# Aniline-containing derivatives of parthenolide: Synthesis and anti-chronic lymphocytic leukaemia activity

**DOI:** 10.1016/j.tet.2020.131631

**Published:** 2020-11-27

**Authors:** Alex S. Quy, Xingjian Li, Louise Male, Tatjana Stankovic, Angelo Agathanggelou, John S. Fossey

**Affiliations:** a School of Chemistry, University of Birmingham, Edgbaston, Birmingham, West Midlands, B15 2TT, UK; b X-Ray Crystallography Facility, School of Chemistry, University of Birmingham, Edgbaston, Birmingham, West Midlands, B15 2TT, UK; c Institute for Cancer and Genomic Sciences, University of Birmingham, Edgbaston, Birmingham, West Midlands, UK

**Keywords:** Parthenolide (PTL), Chronic lymphocytic leukaemia (CLL), Leukemia, 1,4-Conjugate addition, Aniline, Natural product derivatives

## Abstract

Parthenolide exhibits anti-leukaemia activity, whilst its synthetic modification to impart improve drug-like properties, including 1,4-conjugate addition of primary and secondary amines, have previously been used, 1,4-addition of aniline derivatives to parthenolide has not been fully explored. A protocol for such additions to parthenolide is outlined herein. Reaction conditions were determined using tulipane as a model Michael acceptor. Subsequently, aniline-containing parthenolide derivatives were prepared under the optimised conditions and single crystal X-ray diffraction structures were resolved for three of the compounds synthesised. The synthesised derivatives, along with compounds resulting from a side reaction, were tested for their in vitro anti-leukaemia activity using the chronic lymphocytic leukaemia (CLL) MEC1 cell line. Computational studies with the 2RAM protein structure suggested that the activity of the derivatives was independent of their in silico ability to dock with the Cys38 residue of NF-κB.

## Highlights

• Squaric acid-catalysed 1,4-conjugate addition of aniline derivatives to parthenolide.
• Anti-chronic lymphocytic leukaemic activity.
• Crystal structures of some products and by-products.

## Graphical abstract

**Figure:**
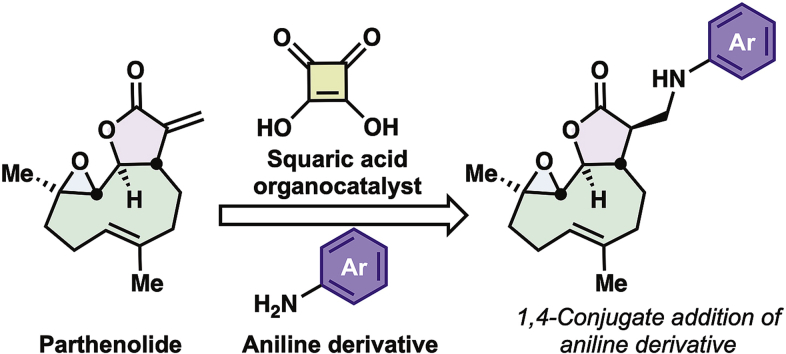

## 1 Introduction

Chronic lymphocytic leukaemia (CLL) is the most common adult leukaemia in western countires [[1], [2], [3]]. Current treatments targeting the disease include ibrutinib which inhibits B cell receptor (BCR) signalling driving CLL cells out of the lymphoid tissues and the replication centres [4] and Venetoclax which inhibits anti-apoptotic signalling by BCL2 [5]. However, these treatments are not curative and disease with high genome instability due to TP53-deficiencies, eventually relapse necessitating alternative approaches [6].

**Fig. 1:**
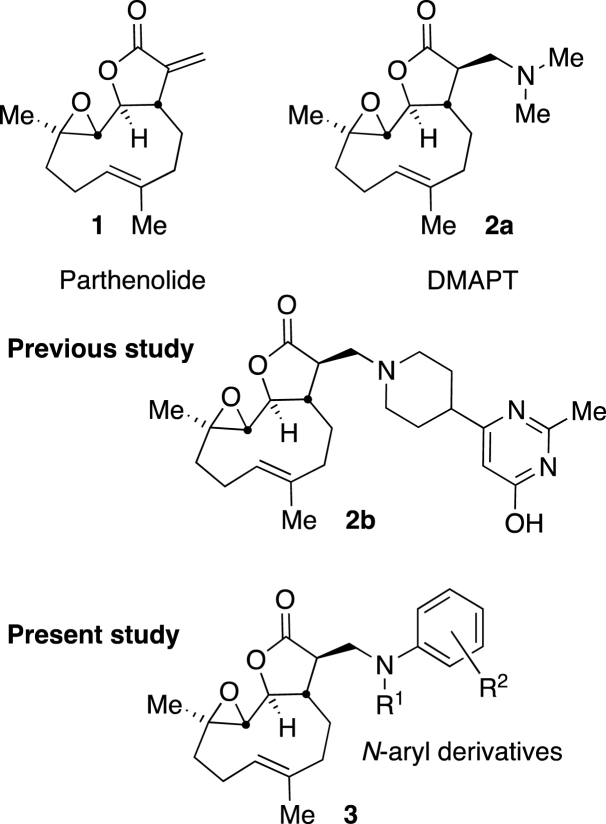
Structures of parthenolide (**1**) and previously reported derivatives thereof DMAPT (**2a**) and compound **2b**. Present study: *N*-Aryl parthenolide derivatives **3**.

Parthenolide (**1**) is a natural product readily extracted from varieties of the plant feverfew (Tanacetum parthenium) [[7], [8], [9], [10], [11], [12], [13], [14], [15]] and has been widely reported to display promising activity against various diseases including types of cancer [[16], [17], [18], [19], [20], [21]]. Mechanistically, parthenolide targets leukaemia cells by depleting glutathione and by alkylating Cys38 of NF-kB to inhibit DNA-binding [22,23]. However, its drug-like properties [24,25] are suboptimal for development as an orally available drug. Great advances in improving the bioavailability of parthenolide have been achieved through relatively straight forward synthetic, one-step, addition to the Michael-acceptor unit of the molecule. The most widely employed manipulation to-date being 1,4-addition of primary and secondary amines, such as the addition of dimethyl amine that results in clean conversion to dimethylaminoparthenolide (DMAPT **2a**, Fig. 1) [[26], [27], [28], [29], [30], [31], [32], [33], [34], [35], [36], [37], [38], [39]]. Notably, the addition of amines leading to **2a** and analogues thereof proceeds with excellent stereoselectivity (typically a single diastereoisomer results) without evidence of any additional by-products arising from epoxide ring-opening. Our team has previously utilised this approach to generate a library of parthenolide derivatives from which compound **2b** (Fig. 1), a promising lead in addressing CLL, was identified [9]. Among reported amine derivatives of parthenolide, to the best of our knowledge [40], there are no reports of analogous *N*-aryl (aniline) derivatives (**3**, Fig. 1). However, a crystal structure report detailing an aniline derivative of 9-α-hydroxy parthenolide that was obtained under zinc(II) chloride Lewis acid-catalysed conditions, indicates feasibility of aniline introduction to related structures [41]. Cytarabine, an *N*-pyrimidine anti-leukaemia drug, has been successfully appended to PTL with a DBU catalyst [42].

Whilst the addition of primary and secondary amines to parthenolide generally proceeds smoothly to give compounds **2** (reactions between **1** and amine in a range of solvents) [9,27,28], it was not possible, in our hands, to so readily react parthenolide with primary or secondary aniline derivatives to form analogous products **3**. As such the challenge to deliver and probe the anti-leukaemic activity of the under explored aniline derivatives of parthenolide (**3**) was embarked upon. This study aims to expand the scope and breadth of compounds in this area, probe new chemical space and test the activity of the compounds against CLL.

## 2 Results and discussion

The 1,4-conjugate addition of primary and secondary aniline derivative to various Michael acceptor motifs has been widely reported [[43], [44], [45], [46], [47], [48], [49]], including related natural product derived lactones [38,41,43,44,50] that have been shown to be amenable to Lewis or Brønsted acid catalysed conjugate addition of anilines. In order to extend the scope of such aniline derivative additions to parthenolide (**1**) a series of model, precedent-informed, reactions were investigated.

Parthenolide (**1**) was obtained by in-house extraction from feverfew and was deemed too valuable to be deployed in the initial development of aniline addition methodology and as such a model substrate was sought. Tulipane (**4**) was selected as a surrogate substrate to probe conditions suitable for conjugate addition of anilines (Table 1). Tulipane, like parthenolide, contains a five-membered lactone ring and bares a double bond that can function as a Michael acceptor.

**Table 1:** Condition screening for the reaction of tulipane (**4**) with aniline (**5a**) to form model product **6**.

Entry	Additive a	Solvent	Temp./°C	Time/h	Yield/%
1	K_2_CO_3_^b^	MeOH or H_2_O	65^e^/100^e^	48	–
2	Et_3_N b	MeOH	65^e^	48	–
3	–	TEAA	20^d^	48	Trace
4	–	TEAA	100	48	18
5	MnCl_2_	H_2_O/MeOH (1:1)	20^d^	48	49
6	MnCl_2_^c^	H_2_O/MeOH (1:1)	20^d^	48	47
7	MnCl_2_	H_2_O/MeOH (1:1)	65^e^	48	66
8	LiBF_4_	Neat	20^d^	24	79
9	LiBF_4_	MeOH	20^d^	24	35
10	LiBF_4_	MeOH	65^e^	24	85
11	Y(NO_3_)_3_·6H_2_O	Neat	20^d^	16	85
12	Squaric acid	H_2_O	20^d^	24	39
13	Squaric acid	H_2_O/MeOH (1:1)	20^d^	24	56
14	Squaric acid	H_2_O/MeOH (1:1)	50	20	95
15	Croconic acid	H_2_O/MeOH (1:1)	20^d^	24	13
16	Croconic acid	H_2_O/MeOH (1:1)	50	24	31

a Unless otherwise stated loaded at 10 mol%.

b 1 equiv. of additive added.

c 20 mol% catalyst loading.

d Refers to an estimated average ambient temperature without climate control.

e Refers to boiling point of the solvent mixture (heated at reflux).

Tulipane (**4**) and aniline (**5a**) in the presence of one equivalent of potassium carbonate or triethylamine failed to deliver desired product **6** (Table 1, entry 1 (water or methanol at reflux) and entry 2 (methanol at reflux) respectively). Triethyl ammonium acetate (TEAA) was prepared according to previously reported procedures [[51], [52], [53], [54], [55]]. At room temperature, use of TEAA as solvent gave only a trace amount of product **6** (Table 1, entry 3); increasing the temperature to 100 °C gave **6** in 18% isolated yield (Table 1, entry 4). The use of manganese(II) chloride as a catalyst [48] at 10 or 20 mol% loading in a water/methanol (1:1) solvent mixture at room temperature (Table 1, entries 5 and 6 respectively) resulted in the formation of **6** in approximately 48% isolated yield (49 and 47%). Increasing the temperature to 65 °C (reflux, 10 mol% loading, Table 1, entry 7) improved the yield to 66%. Under solvent-free conditions (neat) with lithium tetrafluoroborate (10 mol%) at room temperature for 24 h, compound **6** was formed in 79% yield (Table 1, entry 8) [45]. Conducting the reaction in methanol at room temperature resulted in 35% yield, whereas heating at reflux gave a promising 85% isolated yield (Table 1, entries 9 and 10 respectively). Yttrium(III) nitrate hexahydrate was used at 10 mol% under solvent-free conditions at room temperature giving compound **6** in 85% isolated yield (Table 1, entry 11) [56]. Azizi *et al*. showed that squaric acid was an excellent organocatalyst for the addition of aromatic amines (and thiols) to Michael acceptors under aqueous conditions [46]. The formation of **6** under control of squaric acid as catalyst (10 mol%) in water at room temperature proceeded in 39% yield (Table 1, entry 12). When a mixture of water and methanol (1:1) was used as solvent at room temperature the yield was 56% which was further improved to 95% upon heating the reaction mixture to 50 °C (Table 1, entry 13 (24 h) and entry 14 (20 h) respectively). In comparison, croconic acid as catalyst (10 mol%) under similar conditions at room temperature or 50 °C gave product **6** in 13 and 31% isolated yield respectively (Table 1, entries 15 and 16).

**Scheme 1:**
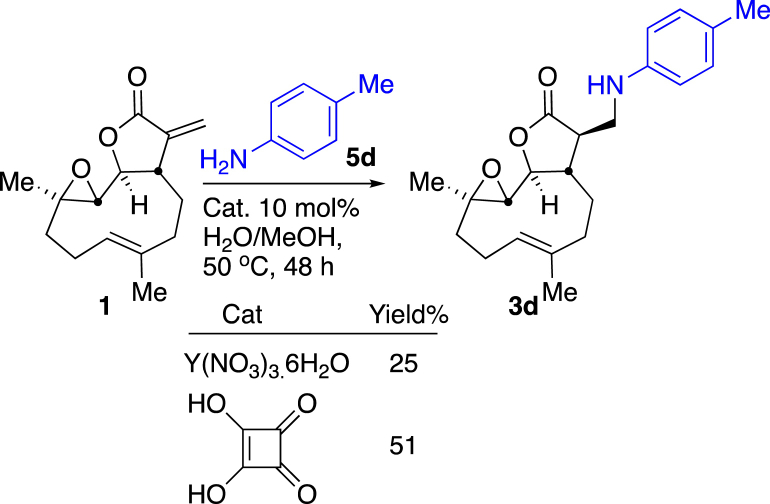
Evaluation of yttrium(III) nitrate hexahydrate and squaric acid as catalysts for the synthesis of compound **3d** from *p*-toluidine (**5d**) and parthenolide (**1**).

From model reactions of tulipane (**4**) and aniline (**5a**) detailed in Table 1, yttrium(III) nitrate hexahydrate and squaric acid were selected as catalysts for evaluation of their suitability to facilitate the addition of *p*-toluidine (4-methylaniline, **5d**) to parthenolide (**1**). *p*-Toluidine was used in these test reactions as it allowed ready reaction monitoring by proton NMR spectroscopy. Use of these catalysts at 10 mol% at 50 °C in a water/methanol (1:1) solvent system gave sluggish conversion to the desired product, parthenolide derivate **3d**: after 48 h yttrium(III) nitrate hexahydrate gave 25% yield, squaric acid performed better with a 51% isolated yield of product **3d** (Scheme 1). Hence, squaric acid as catalyst (10 mol%) at 50 °C for 48 h in a mixture of water and methanol (1:1) as solvent was chosen as the most suitable conditions for synthesis of a range of aniline derivatives of parthenolide.

Under the squaric acid-catalysed aniline addition conditions described above, a range of primary and *N*-methylated secondary anilines (Table 2, entries 1 to 11 and 12 to 14 respectively) were tested. Based on moderate to good isolated yields, no readily discernible correlation of electronic properties of the aniline derivatives used as starting materials to reaction outcomes was observed. The best yields (>70%) were obtained for the use of aniline, *p*-hydroxy aniline and *o*-hydroxy aniline, resulting in products **3a**-**c** in 72–77% isolated yield (Table 2, entries 1 to 3). Primary aniline derivatives bearing *p*-methyl or *p*-methoxy substituents gave products **3d** and **3g** in 51 and 54% isolated yield respectively (Table 2, entries, 4 and 7 respectively). The remaining six primary anilines tested all gave rise to the corresponding desired products, albeit in yields below 40%. Three *N*-methylated secondary anilines were tested as substrates for the same squaric acid-catalysed protocol (Table 2, entries 12 to 14). Unfortunately, the *N*-methylated congener of **3a** (**3l**) was formed in only 8% isolated yield. *p*-Fluoro- and *p*-methoxy analogues did not fare much better giving rise to **3m** and **3n** in 12 and 36% isolated yield respectively. Since tertiary amine derivatives of parthenolide have shown superior pharmaceutical properties (e.g. **2a** and **2b**, Fig. 1) it was reasoned that alternative access to tertiary aniline products should be probed.

**Table 2:** Squaric acid-catalysed reaction of parthenolide (**1**) with aniline-containing substrates to give the corresponding 1,4-conjugate addition products (**3a**-**n**).

Entry	**3**	R	Ar	Yield/%
1	**3a**	H		72
2	**3b**	H		77
3	**3c**	H		75
4	**3d**	H		51
5	**3e**	H		35
6	**3f**	H		9
7	**3g**	H		54
8	**3h**	H		26
9	**3i**	H		28
10	**3j**	H		24
11	**3k**	H		18
12	**3l**	Me		8
13	**3m**	Me		12
14	**3n**	Me		36

**Scheme 2:**
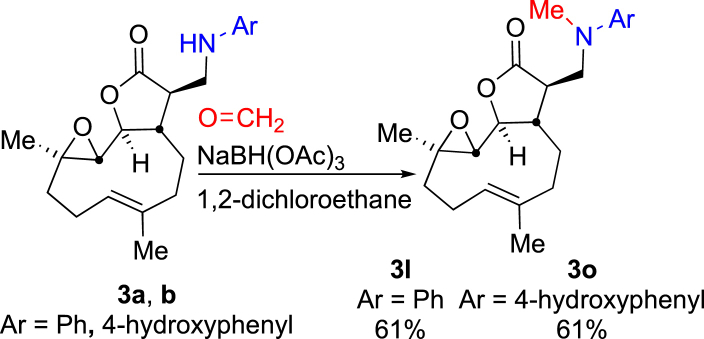
Reductive amination of aniline-containing derivatives of parthenolide (**3a** and **3b**) with formaldehyde to give the corresponding *N*-methylated products **3l** and **3o**.

Since secondary aniline-containing derivatives of parthenolide **3a** and **3b** were available on a larger scale (due to their more ready synthetic access, Table 2) they were selected as substrates upon which to probe the suitability of reductive amination as a strategy to deliver *N*-alkylated tertiary aniline derivatives (Scheme 2). Upon reaction of **3a** or **3b** with formaldehyde with an excess of sodium acetoxy borohydride in 1,2-dichloromethane the corresponding desired products **3l** and **3o** were furnished both in 61% isolated yield. Whilst the yields over the two-steps required to generate them are in the region of 45% this represents a significant improvement over the one-step reaction of *N*-methylated anilines with parthenolide (compare the one-step yield of **3l** of 8% to the two-step reductive amination route giving **3l** in 44% yield overall). Reductive amination is therefore confirmed to be a compatible strategy for *N*-alkylation of secondary aniline-containing derivatives of parthenolide.

**Fig. 2:**
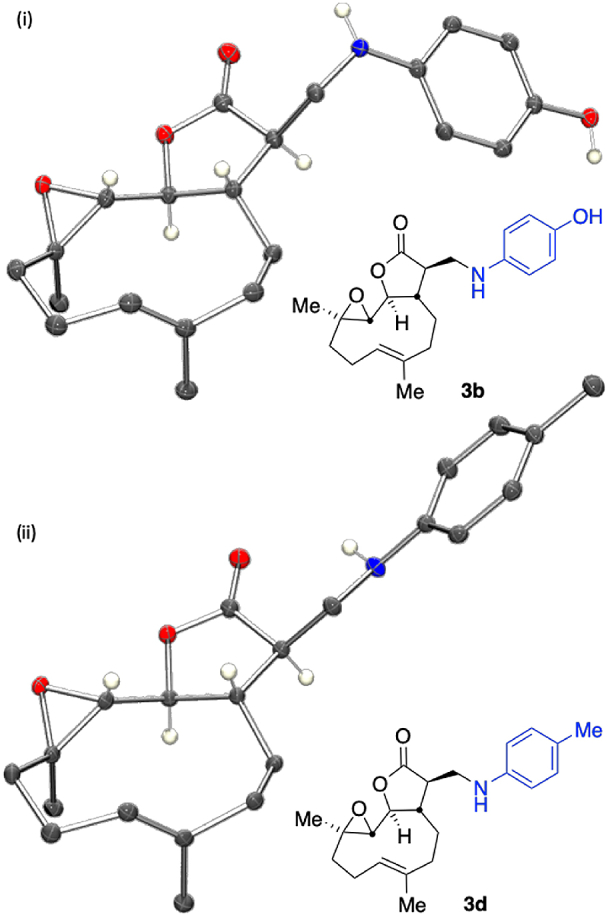
Single crystal XRD structure of (i) **3b** and (ii) **3d**. In the case of 3b (i) a molecule of acetone has been omitted for clarity. Ellipsoid probability 30%, some hydrogen atoms attached to nitrogen or stereogenic centres are shown, remaining hydrogen atoms were omitted for clarity (Ortep III for Windows and PovRay) [57].

The structures and relative stereochemistries of **3b** and **3d** were confirmed by single crystal X-ray diffraction structure determination of the isolated major products (Fig. 2). Thus, the stereoselectivity of aniline addition was determined to be consistent with that previously reported for the more facile addition of primary and secondary amines, i.e. generation of (*R*) stereogenic carbon centres, alpha to the carbonyl in these cases. The absolute stereochemistry of the remaining highly diastereoselective (single isomer detected) squaric acid-catalysed aniline-derivative addition products of Table 2 were assigned by analogy.

**Scheme 3:**
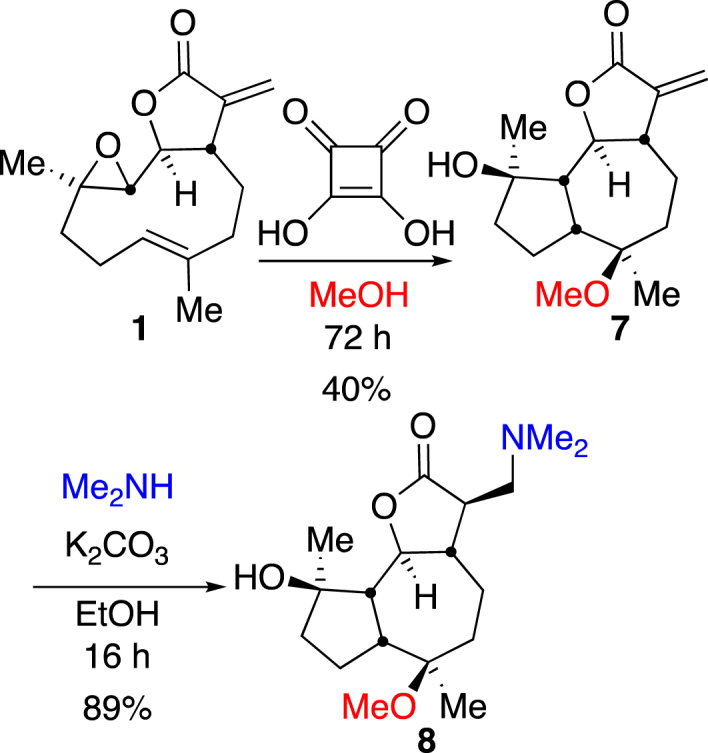
Squaric acid-mediated reaction of parthenolide (**1**) with methanol to give **7**, and its subsequent reaction with dimethylamine to give **8**.

Exposure of parthenolide **1** to acidic conditions in methanol as solvent have been reported to lead to epoxide ring-opening reactions. Although not detected in the reactions of Table 2 unidentified side-products were observed in very low quantities in earlier reactions (Scheme 1). In order to ascertain the nature of the unidentified side-products and to probe whether the squaric acid catalyst could lead to loss of starting material, a blank reaction in the absence of aniline (or derivative thereof) was performed where parthenolide was treated with squaric acid (10 mol%), Scheme 3.

**Fig. 3:**
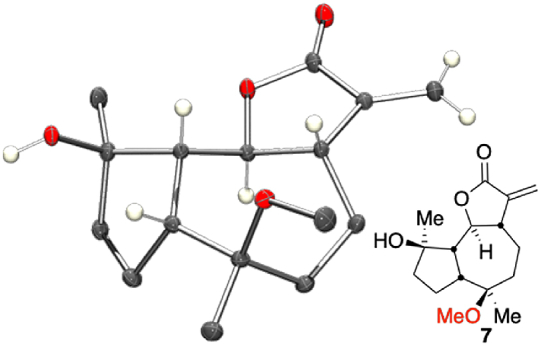
Single crystal XRD structure of one independent molecule of **7**. The asymmetric unit contains two molecules of **7** and one of water. Ellipsoid probability 30%, some hydrogen atoms omitted for clarity (Ortep III for Windows and PovRay) [54].

**Fig. 4:**
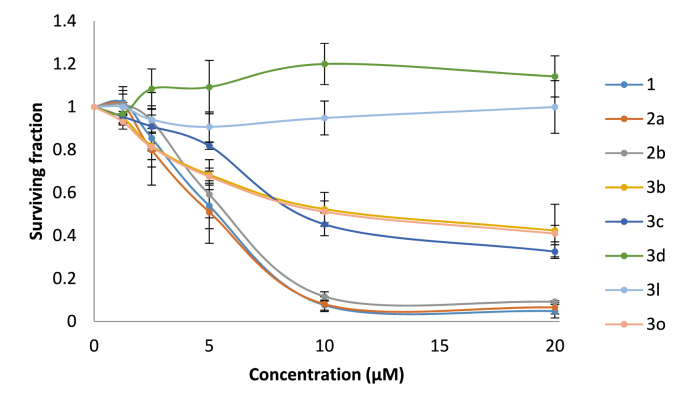
Concentration *versus* activity plots showing the anti-leukaemic activity of *N*-aryl derivatives (**3**) derivatives *versus* parthenolide (**1**) and amine derivatives **2a** and **2b**.

After 72 h, and no further development of product(s) as judged by TLC analysis, the reaction of parthenolide **1** in methanol with squaric acid revealed the formation of *O*-methylated transannular epoxide ring-open product **7** in 40% isolated yield. Compound **7** was not altogether an unexpected result; it has been previously reported to result from the reaction of parthenolide with methanol in acidic conditions and aspects of its biological activity have been probed [[58], [59], [60]]. Furthermore, with an authentic sample of **7** in-hand it was possible to confirm the small amounts of side products formed in earlier experiments were indeed likely to be compound **7**. Compound **7** was analysed by single crystal XRD structure determination which confirmed the identity and relative stereochemistry to be that depicted (Fig. 3). Since compound **7** retains the Michael acceptor motif of the parent compound and attempt to derivatise it with the well-understood dimethyl amine group, in order to furnish **8**, an analogue of **2a**, was made (Scheme 3). Treatment of **7** with dimethylamine and potassium carbonate in ethanol for 16 h resulted in the formation of the expected product **8** in 89% isolated yield. In order to establish the potential applicability of this structural class as anti-leukaemic compounds **7** and **8** were retained and subjected to *in vitro* cell-based assays below (see Fig. 4).

Derivatisation of parthenolide **1** has been widely used to alter its biochemical and pharmacokinetic properties. Inspired by the work of Neelakantan *et al*. in identifying compound **2a** as a promising NF-κB inhibitor [28], the co-authors of this report had previously identified compound **2b** as a potential anti-CLL therapy [8,9], which showed improved drug metabolism and pharmacokinetics (DMPK) properties. To the best of our knowledge the anti-leukaemic activity of aniline-derivatisation on parthenolide has not previously been addressed.

To investigate the anti-leukaemic activity of aniline derivatise **3a**-**o**, the MEC1 chronic lymphocytic leukaemia (CLL) cell line was used. MEC1 is p53-deficient and represents a treatment-refractory model of CLL [61]. The alamarBlue® assay was employed to measure their *in vitro* anti-leukaemic and LC_50_ values were determined using CalcuSyn (Biosoft) software, Table 3. The LC_50_ values of parthenolide (**1**), **2a** and **2b** are consistent with previously published reports (Table 3, 4.6, 3.4 and 5.3 μM, entries 1, 2 and 3 respectively) [9]. The most active aniline derivatives were phenol-containing **3b**, **3c** and **3o** LC_50_ values of 12.5, 11.7 and 12.9 μM respectively (Table 3, entries 5, 6 and 18). Whilst a control experiment confirmed *p*- and *o*-aminophenol were in themselves inactive against CLL, the significance of the phenol functionality to the observed activity in these derivatives remains to be elucidated. Compounds **3a, 3d-m** and **3n** did not show appreciable activity (with LC_50_ determined as >20, >100 and 76.2 μM, Table 3, entry 4, entries 7–16 and 17 respectively). The non-aniline-bearing analogues of **1** and **2a**, compounds **7** and **8** showed reduced cytotoxic activity (Table 3, entries 1 and 2 *versus* entries 19 and 20, LC_50_ values of 4.6 and 3.4 μM versus 14.8 and 14.0 μM respectively).

**Table 3:** Cytotoxic activity against the MEC1 CLL cell line and *in silico* docking score to Cys38 NF-κB.

Entry	Compound	Docking Score(a)	LC_50_ (μM)(b)
1	**1**	−5.3	4.6 ± 0.9
2	**2a**	−4.5	3.4 ± 1.0
3	**2b**	−6.4	5.3 ± 0.5
4	**3a**	−5.2	>20.0
5	**3b**	−5.2	12.5 ± 1.2
6	**3c**	−5.3	11.7 ± 1.3
7	**3d**	−5.2	>100.0
8	**3e**	−5.2	>100.0
9	**3f**	−5.1	>100.0
10	**3g**	−5.1	>100.0
11	**3h**	−5.1	>100.0
12	**3i**	−5.3	>100.0
13	**3j**	−5.3	>100.0
14	**3k**	−5.4	>100.0
15	**3l**	−5	>100.0
16	**3m**	−5	>100.0
17	**3n**	−4.9	76.2 ± 0.3
18	**3o**	−5.1	12.9 ± 1.9
19	**7**	−5.6	14.8

a Best pose calculated from docking each molecule with the p65 subunit of NF-κB with structure code 2RAM centred on Cys38.

b LC_50_ values (CalcuSyn) from dose-response curves generated using low (0–20 μM) or high (0–100 μM) concentrations.

One biological mechanism by which the active compounds may act is through inhibition of the binding between DNA and the p65 domain of NF-κB [22,23] through binding with the Cys38 residue on this protein [59,60]. A series of docking scores were determined using the software Chimera and the structure code 2RAM from the Protein Data Bank. The 3D representations of the parthenolide derivatives were first minimised using MM2 minimisation calculations. Using default settings of Autodock Vina code, and a binding site centred on the Cys38 residue with an 18 Å box of enclosure, all the molecules were analysed with the lowest scoring pose contrasted [62]. The results obtained do not show any significant variance in docking score and as such the aniline derivatisation of parthenolide is unlikely to affect its ability to alkylate NF-kB. Additionally, the retro-Michael addition reaction that occurs in the cell invokes the release of PTL, being the active metabolite. Therefore, docking of the aniline does not directly confirm an inhibition of NF-κB mechanism.

## 3 Conclusion

Successful protocols for the synthesis of aniline-derivatives of parthenolide were developed. The use of squaric acid as an organocatalyst facilitated 1,4-conjugate addition of aniline and aniline derivatives to parthenolide and will permit exploration of hitherto inaccessible chemical space. Aminophenol-containing aniline derivatives of parthenolide **3b**, **3c** and **3o** were the most potent anti-leukaemic compounds among the aniline derivatives tested. Parthenolide has demonstrated potential in a addressing a range of cancer types [30,[63], [64], [65], [66]], attempts to improve its drug-like properties have focused mostly on alkyl amine-derivatisation [9,27,28,31]. Elucidation of methodology herein, that expands the synthetic scope to aniline-derivatives, enables the exploration of hitherto uncharted chemical space in the study of parthenolide, its activity and its drug-like derivatives and analogues.

## 4 Experimental

### 4.1 General procedures

#### 4.1.1 Synthetic chemistry

All commercially available solvents, catalysts and reagents were purchased and used from suppliers without any further purification. Proton NMR spectra were recorded at 400 MHz on a Bruker AVIII400 NMR spectrometer. Carbon NMR spectra are proton decoupled and were recorded at 101 MHz on a Bruker AVIII400 NMR spectrometer at room temperature. Fluorine NMR spectra are proton decoupled and were recorded at 377 MHz on a Bruker AVIII400NMR spectrometer at room temperature. Chemical shifts (δ) were reported in ppm relative to TMS (δ 0.00) for ^1^H NMR and to chloroform (δ 77.16) for ^13^C NMR spectroscopy; coupling constants (*J*) are expressed in Hertz (Hz). The following abbreviations are used for multiplicities: s = singlet, d = doublet, t = triplet, q = quartet, quint = quintet, m = multiplet, pent = pentet, hex = hextet, and br = broad. Mass spectra were recorded on an electrospray MS Waters LCT Time of Flight Mass Spectrometer and with EI (GC/MS) Waters GCT Premier Time of Flight Mass Spectrometer. Infrared Spectra Varian 660-IR FT-IR spectrometer at room temperature using an ATR attachment. Melting points were measured using a StuartTM digital melting point apparatus (SMP10) and reported as a range. Specific optical rotations were recorded on an Optical PolAAr 2001 automatic polarimeter at room temperature. The X-ray crystal structures were determined using an Agilent SuperNova X-ray diffractometer with an Atlas detector (wavelength 1.5418 Å). Column chromatography was carried out using standard flash column chromatography and a Combiflash Rf 200i (stationary phase silica), chromatograms were recorded by evaporative light scattering detector (ELSD) and absorbance at two wavelengths (254 nm and 280 nm). Reactions were monitored by thin layer chromatography (TLC) on Merck silica gel 60 F254 plates. TLC plates were visualised by either UV light with 254 nm/365 nm, a methanolic solution of ninhydrin or with potassium permanganate.

#### 4.1.2 Biological assays

Tissue culture, MEC1 cells were obtained from the American Type Culture Collection (Manassas, VA 20110 USA) and were cultured in RPMI 1640 medium (Sigma-Aldrich, Irvine, UK) with 10% fetal bovine serum (Sigma-Aldrich). The alamarBlue® cytotoxicity assay was conducted by seeding MEC1 cells in triplicate at density of 25000 cells/well in a 96 well plate, final volume of 200 μL [67]. Following treatment with test compound, viability was determined by measuring the reduction of resazurin. Resazurin solution was added to each well at a final concentration of 50 μg/mL and incubated for 3 h at 37 °C with 5% CO_2_. Reduction of resazurin was determined by measuring absorbance at 590 nm using a PheraSTAR FS plate reader (BMG Labtech). Cell viability was calculated as a fraction of the untreated cells after subtracting background fluorescence of resazurin in media only. Data is presented as the mean of five independent experiments and significance was determined by Student’s t-test.

#### 4.1.3 General procedure A: synthesis of aryl aminoparthenolide derivatives

To a 1:1 water-methanol (0.2 M) solution of α,β-unsaturated ester (1 equiv.), the corresponding aniline (1.2 equiv.) and squaric acid (10 mol%.) were added. The resulting solution was stirred at 50 °C for 48 h. The solution was cooled to room temperature and the solvent was removed in vacuo. The residual was extracted with dichloromethane (3 × 25 mL), the combined organic layers were washed with brine and then dried over anhydrous magnesium sulphate, filtered, and solvent removed *in vacuo*. The product was isolated by flash chromatography over silica gel.

#### 4.1.4 General procedure B: synthesis of tertiary aniline parthenolide derivatives from secondary aniline parthenolide derivatives

To a solution of aniline secondary derivative (1 equiv.) in 1,2-dichloroethene (10 mL), formaldehyde (2 equiv.) and sodium triacetoxyborohydride (3 equiv.) were added. The reaction mixture was stirred under nitrogen protection at room temperature for 48 h and quenched with saturated aqueous sodium bicarbonate. The solvent was removed *in vacuo* and the residual was extracted with ethyl acetate. The combined organic solution was dried over anhydrous magnesium sulphate, filtered and concentrated *in vacuo*. Further purification was carried out by flash chromatography over silica gel.

### 4.2 Chemical synthesis

#### 4.2.1 Synthesis of 3a (3R,3aS,9aR,10aR,10bS,E)-6,9a-Dimethyl-3-((phenylamino)methyl)-3a,4,5,8,9,9a,10a,10b-octahydrooxireno[2′,3’:9,10]cyclodeca[1,2-b]furan-2(3H)-one

Following general procedure A, a mixture of parthenolide (150 mg, 0.605 mmol), aniline (75 mg, 0.80 mmol) and squaric acid (7 mg, 0.06 mmol), was stirred at 50 °C in 1:1 water-methanol (20 mL) for 48 h. The title compound was obtained as a pale-yellow oil in 72% yield (148 mg, 0.434 mmol). **Rf** = 0.49 (hexane/ethyl acetate, 50%:50%); ^**1**^**H NMR** (400 MHz, CDCl_3_): δ 7.21–7.17 (2H, m, ArH), 6.74 (1H, t, *J* 7.3, ArH), 6.65 (2H, dd, *J* 8.6 & 0.9, ArH), 5.12 (1H, dd, *J* 12.0 & 2.2, CH), 4.44 (1H, br s, NH), 3.84 (1H, t, *J* 9.0, CH), 3.58 (1H, dd, *J* 13.8 & 3.9, CH_2_), 3.39 (1H, dd, *J* 13.8 & 6.7, CH_2_), 2.68 (1H, d, *J* 9.1, CH, epoxide moiety), 2.60–2.52 (1H, m, CH), 2.43–2.26 (2H, m), 2.18–2.08 (3H, m), 2.07–1.93 (2H, m), 1.76–1.66 (1H, m, CH_2_), 1.68 (3H, s, Me), 1.27 (3H, s, Me), 1.20 (1H, td, J 15.9 & 3.0 CH_2_); ^**13**^**C NMR** (101 MHz, CDCl_3_): δ 176.5 (C=O), 147.8 (Cq), 134.3 (Cq), 129.4 (CH), 125.3 (CH), 118.2 (CH), 113.3 (CH), 82.6 (CH), 66.2 (CH, epoxide moiety), 61.6 (Cq), 47.5 (CH), 46.8 (CH), 42.0 (CH_2_), 41.0 (CH_2_), 36.6 (CH_2_), 30.1 (CH_2_), 24.1 (CH_2_), 17.2 (CH_3_), 16.9 (CH_3_); **IR** (neat, cm^−1^) 1761 (C=O); **HRMS** (ES+) m/z: [M+Na]^+^ calc. for C_21_H_27_NO_3_Na^+^: 364.1889, found: 364.1887; **[α]**_**D**_^**20**^ = −0.26 (c 10.0, CHCl_3_).

#### 4.2.2 Synthesis of **3b** (3R,3aS,9aR,10aR,10bS,E)-3-(((4-Hydroxyphenyl)amino)methyl)-6,9a-dimethyl-3a,4,5,8,9,9a,10a,10b-octahydrooxireno[2′,3’:9,10]cyclodeca[1,2-b]furan-2(3H)-one (3b)

Following general procedure A, a mixture of parthenolide (200 mg, 0.806 mmol), 4-aminophenol (119 mg, 1.06 mmol) and squaric acid (9 mg, 0.08 mmol), was stirred at 50 °C in 1:1 water-methanol (20 mL) for 48 h. The title compound was obtained as a colourless solid in 77% yield (223 mg, 0.625 mmol). **Rf** = 0.46 (hexane/ethyl acetate, 50%:50%); ^**1**^**H NMR** (400 MHz, CDCl_3_): δ 6.71 (2H, d, *J* 8.7, ArH), 6.56 (2H, d, *J* 8.7, ArH), 5.15 (1H, dd, *J* 11.8 & 2.0, CH), 3.83 (1H, t, *J* 9.0, CH), 3.48 (1H, dd, *J* 13.6 & 3.8, CH_2_), 3.29 (1H, dd, *J* 13.5 & 7.2, CH_2_), 2.70 (1H, d, *J* 9.0, CH, epoxide moiety), 2.59–2.50 (1H, m, CH), 2.44–2.24 (2H, m), 2.19–2.07 (3H, m), 2.04–1.88 (2H, m), 1.74–1.61 (1H, m, CH_2_), 1.67 (3H, s, Me), 1.27 (3H, s, Me), 1.18 (1H, td, *J* 12.8 & 5.8, CH_2_); ^**13**^**C NMR** (101 MHz, CDCl_3_): δ 176.9 (C=O), 148.9 (Cq), 141.2 (Cq), 134.4 (Cq), 125.2 (CH), 116.4 (CH), 115.5 (CH), 82.6 (CH), 66.3 (CH, epoxide), 61.9 (Cq), 47.0 (CH), 46.9 (CH), 43.6 (CH_2_), 41.0 (CH_2_), 36.5 (CH_2_), 30.1 (CH_2_), 24.1 (CH_2_), 17.2 (CH_3_), 16.9 (CH_3_); **IR** (neat, cm^−1^) 3345 (OH), 1758 (C=O); **HRMS** (ES+) m/z: [M+Na]^+^ calc. for C_21_H_27_NO_4_Na^+^: 380.1838, found: 380.1831; **mp**: 85–87 °C; **[α]**_**D**_^**20**^ = −7.42° (c 7.0, CHCl_3_).

#### 4.2.3 Synthesis of **3c** (3R,3aS,9aR,10aR,10bS,E)-3-(((2-Hydroxyphenyl)amino)methyl)-6,9a-dimethyl-3a,4,5,8,9,9a,10a,10b-octahydrooxireno[2′,3’:9,10]cyclodeca[1,2-b]furan-2(3H)-one

Following general procedure A, a mixture of parthenolide (100 mg, 0.403 mmol), 2-aminophenol (88 mg, 0.40 mmol) and squaric acid (5 mg, 0.04 mmol), was stirred at 50 °C in 1:1 water-methanol (20 mL) for 48 h. The title compound was obtained as a colourless solid in 75% yield (107 mg, 0.300 mmol). **Rf** = 0.40 (hexane/ethyl acetate, 50%:50%); ^**1**^**H NMR** (400 MHz, CDCl_3_): δ 6.88 (2H, m, ArH), 6.75 (2H, m, ArH), 5.10 (1H, dd, *J* 11.8 & 1.8, CH), 3.85 (1H, t, *J* 9.0, CH), 3.56 (1H, dd, *J* 13.7 & 4.0, CH_2_), 3.44 (1H, dd, *J* 13.7 & 6.2, CH_2_), 2.71 (1H, d, *J* 8.9, CH, epoxide moiety), 2.65–2.56 (1H, m, CH), 2.43–2.22 (2H, m), 2.20–2.08 (3H, m), 2.05–1.90 (2H, m), 1.72–1.64 (1H, m, CH_2_), 1.68 (3H, s, Me), 1.28 (3H, s, Me), 1.20 (1H, td, *J* 12.8 & 5.9, CH_2_); ^**13**^**C NMR** (101 MHz, CDCl_3_): δ 176.7 (C=O), 144.8 (Cq), 136.4 (Cq), 134.4 (Cq), 125.2 (CH), 121.3 (CH), 119.1 (CH), 115.0 (CH), 113.2 (CH), 82.6 (CH), 66.3 (CH, epoxide moiety), 61.8 (Cq), 47.7 (CH), 46.9 (CH), 42.7 (CH_2_), 41.0 (CH_2_), 36.5 (CH_2_), 30.1 (CH_2_), 24.1 (CH_2_), 17.2 (CH_3_), 16.9 (CH_3_); **IR** (neat, cm^−1^) 3386 (OH), 1764 (C=O); **HRMS** (ES+) m/z: [M+H]+ calc. for C_21_H_28_NO_4_^+^: 358.2018, found: 358.2016; **mp**: 195–198 °C; **[α]**_**D**_^**20**^ = −1.08° (c 2.3, CHCl_3_).

#### 4.2.4 Synthesis of **3d** (3R,3aS,9aR,10aR,10bS,E)-6,9a-dimethyl-3-((p-tolylamino)methyl)-3a,4,5,8,9,9a,10a,10b-octahydrooxireno[2′,3’:9,10]cyclodeca[1,2-b]furan-2(3H)-one

Following general procedure A, a mixture of parthenolide (100 mg, 0.403 mmol), p-toluidine (52 mg, 0.48 mmol) and squaric acid (5 mg, 0.04 mmol) was stirred at 50 °C in 1:1 water-methanol (2 mL) for 48 h. The title compound was obtained as a white solid in 51% yield (73 mg). **Rf** = 0.65 (hexane/ethyl acetate, 50%:50%); ^**1**^**H NMR** (400 MHz, CDCl_3_) δ 7.01 (2H, d, *J* 8.2), 6.58 (2H, d, *J* 8.4), 5.13 (1H, d, *J* 8.9), 4.28 (1H, s), 3.84 (1H, t, *J* 9.1), 3.56 (1H, d, *J* 13.8), 3.36 (1H, dd, *J* 13.7 & 6.8), 2.69 (1H, d, *J* 8.9), 2.60–2.51 (1H, m), 2.43–2.27 (2H, m), 2.24 (3H, s), 2.20–1.93 (5H, m), 1.76–1.60 (1H, m), 1.69 (3H, s), 1.28 (3H, s), 1.19 (1H, td, *J* 13.4 & 5.8); ^**13**^**C NMR** (101 MHz, CDCl_3_) δ 176.5, 145.5, 134.3, 129.9, 127.6, 125.3, 113.6, 82.5, 66.2, 61.6, 47.4, 46.8, 42.5, 41.1, 36.6, 30.1, 24.1, 20.4, 17.2, 16.9; **IR** (neat, cm^−1^) 3363 (NH), 1747 (C=O); **HRMS** (TOF MS ES+) m/z: [M+H]^+^ calc. for C_22_H_30_NO_3_^+^ 356.2224, found 356.2226; **mp** 200–203 °C; **[α]**_**D**_^**22**^ **=** −12.81° (c 2.0, CH_2_Cl_2_).

#### 4.2.5 Synthesis of **3e** (3R,3aS,9aR,10aR,10bS,E)-3-(((4-Fluorophenyl)amino)methyl)-6,9a-dimethyl-3a,4,5,8,9,9a,10a,10b-octahydrooxireno[2′,3’:9,10]cyclodeca[1,2-b]furan-2(3H)-one

Following general procedure A, a mixture of parthenolide (100 mg, 0.403 mmol), 4-fluoroaniline (54 mg, 0.48 mmol) and squaric acid (5 mg, 0.04 mmol) was stirred at 50 °C in 1:1 water-methanol (2 mL) for 48 h. The title compound was obtained as a white solid in 35% yield (51 mg). **Rf** = 0.57 (hexane/ethyl acetate, 50%:50%); ^**1**^**H NMR** (400 MHz, CDCl_3_) δ 6.97–6.86 (2H, m), 6.66–6.56 (2H, m), 5.15 (1H, d, *J* 12.3), 4.37 (1H, s), 3.87 (1H, t, *J* 9.0), 3.54 (1H, dd, *J* 13.5 & 3.8), 3.34 (1H, dd, *J* 13.5 & 7.1), 2.70 (1H, d, *J* 8.9), 2.56 (1H, ddd, *J* 12.4, 7.1 & 3.8), 2.47–2.28 (2H, m), 2.22–1.91 (5H, m), 1.81–1.67 (1H, m), 1.70 (3H, s), 1.29 (3H, s), 1.27–1.14 (1H, m); ^**13**^**C NMR** (101 MHz, CDCl_3_) δ 176.4, 157.5, 144.0, 134.2, 125.4, 116.0, 115.8, 114.5, 114.4, 82.6, 66.2, 61.6, 47.3, 46.9, 43.0, 41.1, 36.5, 30.1, 24.1, 17.2, 16.9; ^**19**^**F** **NMR** (377 MHz, CDCl_3_) δ −126.83; **IR** (neat, cm^−1^) 3371 (NH), 1748 (C=O); **HRMS** (TOF MS ES+) m/z: [M+H]^+^ calc. for C_21_H_27_NO_3_^+^ 360.1974, found 360.1975; **mp** 121–124 °C; **[α]**_**D**_^**22**^ **=** −19.39° (c 0.5, CH_2_Cl_2_).

#### 4.2.6 Synthesis of **3f** (3R,3aS,9aR,10aR,10bS,E)-3-(((4-Bromophenyl)amino)methyl)-6,9a-dimethyl-3a,4,5,8,9,9a,10a,10b-octahydrooxireno[2′,3’:9,10]cyclodeca[1,2-b]furan-2(3H)-one

Following general procedure A, a mixture of parthenolide (100 mg, 0.403 mmol), 4-bromoaniline (83 mg, 0.48 mmol) and squaric acid (5 mg, 0.04 mmol) was stirred at 50 °C in 1:1 water-methanol (2 mL) for 48 h. The title compound was obtained as a white solid in 9% yield (15 mg). **Rf** = 0.57 (hexane/ethyl acetate, 50%:50%); ^**1**^**H NMR** (400 MHz, CDCl_3_) δ 7.31–7.23 (2H, m), 6.58–6.49 (2H, m), 5.14 (1H, d, *J* 12.1), 4.48 (1H, t, *J* 6.8), 3.91–3.82 (1H, m), 3.59–3.52 (1H, m) 3.39–3.31 (1H, m), 2.69 (1H, d, *J* 8.9), 2.59–2.52 (1H, m), 2.45–2.28 (2H, m), 2.21–1.93 (5H, m), 1.79–1.70 (1H, m), 1.70 (3H, s), 1.29 (3H, s), 1.30–1.16 (1H, m); ^**13**^**C NMR** (101 MHz, CDCl_3_) δ 176.3, 146.7, 134.2, 132.2, 125.5, 114.9, 109.9, 82.6, 66.2, 61.6, 60.4, 47.4, 46.9, 42.1, 41.1, 36.5, 30.1, 24.1, 17.2, 16.9, 14.2; **IR** (neat, cm^−1^) 3380 (NH), 1760 (C=O); **HRMS** (TOF MS ASAP+) m/z: [M+H]^+^ calcd. for C_21_H_27_BrNO_3_^+^ 420.1174 found 420.1179; **mp** 182–184 °C; **[α]**_**D**_^**22**^ = 9.70° (c 0.5, CH_2_Cl_2_).

#### 4.2.7 Synthesis of **3g** (3R,3aS,9aR,10aR,10bS,E)-3-(((4-Methoxyphenyl)amino)methyl)-6,9a-dimethyl-3a,4,5,8,9,9a,10a,10b-octahydrooxireno[2′,3’:9,10]cyclodeca[1,2-b]furan-2(3H)-one

Following general procedure A, a mixture of parthenolide (100 mg, 0.403 mmol), 4-methoxyaniline (60 mg, 0.48 mmol) and squaric acid (5 mg, 0.04 mmol) was stirred at 50 °C in 1:1 water-methanol (2 mL) for 48 h. The title compound was obtained as a white solid in 54% yield (81 mg). **Rf** = 0.50 (hexane/ethyl acetate, 50%:50%); ^**1**^**H NMR** (400 MHz, CDCl_3_) δ 6.84–6.75 (2H, m), 6.68–6.60 (2H, m), 5.13 (1H, d, *J* 12.2), 4.21 (1H, s), 3.85 (1H, t, *J* 9.0), 3.75 (3H, s), 3.53 (1H, dd, *J* 13.5 & 3.8), 3.33 (1H, dd, *J* 13.5 & 7.0), 2.70 (1H, d, *J* 8.9), 2.56 (1H, ddd, *J* 12.3, 6.9 & 3.8), 2.45–2.26 (2H, m), 2.21–1.91 (5H, m), 1.78–1.65 (1H, m), 1.69 (3H, s), 1.28 (3H, s), 1.20 (1H, td, *J* 13.0 & 5.9); ^**13**^**C NMR** (101 MHz, CDCl_3_) δ 176.6, 152.7, 141.8, 134.3, 125.3, 115.0, 82.6, 66.2, 61.6, 55.8, 47.2, 46.9, 43.4, 41.1, 36.6, 30.0, 24.1, 17.2, 16.9; **IR** (neat, cm^−1^) 3365 (NH), 1747 (C=O); **HRMS** (TOF MS ES+) m/z: [M+H]^+^ calcd. for C_22_H_30_NO_4_^+^ 372.2175, found 372.2174; **mp** 145–147 °C; **[α]**_**D**_^**22**^ = −16.62° (c 1.0, CH_2_Cl_2_).

#### 4.2.8 Synthesis of **3h** (3R,3aS,9aR,10aR,10bS,E)-3-(((3,5-Dimethoxyphenyl)amino)methyl)-6,9a-dimethyl-3a,4,5,8,9,9a,10a,10b-octahydrooxireno[2′,3’:9,10]cyclodeca[1,2-b]furan-2(3H)-one

Following general procedure A, a mixture of parthenolide (100 mg, 0.403 mmol), 3,5-dimethoxyaniline (74 mg, 0.48 mmol) and squaric acid (5 mg, 0.04 mmol) was stirred at 50 °C in 1:1 water-methanol (2 mL) for 48 h. The title compound was obtained as a white solid in 26% yield (42 mg). **Rf** = 0.50 (hexane/ethyl acetate, 50%:50%); ^**1**^**H NMR** (400 MHz, CDCl_3_) δ 5.91 (1H, t, *J* 2.1), 5.83 (2H, d, J 2.1), 5.13 (1H, dd, *J* 11.9 & 2.3), 4.47 (1H, s), 3.86 (1H, t, *J* 9.0), 3.75 (6H, s), 3.56 (1H, dd, *J* 14.5 & 3.3), 3.36 (1H, dd, *J* 13.8 & 6.8), 2.69 (1H, d, *J* 8.9), 2.57 (1H, ddd, *J* 12.3, 6.8 & 3.7), 2.46–2.27 (2H, m), 2.21–1.94 (5H, m), 1.80–1.70 (1H, m), 1.69 (3H, s), 1.29 (3H, s), 1.20 (1H, td, *J* 13.4 & 6.3); ^**13**^**C NMR** (101 MHz, CDCl_3_) δ 176.4, 161.9, 149.7, 134.3, 125.4, 92.1, 90.4, 82.6, 66.2, 61.6, 55.2, 47.5, 46.8, 41.8, 41.0, 36.6, 30.1, 24.1, 17.2, 16.9; **IR** (neat, cm^−1^) 3389 (NH), 1761 (C=O); **HRMS** (TOF MS ES+) m/z: [M+H]^+^ calcd. for C_23_H_32_NO_5_^+^ 402.2280, found 402.2285; **mp** 110–112 °C; **[α]**_**D**_^**22**^ = −5.02° (c 2.0, CH_2_Cl_2_).

#### 4.2.9 Synthesis of **3i** (3R,3aS,9aR,10aR,10bS,E)-3-(((3,5-Dimethylphenyl)amino)methyl)-6,9a-dimethyl-3a,4,5,8,9,9a,10a,10b-octahydrooxireno[2′,3’:9,10]cyclodeca[1,2-b]furan-2(3H)-one

Following general procedure A, a mixture of parthenolide (100 mg, 0.403 mmol), 3,5-dimethylaniline (54 mg, 0.48 mmol) and squaric acid (5 mg, 0.04 mmol) was stirred at 50 °C in 1:1 water-methanol (2 mL) for 48 h. The title compound was obtained as a white solid in 28% yield (42 mg). **Rf** = 0.62 (hexane/ethyl acetate, 50%:50%); ^**1**^**H NMR** (400 MHz, CDCl_3_) δ 6.41 (1H, s), 6.28 (2H, s), 5.12 (1H, dd, *J* 12.1 & 2.3), 4.27 (1H, s), 3.84 (1H, t, *J* 9.0), 3.58 (1H, dd, *J* 13.8 & 3.8), 3.39 (1H, dd, *J* 13.8 & 6.3), 2.68 (1H, d, *J* 8.9), 2.55 (1H, ddd, *J* 12.3, 6.3 & 3.8), 2.45–2.27 (2H, m), 2.24 (6H, s), 2.20–1.95 (5H, m), 1.79–1.66 (1H, m), 1.70 (3H, s), 1.29 (3H, s), 1.19 (1H, td, *J* 13.0 & 6.0); ^**13**^**C NMR** (101 MHz, CDCl_3_) δ 176.4, 147.9, 139.1, 134.3, 125.4, 120.2, 111.3, 82.5, 66.2, 61.6, 47.8, 46.7, 42.0, 41.0, 36.6, 30.1, 24.1, 21.5, 17.2, 16.9; **IR** (neat, cm^−1^) 3382 (NH), 1755 (C=O); **HRMS** (TOF MS ES+) m/z: [M+H]^+^ calcd. for C_23_H_32_NO_3_^+^ 370.2383, found 370.2382; **mp** 196–197 °C; **[α]**_**D**_^**22**^ = 11.08° (c 0.5, CH_2_Cl_2_).

#### 4.2.10 Synthesis of **3j** (3R,3aS,9aR,10aR,10bS,E)-3-(((3-Methoxyphenyl)amino)methyl)-6,9a-dimethyl-3a,4,5,8,9,9a,10a,10b-octahydrooxireno[2′,3’:9,10]cyclodeca[1,2-b]furan-2(3H)-one

Following general procedure A, a mixture of parthenolide (80 mg, 0.32 mmol), 3-methoxyaniline (48 mg, 0.38 mmol) and squaric acid (4 mg, 0.03 mmol) was stirred at 50 °C in 1:1 water-methanol (2 mL) for 48 h. The title compound was obtained as a white solid in 24% yield (28 mg). **Rf** = 0.52 (hexane/ethyl acetate, 50%:50%); ^**1**^**H NMR** (400 MHz, CDCl_3_) δ 7.10 (1H, t, *J* 8.1), 6.31 (1H, dd, *J* 8.2 & 2.4), 6.26 (1H, dd, *J* 8.0 & 2.2), 6.20 (1H, t, *J* 2.3), 5.13 (1H, d, *J* 12.2 & 2.4), 4.43 (1H, s), 3.85 (1H, t, *J* 9.0), 3.77 (3H, s), 3.58 (1H, dd, *J* 14.1 & 3.6), 3.39 (1H, dd, *J* 13.8 & 6.7), 2.69 (1H, d, *J* 8.9), 2.56 (1H, ddd, *J* 12.3, 6.7 & 3.8), 2.46–2.25 (2H, m), 2.20–1.94 (5H, m), 1.79–1.66 (1H, m), 1.69 (3H, s), 1.29 (3H, s), 1.27–1.14 (1H, m); ^**13**^**C NMR** (101 MHz, CDCl_3_) δ 176.4, 161.0, 149.2, 134.3, 130.2, 125.4, 106.3, 103.4, 99.4, 82.6, 66.2, 61.6, 55.2, 47.6, 46.8, 41.9, 41.0, 36.6, 30.1, 24.1, 17.2, 16.9; **IR** (neat, cm^−1^) 2930 (NH), 1762 (C=O); **HRMS** (TOF MS ES+) m/z: [M+H]^+^ calcd. for C_22_H_30_NO_4_^+^ 372.2175, found 372.2177; **mp** 154–156 °C; **[α]**_**D**_^**22**^ = −17.31° (c 1.0, CH_2_Cl_2_).

#### 4.2.11 Synthesis of **3k** (3R,3aS,9aR,10aR,10bS,E)-6,9a-dimethyl-3-((m-tolylamino)methyl)-3a,4,5,8,9,9a,10a,10b-octahydrooxireno[2′,3’:9,10]cyclodeca[1,2-b]furan-2(3H)-one

Following general procedure A, a mixture of parthenolide (80 mg, 0.32 mmol), m-toluidine (42 mg, 0.38 mmol) and squaric acid (4 mg, 0.03 mmol) was stirred at 50 °C in 1:1 water-methanol (2 mL) for 48 h. The title compound was obtained as a white solid in 18% yield (20 mg). **Rf** = 0.64 (hexane/ethyl acetate, 50%:50%); ^**1**^**H NMR** (400 MHz, CDCl_3_) δ 7.08 (1H, t, J 7.5), 6.57 (1H, d, *J* 7.2), 6.50–6.43 (2H, m), 5.12 (1H, dd, *J* 12.2 & 3.8), 4.35 (1H, s), 3.85 (1H, t, *J* 9.0), 3.58 (1H, dd, *J* 13.8 & 3.9), 3.40 (1H, dd, *J* 13.8 & 6.6), 2.68 (1H, d, *J* 8.9), 2.56 (1H, ddd, *J* 12.2, 6.5 & 3.8), 2.45–2.30 (2H, m), 2.28 (3H, s), 2.20–1.95 (5H, m), 1.79–1.66 (1H, m), 1.69 (3H, s), 1.29 (3H, s), 1.19 (1H, td, *J* 13.0 & 5.9); ^**13**^**C NMR** (101 MHz, CDCl_3_) δ 176.4, 147.8, 139.3, 134.3, 129.3, 125.4, 119.2, 114.2, 110.4, 82.6, 66.2, 61.6, 47.6, 46.8, 42.0, 41.0, 36.6, 30.1, 24.1, 21.6, 17.2, 16.9; **IR** (neat, cm^−1^) 3385 (NH), 1761 (C=O); **HRMS** (TOF MS ES+) m/z: [M+H]^+^ calc. for C_22_H_30_NO_3_^+^ 356.2224, found 356.2230; **mp** 154–156 °C; **[α]**_**D**_^**22**^ = −7.62° (c 0.5, CH_2_Cl_2_).

#### 4.2.12 Synthesis of **3l** (3R,3aS,9aR,10aR,10bS,E)-6,9a-dimethyl-3-((methyl(phenyl)amino)methyl)-3a,4,5,8,9,9a,10a,10b-octahydrooxireno[2′,3’:9,10]cyclodeca[1,2-b]furan-2(3H)-one

##### 4.2.12.1 Method (i)

Following general procedure A, a mixture of parthenolide (100 mg, 0.403 mmol), *N*-methylaniline (52 mg, 0.48 mmol) and squaric acid (5 mg, 0.04 mmol) was stirred at 50 °C in 1:1 water-methanol (2 mL) for 48 h. The title compound was obtained as a white solid in 8% yield (9 mg). **Rf** = 0.67 (hexane/ethyl acetate, 50%:50%); ^**1**^**H NMR** (400 MHz, CDCl_3_) δ 7.29–7.22 (2H, m), 6.79–6.70 (3H, m), 5.05 (1H, dd, *J* 11.8 & 2.3), 4.01 (1H, dd, *J* 15.4 & 4.9), 3.79 (1H, t, *J* 9.0), 3.65 (1H, dd, *J* 15.5 & 6.1), 3.01 (3H, s), 2.74–2.62 (2H, m), 2.41–2.28 (1H, m), 2.19–2.03 (4H, m), 1.96–1.86 (1H, m), 1.79–1.68 (1H, m), 1.64 (3H, s), 1.65–1.53 (1H, m), 1.26 (3H, s), 1.19 (1H, td, *J* 13.0 & 5.9); ^**13**^**C NMR** (101 MHz, CDCl_3_) δ 176.0, 149.0, 134.4, 129.5, 125.0, 117.2, 112.5, 82.3, 66.5, 61.6, 52.1, 48.1, 46.4, 40.9, 39.6, 36.6, 30.4, 24.1, 17.2, 16.9; **IR** (neat, cm^−1^) 1760 (C=O); **HRMS** (TOF MS ES+) m/z: [M+H]^+^ calc. for C_22_H_30_NO_3_^+^ 356.2224, found 356.2232; **mp** 153–156 °C; **[α]**_**D**_^**22**^ = −124.65° (c 1.0, CH_2_Cl_2_).

##### 4.2.12.2 Method (ii)

Following general procedure B, a mixture of aminoparthenolide derivative **3a** (109 mg, 0.320 mmol), formaldehyde (30 mg, 0.96 mmol) and sodium triacetoxyborohydride (112 mg, 0.528 mmol), was stirred at room temperature in dichloroethane (10 mL) for 16 h. The title compound was obtained as a colourless solid in 61% yield (72 mg, 0.20 mmol). ^1^H NMR, ^13^C NMR, melting point and HRMS data all consistent with above.

#### 4.2.13 Synthesis of **3m** (3R,3aS,9aR,10aR,10bS,E)-3-(((4-Fluorophenyl)(methyl)amino)methyl)-6,9a-dimethyl-3a,4,5,8,9,9a,10a,10b-octahydrooxireno[2′,3’:9,10]cyclodeca[1,2-b]furan-2(3H)-one

Following general procedure A, a mixture of parthenolide (80 mg, 0.32 mmol), 4-fluoro-*N*-methylaniline (48 mg, 0.38 mmol) and squaric acid (4 mg, 0.03 mmol) was stirred at 50 °C in 1:1 water-methanol (2 mL) for 48 h. The title compound was obtained as a white solid in 8% yield (9 mg). **Rf** = 0.64 (hexane/ethyl acetate, 50%:50%); ^**1**^**H NMR** (400 MHz, CDCl_3_) δ 7.02–6.91 (2H, m), 6.75–6.65 (2H, m), 5.07 (1H, dd, *J* 12.2 & 2.0), 3.92 (1H, dd, *J* 15.4 & 4.9), 3.80 (1H, t, *J* 9.0), 3.60 (1H, dd, *J* 15.4 & 6.0), 2.97 (3H, s), 2.67 (1H, d, *J* 8.9), 2.63 (1H, ddd, *J* 11.9, 6.0 & 4.9), 2.44–2.28 (1H, m), 2.19–2.06 (4H, m), 1.89 (1H, dd, *J* 15.2 & 6.6), 1.76 (1H, t, *J* 13.0), 1.65 (3H, s), 1.66–1.54 (1H, m), 1.27–1.13 (4H, m); ^**13**^**C NMR** (101 MHz, CDCl_3_) δ 176.0, 145.8, 134.3, 125.1, 115.9, 115.7, 113.9, 113.9, 82.3, 66.5, 61.6, 52.8, 48.1, 46.4, 41.0, 40.1, 36.6, 30.3, 24.1, 17.2, 16.9; ^**19**^**F-NMR** (377 MHz, CDCl_3_) δ −128.2; **IR** (neat, cm^−1^) 1761 (C=O); **HRMS** (TOF MS ASAP+) m/z: [M+H]^+^ calc. for C_22_H_29_FNO_3_^+^ 374.2131, found 374.2141; **mp** 131–134 °C; **[α]**_**D**_^**22**^ = 34.97° (c 1.0, CH_2_Cl_2_).

#### 4.2.14 Synthesis of **3n** (3R,3aS,9aR,10aR,10bS,E)-3-(((4-Methoxyphenyl)(methyl)amino)methyl)-6,9a-dimethyl-3a,4,5,8,9,9a,10a,10b-octahydrooxireno[2′,3’:9,10]cyclodeca[1,2-b]furan-2(3H)-one

Following general procedure A, a mixture of parthenolide (80 mg, 0.32 mmol), 4-methoxy-*N*-methylaniline (53 mg, 0.38 mmol) and squaric acid (4 mg, 0.03 mmol) was stirred at 50 °C in 1:1 water-methanol (2 mL) for 48 h. The title compound was obtained as a white solid in 36% yield (45 mg). **Rf** = 0.64 (hexane/ethyl acetate, 50%:50%); ^**1**^**H NMR** (400 MHz, CDCl_3_) δ 6.90–6.81 (2H, m), 6.79–6.71 (2H, m), 5.08 (1H, dd, *J* 9.8 & 2.1), 3.89 (1H, dd, *J* 15.2 & 4.8), 3.80 (1H, t, *J* 9.2), 3.76 (3H, s), 3.52 (1H, dd, *J* 15.3 & 6.2), 2.93 (3H, s), 2.68 (1H, d, *J* 8.9), 2.62 (1H, ddd, *J* 11.9, 6.0 & 4.8), 2.44–2.28 (1H, m), 2.21–2.07 (4H, m), 1.93 (1H, dd, *J* 14.8 & 6.6), 1.78 (1H, t, *J* 12.7), 1.64 (3H, s), 1.63–1.51 (1H, m), 1.27 (3H, s), 1.25–1.15 (1H, m); ^**13**^**C NMR** (101 MHz, CDCl_3_) δ 176.2, 152.2, 144.0, 134.5, 125.0, 115.0, 114.9, 82.3, 66.5, 61.6, 55.8, 53.2, 48.1, 46.4, 41.0, 40.4, 36.6, 30.3, 24.1, 17.2, 16.9; **IR** (neat, cm^−1^) 1766 (C=O); **HRMS** (TOF MS ASAP+) m/z: [M+H]^+^ calc. for C_23_H_32_NO_4_^+^ 386.2331, found 386.2330; **mp** 146–148 °C; **[α]**_**D**_^**22**^ = 81.72° (c 2.0, CH_2_Cl_2_).

#### 4.2.15 Synthesis of **3o** (3R,3aS,9aR,10aR,10bS,E)-3-(((4-Hydroxyphenyl)(methyl)amino)methyl)-6,9a-dimethyl-3a,4,5,8,9,9a,10a,10b-octahydrooxireno[2′,3’:9,10]cyclodeca[1,2-b]furan-2(3H)-one

Following general procedure B, a mixture of aminoparthenolide derivative **3b** (114 mg, 0.319 mmol), formaldehyde (30 mg, 0.96 mmol) and sodium triacetoxyborohydride (112 mg, 0.528 mmol), was stirred at room temperature in dichloroethane (10 mL) for 16 h. The title compound was obtained as a colourless solid in 61% yield (72 mg, 0.20 mmol). **Rf** = 0.49 (hexane/ethyl acetate, 50%:50%); ^**1**^**H NMR** (400 MHz, CD_3_OD): δ 6.81–6.71 (4H, m, ArH), 5.14 (1H, dd, *J* 12.4 & 2.3, CH), 3.97 (1H, t, J 9.1, CH), 3.83 (1H, dd, *J* 15.0 & 4.9, CH_2_), 3.55 (1H, dd, *J* 15.0 & 6.0, CH_2_), 2.90 (3H, s, Me), 2.81 (1H, d, *J* 9.1, CH, epoxide moiety), 2.80–2.73 (1H, m, CH), 2.51–2.36 (1H, m, CH_2_), 2.32–2.22 (1H, m, CH_2_), 2.19–2.03 (3H, m), 1.91 (1H, dd, *J* 14.6 & 6.3, CH_2_), 1.79 (1H, t, *J* 12.8, CH_2_), 1.71–1.61 (1H, m, CH_2_), 1.68 (3H, s, Me), 1.29 (3H, s, Me), 1.22 (1H, td, *J* 12.7 & 5.9, CH_2_); ^**13**^**C NMR** (101 MHz, CD_3_OD): δ 175.2 (C=O), 148.9 (Cq), 143.6 (Cq), 134.7 (Cq), 124.3 (CH), 115.6 (CH), 82.4 (CH), 66.7 (CH, epoxide moiety), 61.8 (Cq), 52.9 (CH_2_), 46.1 (CH), 40.5 (CH_2_), 39.3 (CH_3_), 36.2 (CH_2_), 29.4 (CH_2_), 23.5 (CH_2_), 16.0 (CH_3_), 15.7 (CH_3_); **IR** (neat, cm^−1^) 3349 (OH), 1761 (C=O); **HRMS** (ES+) m/z: [M+H]^+^ calc. for C_22_H_30_NO_4_^+^: 372.2175, found: 372.2172; **mp**: 192–194 °C; **[α]**_**D**_^**20**^ = −6.58° (c 4, CHCl_3_).

#### 4.2.16 Synthesis of **6** 3-((Phenylamino)methyl)dihydrofuran-2(3H)-one

Following general procedure A, a mixture of tulipane (100 mg, 1.00 mmol), aniline (112 mg, 1.20 mmol) and squaric acid (11 mg, 0.10 mmol), was stirred at 50 °C in 1:1 water-methanol (20 mL) for 48 h. The title compound was obtained as a colourless solid in 95% yield (181 mg, 0.948 mmol). **Rf** = 0.68 (dichloromethane/methanol, 95%:5%); ^**1**^**H NMR** (400 MHz, CDCl_3_): δ 7.22–7.14 (2H, m, ArH), 6.73 (1H, tt, *J* 7.3 & 1.0, ArH), 6.68–6.62 (2H, m, ArH), 4.40–4.32 (1H, td, *J* 8.8 & 2.6, CH_2_), 4.26 (1H, br s, NH), 4.24–4.15 (1H, td, *J* 9.8 & 6.7, CH_2_), 3.52–3.37 (2H, m, CH_2_), 2.92–2.82 (1H, m, CH), 2.45–2.31 (1H, m, CH_2_), 2.15–2.05 (1H, m, CH_2_); ^**13**^**C NMR** (101 MHz, CDCl_3_): δ 178.5 (C=O), 147.6 (C–N), 129.4 (CH), 118.1 (CH), 113.2 (CH), 66.8 (CH_2_), 43.9 (CH_2_), 39.2 (CH), 26.8 (CH_2_); **IR** (neat, cm^−1^) 3345 (OH), 1765 (C=O); **HRMS** (ES+) m/z: [M+Na]^+^ calc. for C_11_H_13_NO_2_Na^+^: 214.0844, found: 214.0837; **mp**: 60–62 °C.

##### 4.2.16.1 Synthesis of **7** (3aS,6R,6aR,9R,9aS,9bS)-9-hydroxy-6-methoxy-6,9-dimethyl-3-methylenedecahydroazuleno[4,5-b]furan-2(3H)-one

To a solution of parthenolide (100 mg, 0.403 mmol) in 10 mL of methanol was added squaric acid (5 mg, 0.04 mmol) and the resulting mixture was stirred at room temperature for three days. After completion of the reaction, the solvent was removed *in vacuo*, the residual was extracted with ethyl acetate (3 × 25 mL), the combined organic layers were washed with brine and then dried over anhydrous magnesium sulphate, filtered, and solvent removed *in vacuo*. The product was isolated by flash chromatography over silica gel. The title compound was obtained as a colourless solid in 40% yield (45 mg, 0.16 mmol). **Rf** = 0.46 hexane/ethyl acetate, 50%:50%); ^**1**^**H NMR** (400 MHz, CDCl_3_): δ 6.22 (1H, d, *J* 3.5, CH_2_), 5.51 (1H, d, *J* 3.3, CH_2_), 4.21 (1H, dd, *J* 11.7 & 9.9, CH), 3.19 (3H, s, Me), 2.90–2.72 (2H, m), 2.34 (1H, t, *J* 11.7, CH), 2.33 (1H, br s, OH), 2.21–2.12 (1H, m, CH_2_), 2.01–1.93 (1H, m, CH_2_), 1.90–1.77 (3H, m), 1.75–1.66 (1H, m, CH_2_), 1.60–1.50 (1H, m, CH_2_), 1.48–1.37 (1H, m, CH_2_), 1.39 (3H, s, Me), 1.16 (3H, s, Me); ^**13**^**C NMR** (101 MHz, CDCl_3_): δ 169.6 (C=O), 39.0 (Cq), 120.1 (CH_2_), 82.7 (CH), 80.5 (Cq), 78.1 (Cq), 55.5 (CH), 48.3 (CH_3_), 46.8 (CH), 46.0 (CH), 39.0 (CH_2_), 36.0 (CH_2_), 25.5 (CH_2_), 24.5 (CH_2_), 24.3 (CH_3_), 22.6 (CH_3_); **IR** (neat, cm^−1^) 3470 (OH), 1751 (C=O); **HRMS** (ES+) m/z: [M + Na]^+^ calc. for C_16_H_24_O_4_Na^+^: 303.1572, found: 303.1570; **mp**: 105–107 °C; **[α]**_**D**_^**23**^ = −2.77° (c = 0.5, CH_2_Cl_2_).

#### 4.2.17 Synthesis of **8** (3R,3aS,6R,6aR,9R,9aS,9bS)-3-((Dimethylamino)methyl)-9-hydroxy-6-methoxy-6,9-dimethyldecahydroazuleno[4,5-b]furan-2(3H)-one

A mixture of parthenolide analogue **8** (34 mg, 0.12 mmol), dimethylamine (11 mg, 0.24 mmol) and potassium carbonate (33 mg, 0.24 mmol), was stirred at room temperature in ethanol (10 mL) for 16 h. The solvent was removed in vacuo and the product was isolated by flash chromatography over silica gel as a colourless solid in 89% yield (34 mg, 0.11 mmol). **Rf** = 0.37 (hexane/ethyl acetate, 50%:50%); ^**1**^**H NMR** (400 MHz, CDCl_3_): δ 4.27 (1H, dd, *J* 11.6 & 10.2, CH), 3.17 (3H, s, Me), 2.84–2.74 (1H, m, CH), 2.70 (1H, dd, *J* 12.9 & 5.0, CH_2_), 2.55 (1H, dd, *J* 12.9 & 6.6, CH_2_), 2.46 (1H, br s, OH), 2.40–2.32 (1H, m, CH), 2.32–2.18 (3H, m), 2.24 (6H, s, Me), 2.11–1.99 (1H, m, CH), 1.95–1.77 (3H, m), 1.66 (1H, td, *J* 13.4 & 3.7, CH_2_), 1.60–1.50 (1H, m, CH_2_), 1.42–1.31 (1H, m, CH_2_), 1.34 (3H, s, Me), 1.16 (3H, s, Me); ^**13**^**C NMR** (101 MHz, CDCl_3_): δ 176.9 (C=O), 82.9 (CH), 80.2 (Cq), 78.0 (Cq), 58.4 (CH_2_), 54.9 (CH), 48.5 (CH), 48.1 (CH_3_), 46.0 (CH_3_), 45.6 (CH), 44.7 (CH), 39.3 (CH_2_), 37.8 (CH_2_), 26.0 (CH_2_), 25.5 (CH_2_), 23.7 (CH_3_), 22.0 (CH_3_); **IR** (neat, cm^−1^): 3477 (OH), 1762 (C=O), **HRMS** (ES+) m/z: [M + H]^+^ calc. for C_18_H_32_NO_4_^+^: 326.2331, found: 326.2323; **mp**: 138–140 °C; **[α]**_**D**_^**20**^ = 7.20 (c = 1.5, CHCl_3_).

## Declaration of competing interest

The authors declare that they have no known competing financial interests or personal relationships that could have appeared to influence the work reported in this paper.

## Appendix Supplementary data

Supplementary Material Supplementary Material

cif check **3d** cif check 3d

cif check **7** cif check 7

cif check **3b** cif check 3b

3d cif file

7 cif file

3b cif file
