# Supplementary material for: Aniline-containing derivatives of parthenolide: Synthesis and anti-chronic lymphocytic leukaemia activity
Source: Tetrahedron. 2020 Nov 27;76(48):131631. doi: 10.1016/j.tet.2020.131631 (PMC7695678; doi:10.1016/j.tet.2020.131631)
Supplement: Supplementary Material [file mmc1.pdf]

Supplementary Material

Aniline-containing derivatives of parthenolide: Synthesis and anti-chronic lymphocytic leukaemia activity

Alex S. Quy,[a] Xingjian Li,[a] Louise Male,[b] Tatjana Stankovic,[c] Angelo Agathanggelou [c] and John S. Fossey [a]

[a] School of Chemistry, University of Birmingham, Edgbaston, Birmingham, West Midlands, B15 2TT, U.K.
[b] X-Ray Crystallography Facility, School of Chemistry, University of Birmingham, Edgbaston, Birmingham, West Midlands, B15 2TT, U.K.
[c] Institute for Cancer and Genomic Sciences, University of Birmingham, Edgbaston, Birmingham, West Midlands, U.K.

Table of contents 1
General procedures 1
Synthetic chemistry 1
Biological assays 2
General procedure A: 3
General procedure B: 3
Chemical synthesis 4
Synthesis of 3a 4
Synthesis of 3b 4
Synthesis of 3c 5
Synthesis of 3d 5
Synthesis of 3e 6
Synthesis of 3f 6
Synthesis of 3g 7
Synthesis of 3h 7
Synthesis of 3i 8
Synthesis of 3j 8
Synthesis of 3k 9
Synthesis of 3l 9
Synthesis of 3m 10
Synthesis of 3n 10
Synthesis of 3o 11
Synthesis of 6 11
Synthesis of 7 12
Synthesis of 8 13
X-Ray Crystallography 14
XRD compound 3b 14
XRD compound 3d 15
XRD compound 7 16
NMR Spectrums 17
Supplementary References 55

General procedures

Synthetic chemistry

All commercially available solvents, catalysts and reagents were purchased and used from suppliers without any further purification. Proton NMR spectra were recorded at 400 MHz on a Bruker

AVIII400 NMR spectrometer. Carbon NMR spectra are proton decoupled and were recorded at 101 MHz on a Bruker AVIII400 NMR spectrometer at room temperature. Fluorine NMR spectra are proton decoupled and were recorded at 377 MHz on a Bruker AVIII400NMR spectrometer at room temperature. Chemical shifts (δ) were reported in ppm relative to TMS (δ 0.00) for ^{1}H NMR and to chloroform (δ 77.16) for ^{13}C NMR spectroscopy; coupling constants (*J*) are expressed in Hertz (Hz). The following abbreviations are used for multiplicities: s = singlet, d = doublet, t = triplet, q = quartet, quint = quintet, m = multiplet, pent = pentet, hex = hextet, and br = broad. Mass spectra were recorded on an electrospray MS Waters LCT Time of Flight Mass Spectrometer and with EI (GC/MS) Waters GCT Premier Time of Flight Mass Spectrometer. Infrared Spectra Varian 660-IR FT-IR spectrometer at room temperature using an ATR attachment. Melting points were measured using a StuartTM digital melting point apparatus (SMP10) and reported as a range. Specific optical rotations were recorded on an Optical PolAAr 2001 automatic polarimeter at room temperature. The X-ray crystal structures were determined using an Agilent SuperNova X-ray diffractometer with an Atlas detector (wavelength 1.5418 Å). Column chromatography was carried out using standard flash column chromatography and a Combiflash Rf 200i (stationary phase silica), chromatograms were recorded by evaporative light scattering detector (ELSD) and absorbance at two wavelengths (254 nm and 280 nm). Reactions were monitored by thin layer chromatography (TLC) on Merck silica gel 60 F254 plates. TLC plates were visualised by either UV light with 254 nm / 365 nm, a methanolic solution of ninhydrin or with potassium permanganate.

Biological assays

Tissue culture, MEC1 cells were obtained from the American Type Culture Collection (Manassas, VA 20110 USA) and were cultured in RPMI 1640 medium (Sigma-Aldrich, Irvine, UK) with 10% fetal bovine serum (Sigma-Aldrich). The alamarBlue® cytotoxicity assay was conducted by seeding MEC1 cells in triplicate at density of 25000 cells/well in a 96 well plate, final volume of 200 µL.[1] Following treatment with test compound, viability was determined by measuring the reduction of resazurin. Resazurin solution was added to each well at a final concentration of 50 µg/mL and incubated for 3 hours at 37 °C with 5% CO_2. Reduction of resazurin was determined by measuring absorbance at 590 nm using a PheraSTAR FS plate reader (BMG Labtech). Cell viability was calculated as a fraction of the untreated cells after subtracting background fluorescence of resazurin in media only. Data is presented as the mean of five independent experiments and significance was determined by Student's t-test.

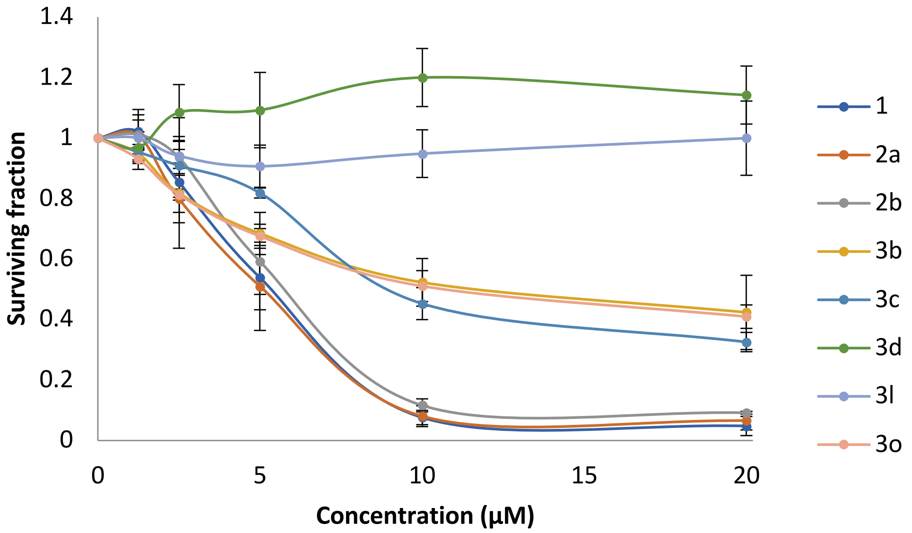

***Supplementary Figure 1**. Concentration* versus *activity plots showing the anti-leukaemic activity of* N-*aryl derivatives (**3**) derivatives versus parthenolide (**1**) and amine derivatives **2a** and **2b**.*

General procedure A:

Synthesis of aryl aminoparthenolide derivatives

To a 1:1 water-methanol (0.2 M) solution of α,β-unsaturated ester (1 equiv.), the corresponding aniline (1.2 equiv.) and squaric acid (10 mol%.) were added. The resulting solution was stirred at 50 °C for 48 hours. The solution was allowed to cool to room temperature and the solvent was removed *in vacuo*. The residual was extracted with dichloromethane (3 × 25 mL), the combined organic layers were washed with brine and then dried over anhydrous magnesium sulphate, filtered, and solvent removed *in vacuo*. The product was isolated by flash chromatography over silica gel.

General procedure B:

Synthesis of tertiary aniline parthenolide derivatives from secondary aniline parthenolide derivatives

To a solution of aniline secondary derivative (1 equiv.) in 1,2-dichloroethene (10 mL), formaldehyde (2 equiv.) and sodium triacetoxyborohydride (3 equiv.) were added. The reaction mixture was stirred under nitrogen protection at room temperature for 48 hours and quenched with saturated aqueous sodium bicarbonate. The solvent was removed *in vacuo* and the residual was extracted with ethyl acetate. The combined organic solution was dried over anhydrous magnesium sulphate, filtered and concentrated *in vacuo*. Further purification was carried out by flash chromatography over silica gel.

Chemical synthesis

Synthesis of 3a

(3R,3aS,9aR,10aR,10bS,E)-6,9a-Dimethyl-3-((phenylamino)methyl)-3a,4,5,8,9,9a,10a,10b-octahydrooxireno[2',3':9,10]cyclodeca[1,2-b]furan-2(3H)-one

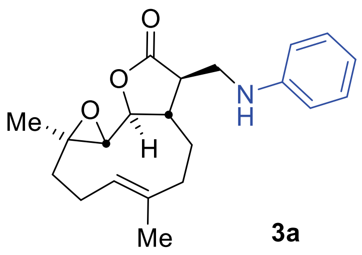

3a

Following the general procedure A, a mixture of parthenolide (0.150 g, 0.60 mmol), aniline (0.075 g, 0.80 mmol) and squaric acid (0.007 g, 0.06 mmol), was stirred at 50 °C in 1:1 water-methanol (20 mL) for 48 hours. The title compound was obtained as a pale-yellow oil in 72% yield (0.148 g, 0.43 mmol). **Rf** = 0.49 (hexane/ethyl acetate, 50%:50%); **^{1}H NMR** (400 MHz, $CDCl_3$): δ 7.21-7.17 (2H, m, ArH), 6.74 (1H, t, *J* 7.3, ArH), 6.65 (2H, dd, *J* 8.6 & 0.9, ArH), 5.12 (1H, dd, *J* 12.0 & 2.2, CH), 4.44 (1H, br s, NH), 3.84 (1H, t, *J* 9.0, CH), 3.58 (1H, dd, *J* 13.8 & 3.9, CH_2), 3.39 (1H, dd, *J* 13.8 & 6.7, CH_2), 2.68 (1H, d, *J* 9.1, CH, epoxide moiety), 2.60-2.52 (1H, m, CH), 2.43-2.26 (2H, m), 2.18-2.08 (3H, m), 2.07-1.93 (2H, m), 1.76-1.66 (1H, m, CH_2), 1.68 (3H, s, Me), 1.27 (3H, s, Me), 1.20 (1H, td, *J* 15.9 & 3.0 CH_2); **^{13}C NMR** (101 MHz, $CDCl_3$): δ 176.5 (C=O), 147.8 (Cq), 134.3 (Cq), 129.4 (CH), 125.3 (CH), 118.2 (CH), 113.3 (CH), 82.6 (CH), 66.2 (CH, epoxide moiety), 61.6 (Cq), 47.5 (CH), 46.8 (CH), 42.0 (CH_2), 41.0 (CH_2), 36.6 (CH_2), 30.1 (CH_2), 24.1 (CH_2), 17.2 (CH_3), 16.9 (CH_3); **IR** (neat, cm^{-1}) 1761 (C=O); **HRMS** (ES+) m/z: $[M+Na]^+$ calc. for $C_{21}H_{27}NO_3Na^+$: 364.1889, found: 364.1887; **$[\alpha]_D^{20}$** = -0.26 (c 10.0, $CHCl_3$).

Synthesis of 3b

(3R,3aS,9aR,10aR,10bS,E)-3-(((4-Hydroxyphenyl)amino)methyl)-6,9a-dimethyl-3a,4,5,8,9,9a,10a,10b-octahydrooxireno[2',3':9,10]cyclodeca[1,2-b]furan-2(3H)-one (3b)

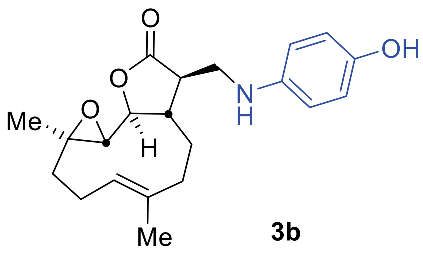

3b

Following the general procedure A, a mixture of parthenolide (0.200 g, 0.81 mmol), 4-aminophenol (0.119 g, 1.06 mmol) and squaric acid (0.009 g, 0.08 mmol), was stirred at 50 °C in 1:1 water-methanol (20 mL) for 48 hours. The title compound was obtained as a colourless solid in 77% yield (0.223 g, 0.62 mmol). **Rf** = 0.46 (hexane/ethyl acetate, 50%:50%); **^{1}H NMR** (400 MHz, $CDCl_3$): δ 6.71 (2H, d, *J* 8.7, ArH), 6.56 (2H, d, *J* 8.7, ArH), 5.15 (1H, dd, *J* 11.8 & 2.0, CH), 3.83 (1H, t, *J* 9.0, CH), 3.48 (1H, dd, *J* 13.6 & 3.8, CH_2), 3.29 (1H, dd, *J* 13.5 & 7.2, CH_2), 2.70 (1H, d, *J* 9.0, CH, epoxide moiety), 2.59-2.50 (1H, m, CH), 2.44-2.24 (2H, m), 2.19-2.07 (3H, m), 2.04-1.88 (2H, m), 1.74-1.61 (1H, m, CH_2), 1.67 (3H, s, Me), 1.27 (3H, s, Me), 1.18 (1H, td, *J* 12.8 & 5.8, CH_2); **^{13}C NMR** (101 MHz, $CDCl_3$): δ 176.9 (C=O), 148.9

(Cq), 141.2 (Cq), 134.4 (Cq), 125.2 (CH), 116.4 (CH), 115.5 (CH), 82.6 (CH), 66.3 (CH, epoxide), 61.9 (Cq), 47.0 (CH), 46.9 (CH), 43.6 (CH_2), 41.0 (CH_2), 36.5 (CH_2), 30.1 (CH_2), 24.1 (CH_2), 17.2 (CH_3), 16.9 (CH_3); **IR** (neat, cm^{-1}) 3345 (OH), 1758 (C=O); **HRMS** (ES+) m/z: $[M+Na]^+$ calc. for $C_{21}H_{27}NO_4Na^+$: 380.1838, found: 380.1831; **mp**: 85-87 °C; **$[\alpha]_D^{20}$** = -7.42° (c 7.0, $CHCl_3$).

Synthesis of 3c

(3R,3aS,9aR,10aR,10bS,E)-3-(((2-Hydroxyphenyl)amino)methyl)-6,9a-dimethyl-3a,4,5,8,9,9a,10a,10b-octahydrooxireno[2',3':9,10]cyclodeca[1,2-b]furan-2(3H)-one

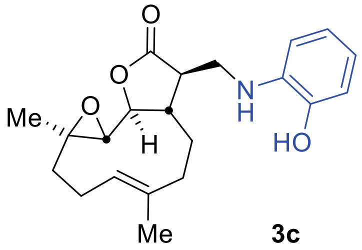

3c

Following the general procedure A, a mixture of parthenolide (0.100 g, 0.40 mmol), 2-aminophenol (0.088 g, 0.40 mmol) and squaric acid (0.010 g, 0.04 mmol), was stirred at 50 °C in 1:1 water-methanol (20 mL) for 48 hours. The title compound was obtained as a colourless solid in 75% yield (0.107 g, 0.30 mmol). **Rf** = 0.40 (hexane/ethyl acetate, 50%:50%); **^{1}H NMR** (400 MHz, $CDCl_3$): δ 6.88 (2H, m, ArH), 6.75 (2H, m, ArH), 5.10 (1H, dd, *J* 11.8 & 1.8, CH), 3.85 (1H, t, *J* 9.0, CH), 3.56 (1H, dd, *J* 13.7 & 4.0, CH_2), 3.44 (1H, dd, *J* 13.7 & 6.2, CH_2), 2.71 (1H, d, *J* 8.9, CH, epoxide moiety), 2.65-2.56 (1H, m, CH), 2.43-2.22 (2H, m), 2.20-2.08 (3H, m), 2.05-1.90 (2H, m), 1.72-1.64 (1H, m, CH_2), 1.68 (3H, s, Me), 1.28 (3H, s, Me), 1.20 (1H, td, *J* 12.8 & 5.9, CH_2); **^{13}C NMR** (101 MHz, $CDCl_3$): δ 176.7 (C=O), 144.8 (Cq), 136.4 (Cq), 134.4 (Cq), 125.2 (CH), 121.3 (CH), 119.1 (CH), 115.0 (CH), 113.2 (CH), 82.6 (CH), 66.3 (CH, epoxide moiety), 61.8 (Cq), 47.7 (CH), 46.9 (CH), 42.7 (CH_2), 41.0 (CH_2), 36.5 (CH_2), 30.1 (CH_2), 24.1 (CH_2), 17.2 (CH_3), 16.9 (CH_3); **IR** (neat, cm^{-1}) 3386 (OH), 1764 (C=O); **HRMS** (ES+) m/z: [M+H]+ calc. for $C_{21}H_{28}NO_4^+$: 358.2018, found: 358.2016; **mp**: 195-198 °C; **$[\alpha]_D^{20}$** = -1.08° (c 2.3, $CHCl_3$).

Synthesis of 3d

(3R,3aS,9aR,10aR,10bS,E)-6,9a-Dimethyl-3-((p-tolylamino)methyl)-3a,4,5,8,9,9a,10a,10b-octahydrooxireno[2',3':9,10]cyclodeca[1,2-b]furan-2(3H)-one

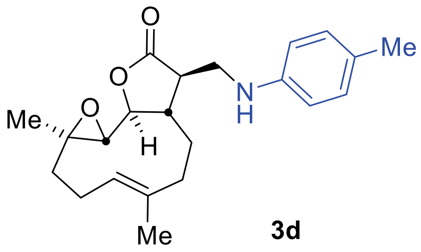

3d

Following the general procedure A, a mixture of parthenolide (100 mg, 0.403 mmol), p-toluidine (52 mg, 0.483 mmol) and squaric acid (5 mg, 0.04 mmol) was stirred at 50 °C in 1:1 water-methanol (2 mL) for 48 hours. The title compound was obtained as a white solid in 51% yield (73 mg). **Rf** = 0.65 (hexane/ethyl acetate, 50%:50%); **^{1}H NMR** (400 MHz, $CDCl_3$) δ 7.01 (2H, d, *J* 8.2), 6.58 (2H, d, *J* 8.4), 5.13 (1H, d, *J* 8.9), 4.28 (1H, s), 3.84 (1H, t, *J* 9.1), 3.56 (1H, d, *J* 13.8), 3.36 (1H, dd, *J* 13.7 & 6.8), 2.69 (1H, d, *J* 8.9), 2.60-2.51 (1H, m), 2.43-2.27 (2H, m), 2.24 (3H, s), 2.20-1.93 (5H, m), 1.76-1.60 (1H, m), 1.69 (3H, s), 1.28 (3H, s), 1.19 (1H, td,

J 13.4 & 5.8); **^{13}C NMR** (101 MHz, $CDCl_3$) δ 176.5, 145.5, 134.3, 129.9, 127.6, 125.3, 113.6, 82.5, 66.2, 61.6, 47.4, 46.8, 42.5, 41.1, 36.6, 30.1, 24.1, 20.4, 17.2, 16.9; **IR** (neat, cm^{-1}) 3363 (NH), 1747 (C=O); **HRMS** (TOF MS ES+) *m/z*: $[M+H]^+$ calc. for $C_{22}H_{30}NO_3^+$ 356.2224, found 356.2226; **mp** 200-203 °C; **$[\alpha]_D^{22}$** = -12.81° (c 2.0, CH_2Cl_2).

Synthesis of 3e

(3R,3aS,9aR,10aR,10bS,E)-3-(((4-Fluorophenyl)amino)methyl)-6,9a-dimethyl-3a,4,5,8,9,9a,10a,10b-octahydrooxireno[2',3':9,10]cyclodeca[1,2-b]furan-2(3H)-one

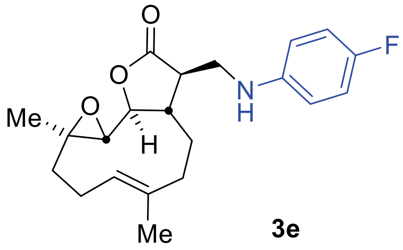

3e

Following the general procedure A, a mixture of parthenolide (100 mg, 0.403 mmol), 4-fluoroaniline (54 mg, 0.483 mmol) and squaric acid (5 mg, 0.04 mmol) was stirred at 50 °C in 1:1 water-methanol (2 mL) for 48 hours. The title compound was obtained as a white solid in 35% yield (51 mg). **Rf** = 0.57 (hexane/ethyl acetate, 50%:50%); **^{1}H NMR** (400 MHz, $CDCl_3$) δ 6.97-6.86 (2H, m), 6.66-6.56 (2H, m), 5.15 (1H, d, *J* 12.3), 4.37 (1H, s), 3.87 (1H, t, *J* 9.0), 3.54 (1H, dd, *J* 13.5 & 3.8), 3.34 (1H, dd, *J* 13.5 & 7.1), 2.70 (1H, d, *J* 8.9), 2.56 (1H, ddd, *J* 12.4, 7.1 & 3.8), 2.47-2.28 (2H, m), 2.22-1.91 (5H, m), 1.81-1.67 (1H, m), 1.70 (3H, s), 1.29 (3H, s), 1.27-1.14 (1H, m); **^{13}C NMR** (101 MHz, $CDCl_3$) δ 176.4, 157.5, 144.0, 134.2, 125.4, 116.0, 115.8, 114.5, 114.4, 82.6, 66.2, 61.6, 47.3, 46.9, 43.0, 41.1, 36.5, 30.1, 24.1, 17.2, 16.9; **^{19}F-NMR** (377 MHz, $CDCl_3$) δ -126.83; **IR** (neat, cm^{-1}) 3371 (NH), 1748 (C=O); **HRMS** (TOF MS ES+) *m/z*: $[M+H]^+$ calc. for $C_{21}H_{27}NO_3^+$ 360.1974, found 360.1975; **mp** 121-124 °C; **$[\alpha]_D^{22}$** = -19.39° (c 0.5, CH_2Cl_2).

Synthesis of 3f

(3R,3aS,9aR,10aR,10bS,E)-3-(((4-Bromophenyl)amino)methyl)-6,9a-dimethyl-3a,4,5,8,9,9a,10a,10b-octahydrooxireno[2',3':9,10]cyclodeca[1,2-b]furan-2(3H)-one

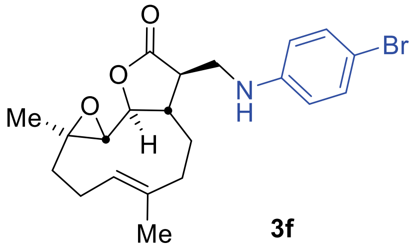

3f

Following the general procedure A, a mixture of parthenolide (100 mg, 0.403 mmol), 4-bromoaniline (83 mg, 0.483 mmol) and squaric acid (5 mg, 0.04 mmol) was stirred at 50 °C in 1:1 water-methanol (2 mL) for 48 hours. The title compound was obtained as a white solid in 9% yield (15 mg). **Rf** = 0.57 (hexane/ethyl acetate, 50%:50%); **^{1}H NMR** (400 MHz, $CDCl_3$) δ 7.31-7.23 (2H, m), 6.58-6.49 (2H, m), 5.14 (1H, d, *J* 12.1), 4.48 (1H, t, *J* 6.8), 3.91-3.82 (1H, m), 3.59-3.52 (1H, m) 3.39-3.31 (1H, m), 2.69 (1H, d, *J* 8.9), 2.59-2.52 (1H, m), 2.45-2.28 (2H, m), 2.21-1.93 (5H, m), 1.79-1.70 (1H, m), 1.70 (3H, s), 1.29 (3H, s), 1.30-1.16 (1H, m); **^{13}C NMR** (101 MHz, $CDCl_3$) δ 176.3, 146.7, 134.2, 132.2, 125.5, 114.9, 109.9, 82.6, 66.2, 61.6, 60.4, 47.4, 46.9, 42.1, 41.1, 36.5, 30.1, 24.1, 17.2, 16.9, 14.2; **IR** (neat, cm^{-1}) 3380 (NH),

1760 (C=O); **HRMS** (TOF MS ASAP+) *m/z*: $[M+H]^+$ calcd. for $C_{21}H_{27}BrNO_3^+$ 420.1174 found 420.1179; **mp** 182-184 °C; $[\alpha]_D^{22}$ = 9.70° (c 0.5, CH_2Cl_2).

Synthesis of 3g

(3R,3aS,9aR,10aR,10bS,E)-3-(((4-Methoxyphenyl)amino)methyl)-6,9a-dimethyl-3a,4,5,8,9,9a,10a,10b-octahydrooxireno[2',3':9,10]cyclodeca[1,2-b]furan-2(3H)-one

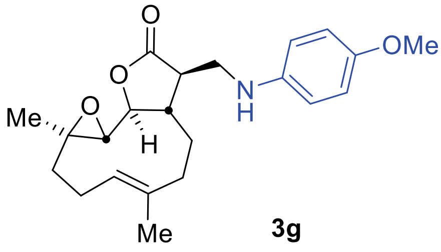

3g

Following the general procedure A, a mixture of parthenolide (100 mg, 0.403 mmol), 4-methoxyaniline (60 mg, 0.483 mmol) and squaric acid (5 mg, 0.04 mmol) was stirred at 50 °C in 1:1 water-methanol (2 mL) for 48 hours. The title compound was obtained as a white solid in 54% yield (81 mg). **Rf** = 0.50 (hexane/ethyl acetate, 50%:50%); **^{1}H NMR** (400 MHz, $CDCl_3$) δ 6.84-6.75 (2H, m), 6.68-6.60 (2H, m), 5.13 (1H, d, *J* 12.2), 4.21 (1H, s), 3.85 (1H, t, *J* 9.0), 3.75 (3H, s), 3.53 (1H, dd, *J* 13.5 & 3.8), 3.33 (1H, dd, *J* 13.5 & 7.0), 2.70 (1H, d, *J* 8.9), 2.56 (1H, ddd, *J* 12.3, 6.9 & 3.8), 2.45-2.26 (2H, m), 2.21-1.91 (5H, m), 1.78-1.65 (1H, m), 1.69 (3H, s), 1.28 (3H, s), 1.20 (1H, td, *J* 13.0 & 5.9); **^{13}C NMR** (101 MHz, $CDCl_3$) δ 176.6, 152.7, 141.8, 134.3, 125.3, 115.0, 82.6, 66.2, 61.6, 55.8, 47.2, 46.9, 43.4, 41.1, 36.6, 30.0, 24.1, 17.2, 16.9; **IR** (neat, cm^{-1}) 3365 (NH), 1747 (C=O); **HRMS** (TOF MS ES+) *m/z*: $[M+H]^+$ calcd. for $C_{22}H_{30}NO_4^+$ 372.2175, found 372.2174; **mp** 145-147 °C; $[\alpha]_D^{22}$ = -16.62° (c 1.0, CH_2Cl_2).

Synthesis of 3h

(3R,3aS,9aR,10aR,10bS,E)-3-(((3,5-Dimethoxyphenyl)amino)methyl)-6,9a-dimethyl-3a,4,5,8,9,9a,10a,10b-octahydrooxireno[2',3':9,10]cyclodeca[1,2-b]furan-2(3H)-one

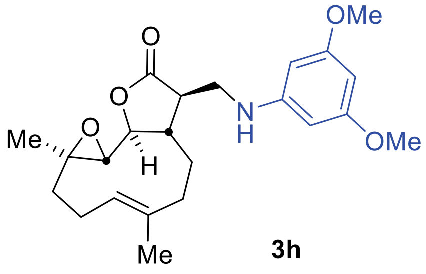

3h

Following the general procedure A, a mixture of parthenolide (100 mg, 0.403 mmol), 3,5-dimethoxyaniline (74 mg, 0.483 mmol) and squaric acid (5 mg, 0.04 mmol) was stirred at 50 °C in 1:1 water-methanol (2 mL) for 48 hours. The title compound was obtained as a white solid in 26% yield (42 mg). **Rf** = 0.50 (hexane/ethyl acetate, 50%:50%); **^{1}H NMR** (400 MHz, $CDCl_3$) δ 5.91 (1H, t, *J* 2.1), 5.83 (2H, d, *J* 2.1), 5.13 (1H, dd, *J* 11.9 & 2.3), 4.47 (1H, s), 3.86 (1H, t, *J* 9.0), 3.75 (6H, s), 3.56 (1H, dd, *J* 14.5 & 3.3), 3.36 (1H, dd, *J* 13.8 & 6.8), 2.69 (1H, d, *J* 8.9), 2.57 (1H, ddd, *J* 12.3, 6.8 & 3.7), 2.46-2.27 (2H, m), 2.21-1.94 (5H, m), 1.80-1.70 (1H, m), 1.69 (3H, s), 1.29 (3H, s), 1.20 (1H, td, *J* 13.4 & 6.3); **^{13}C NMR** (101 MHz, $CDCl_3$) δ 176.4, 161.9, 149.7, 134.3, 125.4, 92.1, 90.4, 82.6, 66.2, 61.6, 55.2, 47.5, 46.8, 41.8, 41.0, 36.6, 30.1, 24.1, 17.2, 16.9; **IR** (neat, cm^{-1}) 3389 (NH),

1761 (C=O); **HRMS** (TOF MS ES+) *m/z*: $[M+H]^+$ calcd. for $C_{23}H_{32}NO_5^+$ 402.2280, found 402.2285; **mp** 110-112 °C; $[\alpha]_D^{22}$ = -5.02° (c 2.0, CH_2Cl_2).

Synthesis of 3i

(3R,3aS,9aR,10aR,10bS,E)-3-(((3,5-Dimethylphenyl)amino)methyl)-6,9a-dimethyl-3a,4,5,8,9,9a,10a,10b-octahydrooxireno[2',3':9,10]cyclodeca[1,2-b]furan-2(3H)-one

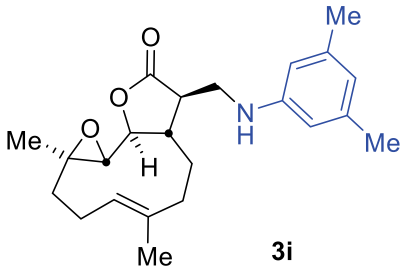

3i

Following the general procedure A, a mixture of parthenolide (100 mg, 0.403 mmol), 3,5-dimethylaniline (54 mg, 0.483 mmol) and squaric acid (5 mg, 0.04 mmol) was stirred at 50 °C in 1:1 water-methanol (2 mL) for 48 hours. The title compound was obtained as a white solid in 28% yield (42 mg). **Rf** = 0.62 (hexane/ethyl acetate, 50%:50%); ^{1}**H NMR** (400 MHz, $CDCl_3$) δ 6.41 (1H, s), 6.28 (2H, s), 5.12 (1H, dd, *J* 12.1 & 2.3), 4.27 (1H, s), 3.84 (1H, t, *J* 9.0), 3.58 (1H, dd, *J* 13.8 & 3.8), 3.39 (1H, dd, *J* 13.8 & 6.3), 2.68 (1H, d, *J* 8.9), 2.55 (1H, ddd, *J* 12.3, 6.3 & 3.8), 2.45-2.27 (2H, m), 2.24 (6H, s), 2.20-1.95 (5H, m), 1.79-1.66 (1H, m), 1.70 (3H, s), 1.29 (3H, s), 1.19 (1H, td, *J* 13.0 & 6.0); ^{13}**C NMR** (101 MHz, $CDCl_3$) δ 176.4, 147.9, 139.1, 134.3, 125.4, 120.2, 111.3, 82.5, 66.2, 61.6, 47.8, 46.7, 42.0, 41.0, 36.6, 30.1, 24.1, 21.5, 17.2, 16.9; **IR** (neat, cm^{-1}) 3382 (NH), 1755 (C=O); **HRMS** (TOF MS ES+) *m/z*: $[M+H]^+$ calcd. for $C_{23}H_{32}NO_3^+$ 370.2383, found 370.2382; **mp** 196-197 °C; $[\alpha]_D^{22}$ = 11.08° (c 0.5, CH_2Cl_2).

Synthesis of 3j

(3R,3aS,9aR,10aR,10bS,E)-3-(((3-Methoxyphenyl)amino)methyl)-6,9a-dimethyl-3a,4,5,8,9,9a,10a,10b-octahydrooxireno[2',3':9,10]cyclodeca[1,2-b]furan-2(3H)-one

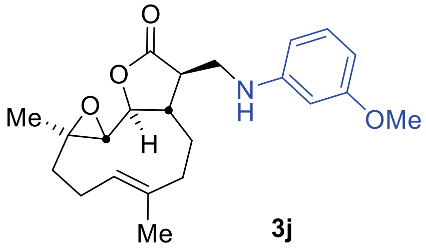

3j

Following the general procedure A, a mixture of parthenolide (80 mg, 0.32 mmol), 3-methoxyaniline (48 mg, 0.384 mmol) and squaric acid (4 mg, 0.03 mmol) was stirred at 50 °C in 1:1 water-methanol (2 mL) for 48 hours. The title compound was obtained as a white solid in 24% yield (28 mg). **Rf** = 0.52 (hexane/ethyl acetate, 50%:50%); ^{1}**H NMR** (400 MHz, $CDCl_3$) δ 7.10 (1H, t, *J* 8.1), 6.31 (1H, dd, *J* 8.2 & 2.4), 6.26 (1H, dd, *J* 8.0 & 2.2), 6.20 (1H, t, *J* 2.3), 5.13 (1H, d, *J* 12.2 & 2.4), 4.43 (1H, s), 3.85 (1H, t, *J* 9.0), 3.77 (3H, s), 3.58 (1H, dd, *J* 14.1 & 3.6), 3.39 (1H, dd, *J* 13.8 & 6.7), 2.69 (1H, d, *J* 8.9), 2.56 (1H, ddd, *J* 12.3, 6.7 & 3.8), 2.46-2.25 (2H, m), 2.20-1.94 (5H, m), 1.79-1.66 (1H, m), 1.69 (3H, s), 1.29 (3H, s), 1.27-1.14 (1H, m); ^{13}**C NMR** (101 MHz, $CDCl_3$) δ 176.4, 161.0, 149.2, 134.3, 130.2, 125.4, 106.3, 103.4, 99.4, 82.6, 66.2, 61.6, 55.2, 47.6, 46.8, 41.9, 41.0, 36.6, 30.1, 24.1, 17.2, 16.9; **IR** (neat, cm^{-1}) 2930

(NH), 1762 (C=O); **HRMS** (TOF MS ES+) *m/z*: $[M+H]^+$ calcd. for $C_{22}H_{30}NO_4^+$ 372.2175, found 372.2177; **mp** 154-156 °C; $[\alpha]_D^{22}$ = -17.31° (c 1.0, CH_2Cl_2).

Synthesis of 3k

(3R,3aS,9aR,10aR,10bS,E)-6,9a-Dimethyl-3-((m-tolylamino)methyl)-3a,4,5,8,9,9a,10a,10b-octahydrooxireno[2',3':9,10]cyclodeca[1,2-b]furan-2(3H)-one

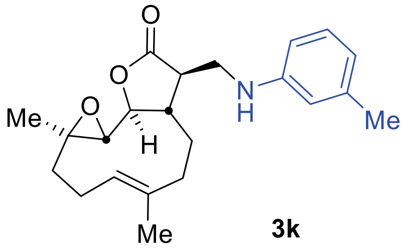

3k

Following the general procedure A, a mixture of parthenolide (80 mg, 0.32 mmol), m-toluidine (42 mg, 0.384 mmol) and squaric acid (4 mg, 0.03 mmol) was stirred at 50 °C in 1:1 water-methanol (2 mL) for 48 hours. The title compound was obtained as a white solid in 18% yield (20 mg). **Rf** = 0.64 (hexane/ethyl acetate, 50%:50%); **^{1}H NMR** (400 MHz, $CDCl_3$) δ 7.08 (1H, t, *J* 7.5), 6.57 (1H, d, *J* 7.2), 6.50-6.43 (2H, m), 5.12 (1H, dd, *J* 12.2 & 3.8), 4.35 (1H, s), 3.85 (1H, t, *J* 9.0), 3.58 (1H, dd, *J* 13.8 & 3.9), 3.40 (1H, dd, *J* 13.8 & 6.6), 2.68 (1H, d, *J* 8.9), 2.56 (1H, ddd, *J* 12.2, 6.5 & 3.8), 2.45-2.30 (2H, m), 2.28 (3H, s), 2.20-1.95 (5H, m), 1.79-1.66 (1H, m), 1.69 (3H, s), 1.29 (3H, s), 1.19 (1H, td, *J* 13.0 & 5.9); **^{13}C NMR** (101 MHz, $CDCl_3$) δ 176.4, 147.8, 139.3, 134.3, 129.3, 125.4, 119.2, 114.2, 110.4, 82.6, 66.2, 61.6, 47.6, 46.8, 42.0, 41.0, 36.6, 30.1, 24.1, 21.6, 17.2, 16.9; **IR** (neat, cm^{-1}) 3385 (NH), 1761 (C=O); **HRMS** (TOF MS ES+) *m/z*: $[M+H]^+$ calc. for $C_{22}H_{30}NO_3^+$ 356.2224, found 356.2230; **mp** 154-156 °C; $[\alpha]_D^{22}$ = -7.62° (c 0.5, CH_2Cl_2).

Synthesis of 3l

(3R,3aS,9aR,10aR,10bS,E)-6,9a-Dimethyl-3-((methyl(phenyl)amino)methyl)-3a,4,5,8,9,9a,10a,10b-octahydrooxireno[2',3':9,10]cyclodeca[1,2-b]furan-2(3H)-one

Method (i)

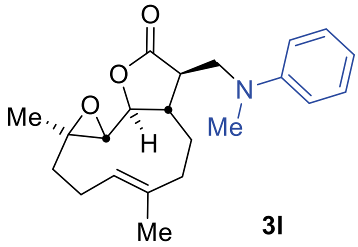

3l

Following the general procedure A, a mixture of parthenolide (100 mg, 0.403 mmol), *N*-methylaniline (52 mg, 0.483 mmol) and squaric acid (5 mg, 0.04 mmol) was stirred at 50 °C in 1:1 water-methanol (2 mL) for 48 hours. The title compound was obtained as a white solid in 8% yield (9 mg). **Rf** = 0.67 (hexane/ethyl acetate, 50%:50%); **^{1}H NMR** (400 MHz, $CDCl_3$) δ 7.29-7.22 (2H, m), 6.79-6.70 (3H, m), 5.05 (1H, dd, *J* 11.8 & 2.3), 4.01 (1H, dd, *J* 15.4 & 4.9), 3.79 (1H, t, *J* 9.0), 3.65 (1H, dd, *J* 15.5 & 6.1), 3.01 (3H, s), 2.74-2.62 (2H, m), 2.41-2.28 (1H, m), 2.19-2.03 (4H, m), 1.96-1.86 (1H, m), 1.79-1.68 (1H, m), 1.64 (3H, s), 1.65-1.53 (1H, m), 1.26 (3H, s), 1.19 (1H, td, *J* 13.0 & 5.9); **^{13}C NMR** (101 MHz, $CDCl_3$) δ 176.0, 149.0, 134.4, 129.5, 125.0, 117.2, 112.5, 82.3, 66.5, 61.6, 52.1, 48.1, 46.4, 40.9, 39.6, 36.6, 30.4, 24.1, 17.2, 16.9; **IR** (neat, cm^{-1}) 1760

(C=O); **HRMS** (TOF MS ES+) *m/z*: $[M+H]^+$ calc. for $C_{22}H_{30}NO_3^+$ 356.2224, found 356.2232; **mp** 153-156 °C; $[\alpha]_D^{22}$ = -124.65° (c 1.0, CH_2Cl_2).

Method (ii)

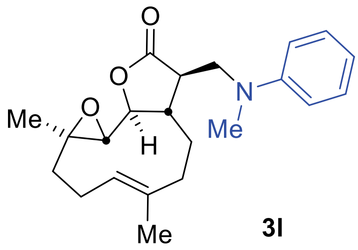

3l

Following the general procedure B, a mixture of aminoparthenolide derivative **3a** (0.109 g, 0.32 mmol), formaldehyde (0.030 g, 0.96 mmol) and sodium triacetoxyborohydride (0.112 g, 0.45 mmol), was stirred at room temperature in dichloroethane (10 mL) for 16 hours. The title compound was obtained as a colourless solid in 61% yield (0.072 g, 0.20 mmol). ^{1}H NMR, ^{13}C NMR, melting point and HRMS all consistent as above.

Synthesis of 3m

(3R,3aS,9aR,10aR,10bS,E)-3-(((4-Fluorophenyl)(methyl)amino)methyl)-6,9a-dimethyl-3a,4,5,8,9,9a,10a,10b-octahydrooxireno[2',3':9,10]cyclodeca[1,2-b]furan-2(3H)-one

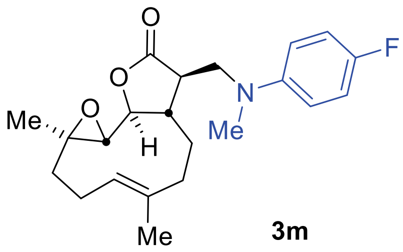

3m

Following the general procedure A, a mixture of parthenolide (80 mg, 0.32 mmol), 4-fluoro-*N*-methylaniline (48 mg, 0.384 mmol) and squaric acid (4 mg, 0.03 mmol) was stirred at 50 °C in 1:1 water-methanol (2 mL) for 48 hours. The title compound was obtained as a white solid in 8% yield (9 mg). **Rf** = 0.64 (hexane/ethyl acetate, 50%:50%); ^{1}**H NMR** (400 MHz, $CDCl_3$) δ 7.02-6.91 (2H, m), 6.75-6.65 (2H, m), 5.07 (1H, dd, *J* 12.2 & 2.0), 3.92 (1H, dd, *J* 15.4 & 4.9), 3.80 (1H, t, *J* 9.0), 3.60 (1H, dd, *J* 15.4 & 6.0), 2.97 (3H, s), 2.67 (1H, d, *J* 8.9), 2.63 (1H, ddd, *J* 11.9, 6.0 & 4.9), 2.44-2.28 (1H, m), 2.19-2.06 (4H, m), 1.89 (1H, dd, *J* 15.2 & 6.6), 1.76 (1H, t, *J* 13.0), 1.65 (3H, s), 1.66-1.54 (1H, m), 1.27-1.13 (4H, m); ^{13}**C NMR** (101 MHz, $CDCl_3$) δ 176.0, 145.8, 134.3, 125.1, 115.9, 115.7, 113.9, 113.9, 82.3, 66.5, 61.6, 52.8, 48.1, 46.4, 41.0, 40.1, 36.6, 30.3, 24.1, 17.2, 16.9; ^{19}**F-NMR** (377 MHz, $CDCl_3$) δ -128.2; **IR** (neat, cm^{-1}) 1761 (C=O); **HRMS** (TOF MS ASAP+) *m/z*: $[M+H]^+$ calc. for $C_{22}H_{29}FNO_3^+$ 374.2131, found 374.2141; **mp** 131-134 °C; $[\alpha]_D^{22}$ = 34.97° (c 1.0, CH_2Cl_2).

Synthesis of 3n

(3R,3aS,9aR,10aR,10bS,E)-3-(((4-Methoxyphenyl)(methyl)amino)methyl)-6,9a-dimethyl-3a,4,5,8,9,9a,10a,10b-octahydrooxireno[2',3':9,10]cyclodeca[1,2-b]furan-2(3H)-one

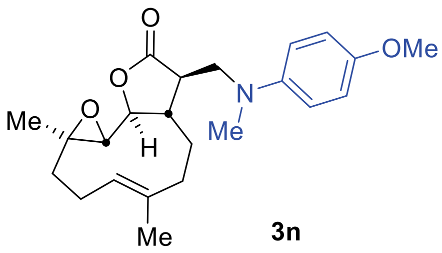

3n

Following the general procedure A, a mixture of parthenolide (80 mg, 0.32 mmol), 4-methoxy-*N*-methylaniline (53 mg, 0.384 mmol) and squaric acid (4 mg, 0.03 mmol) was stirred at 50 °C in 1:1 water-methanol (2 mL) for 48 hours. The title compound was

obtained as a white solid in 36% yield (45 mg). **Rf** = 0.64 (hexane/ethyl acetate, 50%:50%); **^{1}H NMR** (400 MHz, $CDCl_3$) δ 6.90-6.81 (2H, m), 6.79-6.71 (2H, m), 5.08 (1H, dd, *J* 9.8 & 2.1), 3.89 (1H, dd, *J* 15.2 & 4.8), 3.80 (1H, t, *J* 9.2), 3.76 (3H, s), 3.52 (1H, dd, *J* 15.3 & 6.2), 2.93 (3H, s), 2.68 (1H, d, *J* 8.9), 2.62 (1H, ddd, *J* 11.9, 6.0 & 4.8), 2.44-2.28 (1H, m), 2.21-2.07 (4H, m), 1.93 (1H, dd, *J* 14.8 & 6.6), 1.78 (1H, t, *J* 12.7), 1.64 (3H, s), 1.63-1.51 (1H, m), 1.27 (3H, s), 1.25-1.15 (1H, m); **^{13}C NMR** (101 MHz, $CDCl_3$) δ 176.2, 152.2, 144.0, 134.5, 125.0, 115.0, 114.9, 82.3, 66.5, 61.6, 55.8, 53.2, 48.1, 46.4, 41.0, 40.4, 36.6, 30.3, 24.1, 17.2, 16.9; **IR** (neat, cm^{-1}) 1766 (C=O); **HRMS** (TOF MS ASAP+) *m/z*: $[M+H]^+$ calc. for $C_{23}H_{32}NO_4^+$ 386.2331, found 386.2330; **mp** 146-148 °C; **[α]$_D^{22}$** = 81.72° (c 2.0, CH_2Cl_2).

Synthesis of 3o

(3R,3aS,9aR,10aR,10bS,E)-3-(((4-Hydroxyphenyl)(methyl)amino)methyl)-6,9a-dimethyl-3a,4,5,8,9,9a,10a,10b-octahydrooxireno[2',3':9,10]cyclodeca[1,2-b]furan-2(3H)-one

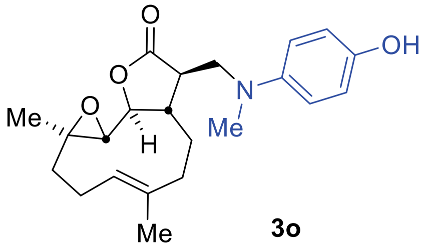

3o

Following the general procedure B, a mixture of aminoparthenolide derivative **3b** (0.114 g, 0.32 mmol), formaldehyde (0.030 g, 0.96 mmol) and sodium triacetoxyborohydride (0.112 g, 0.45 mmol), was stirred at room temperature in dichloroethane (10 mL) for 16 hours. The title compound was obtained as a colourless solid in 61% yield (0.072 g, 0.20 mmol). **Rf** = 0.49 (hexane/ethyl acetate, 50%:50%); **^{1}H NMR** (400 MHz, CD_3OD): δ 6.81-6.71 (4H, m, ArH), 5.14 (1H, dd, *J* 12.4 & 2.3, CH), 3.97 (1H, t, *J* 9.1, CH), 3.83 (1H, dd, *J* 15.0 & 4.9, CH_2), 3.55 (1H, dd, *J* 15.0 & 6.0, CH_2), 2.90 (3H, s, Me), 2.81 (1H, d, *J* 9.1, CH, epoxide moiety), 2.80-2.73 (1H, m, CH), 2.51-2.36 (1H, m, CH_2), 2.32-2.22 (1H, m, CH_2), 2.19-2.03 (3H, m), 1.91 (1H, dd, *J* 14.6 & 6.3, CH_2), 1.79 (1H, t, *J* 12.8, CH_2), 1.71-1.61 (1H, m, CH_2), 1.68 (3H, s, Me), 1.29 (3H, s, Me), 1.22 (1H, td, *J* 12.7 & 5.9, CH_2); **^{13}C NMR** (101 MHz, CD_3OD): δ 175.2 (C=O), 148.9 (Cq), 143.6 (Cq), 134.7 (Cq), 124.3 (CH), 115.6 (CH), 82.4 (CH), 66.7 (CH, epoxide moiety), 61.8 (Cq), 52.9 (CH_2), 46.1 (CH), 40.5 (CH_2), 39.3 (CH_3), 36.2 (CH_2), 29.4 (CH_2), 23.5 (CH_2), 16.0 (CH_3), 15.7 (CH_3); **IR** (neat, cm^{-1}) 3349 (OH), 1761 (C=O); **HRMS** (ES+) m/z: $[M+H]^+$ calc. for $C_{22}H_{30}NO_4^+$: 372.2175, found: 372.2172; **mp**: 192-194 °C; **[α]$_D^{20}$** = -6.58° (c 4, $CHCl_3$).

Synthesis of 6

3-((Phenylamino)methyl)dihydrofuran-2(3H)-one

6

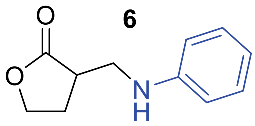

Following the general procedure A, a mixture of tulipane (0.100 g, 1.00 mmol), aniline (0.112 g, 1.20 mmol) and squaric acid (0.011 g, 0.10 mmol), was stirred at 50 °C in 1:1 water-methanol (20 mL) for 48 hours.

The title compound was obtained as a colourless solid in 95% yield (0.181 g, 0.95 mmol). **Rf** = 0.68 (dichloromethane/methanol, 95%:5%); **^{1}H NMR** (400 MHz, $CDCl_3$) : δ 7.22-7.14 (2H, m, ArH), 6.73 (1H, tt, *J* 7.3 & 1.0, ArH), 6.68-6.62 (2H, m, ArH), 4.40-4.32 (1H, td, *J* 8.8 & 2.6, CH_2), 4.26 (1H, br s, NH), 4.24-4.15 (1H, td, *J* 9.8 & 6.7, CH_2), 3.52-3.37 (2H, m, CH_2), 2.92-2.82 (1H, m, CH), 2.45-2.31 (1H, m, CH_2), 2.15-2.05 (1H, m, CH_2); **^{13}C NMR** (101 MHz, $CDCl_3$): δ 178.5 (C=O), 147.6 (C-N), 129.4 (CH), 118.1 (CH), 113.2 (CH), 66.8 (CH_2), 43.9 (CH_2), 39.2 (CH), 26.8 (CH_2); **IR** (neat, cm^{-1}) 3345 (OH), 1765 (C=O); **HRMS** (ES+) m/z: $[M+Na]^+$ calc. for $C_{11}H_{13}NO_2Na^+$: 214.0844, found: 214.0837; **mp**: 60-62 °C.

Synthesis of 7

(3aS,6R,6aR,9R,9aS,9bS)-9-Hydroxy-6-methoxy-6,9-dimethyl-3-methylenedecahydroazuleno[4,5-b]furan-2(3H)-one

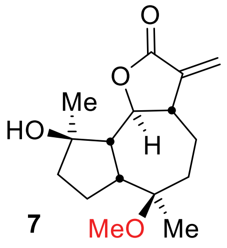

7

To a solution of parthenolide (0.100 g, 0.4 mmol) in 10 mL of methanol was added squaric acid (0.005 g, 0.04 mmol) and the resulting mixture was stirred at room temperature for 3 days. After completion of the reaction, the solvent was removed *in vacuo*, the residual was extracted with ethyl acetate (3 × 25 mL), the combined organic layers were washed with brine and then dried over anhydrous magnesium sulphate, filtered, and solvent removed *in vacuo*. The product was isolated by flash chromatography over silica gel. The title compound was obtained as a colourless solid in 40% yield (0.045 g, 0.16 mmol). **Rf** = 0.46 hexane/ethyl acetate, 50%:50%); **^{1}H NMR** (400 MHz, $CDCl_3$): δ 6.22 (1H, d, *J* 3.5, CH_2), 5.51 (1H, d, *J* 3.3, CH_2), 4.21 (1H, dd, *J* 11.7 & 9.9, CH), 3.19 (3H, s, Me), 2.90-2.72 (2H, m), 2.34 (1H, t, *J* 11.7, CH), 2.33 (1H, br s, OH), 2.21-2.12 (1H, m, CH_2), 2.01-1.93 (1H, m, CH_2), 1.90-1.77 (3H, m), 1.75-1.66 (1H, m, CH_2), 1.60-1.50 (1H, m, CH_2), 1.48-1.37 (1H, m, CH_2), 1.39 (3H, s, Me), 1.16 (3H, s, Me); **^{13}C NMR** (101 MHz, $CDCl_3$): δ 169.6 (C=O), 39.0 (Cq), 120.1 (CH_2), 82.7 (CH), 80.5 (Cq), 78.1 (Cq), 55.5 (CH), 48.3 (CH_3), 46.8 (CH), 46.0 (CH), 39.0 (CH_2), 36.0 (CH_2), 25.5 (CH_2), 24.5 (CH_2), 24.3 (CH_3), 22.6 (CH_3); **IR** (neat, cm^{-1}) 3470 (OH), 1751 (C=O); **HRMS** (ES+) m/z: $[M + Na]^+$ calc. for $C_{16}H_{24}O_4Na^+$: 303.1572, found: 303.1570; **mp**: 105-107 °C; **$[\alpha]_D^{23}$** = -2.77° (c =0.5, CH_2Cl_2).

Synthesis of 8

(3R,3aS,6R,6aR,9R,9aS,9bS)-3-((Dimethylamino)methyl)-9-hydroxy-6-methoxy-6,9-dimethyldecahydroazuleno[4,5-b]furan-2(3H)-one

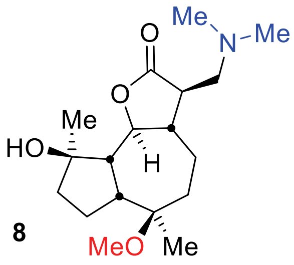

8

A mixture of parthenolide analogue 8 (0.034 g, 0.12 mmol), dimethylamine (0.011 g, 0.24 mmol) and potassium carbonate (0.033 g, 0.24 mmol), was stirred at room temperature in ethanol (10 mL) for 16 hours. The solvent was removed *in vacuo* and the product was isolated by flash chromatography over silica gel as a colourless solid in 89% yield (0.034 g, 0.11 mmol). **Rf** = 0.37 (hexane/ethyl acetate, 50%:50%); **^{1}H NMR** (400 MHz, $CDCl_3$): δ 4.27 (1H, dd, *J* 11.6 & 10.2, CH), 3.17 (3H, s, Me), 2.84-2.74 (1H, m, CH), 2.70 (1H, dd, *J* 12.9 & 5.0, CH_2), 2.55 (1H, dd, *J* 12.9 & 6.6, CH_2), 2.46 (1H, br s, OH), 2.40-2.32 (1H, m, CH), 2.32-2.18 (3H, m), 2.24 (6H, s, Me), 2.11-1.99 (1H, m, CH), 1.95-1.77 (3H, m), 1.66 (1H, td, *J* 13.4 & 3.7, CH_2), 1.60-1.50 (1H, m, CH_2), 1.42 - 1.31 (1H, m, CH_2), 1.34 (3H, s, Me), 1.16 (3H, s, Me); **^{13}C NMR** (101 MHz, $CDCl_3$): δ 176.9 (C=O), 82.9 (CH), 80.2 (Cq), 78.0 (Cq), 58.4 (CH_2), 54.9 (CH), 48.5 (CH), 48.1 (CH_3), 46.0 (CH_3), 45.6 (CH), 44.7 (CH), 39.3 (CH_2), 37.8 (CH_2), 26.0 (CH_2), 25.5 (CH_2), 23.7 (CH_3), 22.0 (CH_3); **IR** (neat, cm^{-1}): 3477 (OH), 1762 (C=O), **HRMS** (ES+) m/z: $[M + H]^+$ calc. for $C_{18}H_{32}NO_4^+$: 326.2331, found: 326.2323; **mp**: 138-140 °C; **$[\alpha]_D^{20}$** = 7.20 (c=1.5, $CHCl_3$).

X-Ray Crystallography

XRD compound 3b

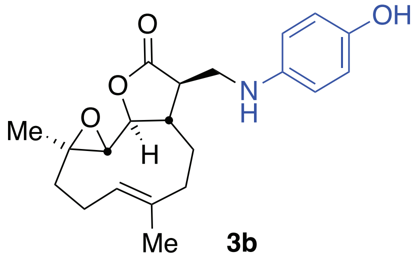

CCDC Deposit Number: *2011606*. Single crystals of $C_{24}H_{33}NO_5$ (**3b** + acetone) were obtained by liquid diffusion of hexane into an acetone solution of **3b**. A suitable crystal was selected and mounted on a SuperNova, Dual, Cu at zero, Atlas diffractometer. The crystal was kept at 99.98(12) K during data collection. Using Olex2,[2] the structure was solved with the ShelXS[3] structure solution program using Direct Methods and refined with the ShelXL[3] refinement package using Least Squares minimisation. The structure contains a molecule of acetone. The hydrogen atoms bonded to N(1) and O(4) were located in the electron density and freely refined. The remaining hydrogen atoms were fixed as riding models.

Crystal Data for $C_{24}H_{33}NO_5$ **3b** (M =415.51 g/mol): monoclinic, space group $P2_1$ (no. 4), a = 11.34175(18) Å, b = 8.21411(13) Å, c = 12.44906(19) Å, β = 107.1578(16)°, V = 1108.17(3) Å3, Z = 2, T = 99.98(12) K, μ(CuKα) = 0.699 mm^{-1}, $Dcalc$ = 1.245 g/cm^3, 39001 reflections measured (7.432° ≤ 2Θ ≤ 148.878°), 4374 unique (R_{int} = 0.0373, R_{sigma} = 0.0165) which were used in all calculations. The final R_1 was 0.0323 (I > 2σ(I)) and wR_2 was 0.0857 (all data). Flack = -0.07 (6).

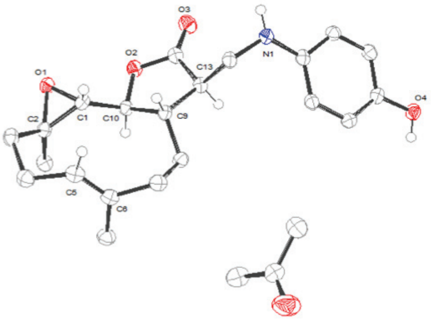

Supplementary Figure 2. *Crystal structure of **3b** with ellipsoids drawn at the 50% probability level. The structure contains a molecule of acetone. Selected atoms labelled and hydrogens attached to non-stereogenic carbons not shown for clarity.*

XRD compound 3d

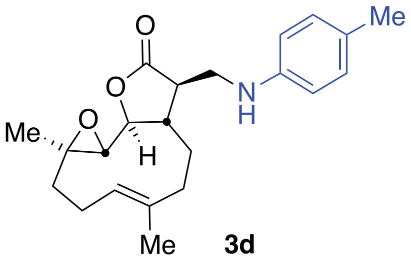

CCDC Deposit Number: *2011607*. Single crystals of $C_{22}H_{29}NO_3$ (**3d**) were obtained by vapour diffusion of hexane into a dichloromethane solution of **3d**. A suitable crystal was selected and mounted on a SuperNova, Dual, Cu at zero, Atlas diffractometer. The crystal was kept at 100.00(10) K during data collection. Using Olex2,[2] the structure was solved with the ShelXT[4] structure solution program using Intrinsic Phasing and refined with the ShelXL[5] refinement package using Least Squares minimisation.

The hydrogen atom bonded to N(1) was located in the electron density and freely refined, while the remaining hydrogen atoms were fixed as riding models with the isotropic thermal parameters (U_{iso}) being based on the U_{eq} of the parent atom.

Crystal Data for $C_{22}H_{29}NO_3$ (M =355.46 g/mol): monoclinic, space group $P2_1$ (no. 4), a = 11.1281(3) Å, b = 7.1835(2) Å, c = 11.8050(3) Å, β = 93.143(2)°, V = 942.26(4) Å^3, Z = 2, T = 100.00(10) K, μ(CuKα) = 0.654 mm^{-1}, $Dcalc$ = 1.253 g/cm^3, 33890 reflections measured (7.5° ≤ 2Θ ≤ 148.854°), 3695 unique (R_{int} = 0.0431, R_{sigma} = 0.0198) which were used in all calculations. The final R_1 was 0.0287 (I > 2σ(I)) and wR_2 was 0.0719 (all data). Flack = -0.03 (8).

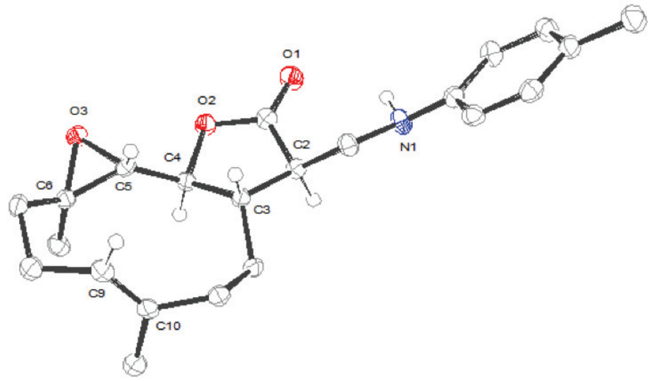

***Supplementary Figure 3.** Crystal structure of **3d** with ellipsoids drawn at the 50% probability level. Selected atoms labelled and hydrogens attached to non-stereogenic carbons not shown for clarity.*

XRD compound 7

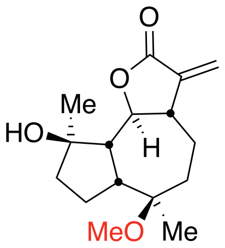

7

CCDC Deposit Number: ***2011605***. Single crystals of $C_{32}H_{50}O_9$ ([**7** x 2]+ H_2O) were obtained by vapour diffusion of hexane into an ethyl acetate solution of **7**. A suitable crystal was selected and mounted on a SuperNova, Dual, Cu at zero, Atlas diffractometer. The crystal was kept at 99.9(5) K during data collection. Using Olex2,[2] the structure was solved with the ShelXS[3] structure solution program using Direct Methods and refined with the ShelXL[5] refinement package using Least Squares minimisation.

The structure contains a molecule of water. The hydrogen atoms bonded to O(3), O(103) and the water molecule O(201) were located in the electron density and freely refined. The remaining hydrogen atoms were fixed as riding models. The structure occupies a chiral space group. The hydrogen atoms bonded to O(3), O(103) and the water molecule O(201) were located in the electron density and freely refined. The remaining hydrogen atoms were fixed as riding models.

Crystal Data for $C_{32}H_{50}O_9$ (M =578.72 g/mol): monoclinic, space group P2$_1$ (no. 4), a = 6.42575(9) Å, b =v25.7445(3) Å, c = 9.53536(12) Å, β = 105.0673(13)°, V = 1523.19(3) Å^3, Z = 2, T = 99.9(5) K, μ(CuKα) = 0.741 mm^{-1}, $Dcalc$ = 1.262 g/cm^3, 28047 reflections measured (6.866° ≤ 2Θ ≤ 138.276°), 5683 unique (R_{int} = 0.0295, R_{sigma} = 0.0200) which were used in all calculations. The final R_1 was 0.0264 (I > 2σ(I)) and wR_2 was 0.0637 (all data). Flack = 0.05 (4).

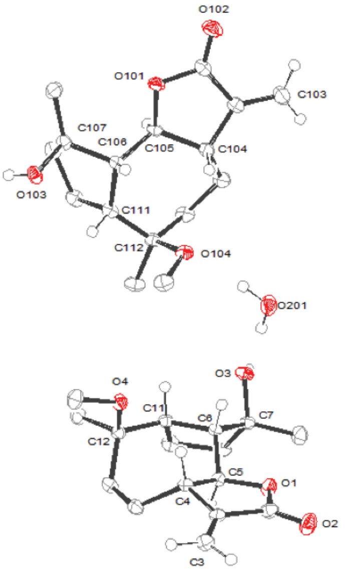

Supplementary Figure 4. *Crystal structure of the asymmetric unit of 7 with ellipsoids drawn at the 50% probability level. The structure contains two molecules of 7 and a molecule of water per asymmetric unit. Selected atoms labelled and hydrogens attached to non-stereogenic carbons not shown for clarity.*

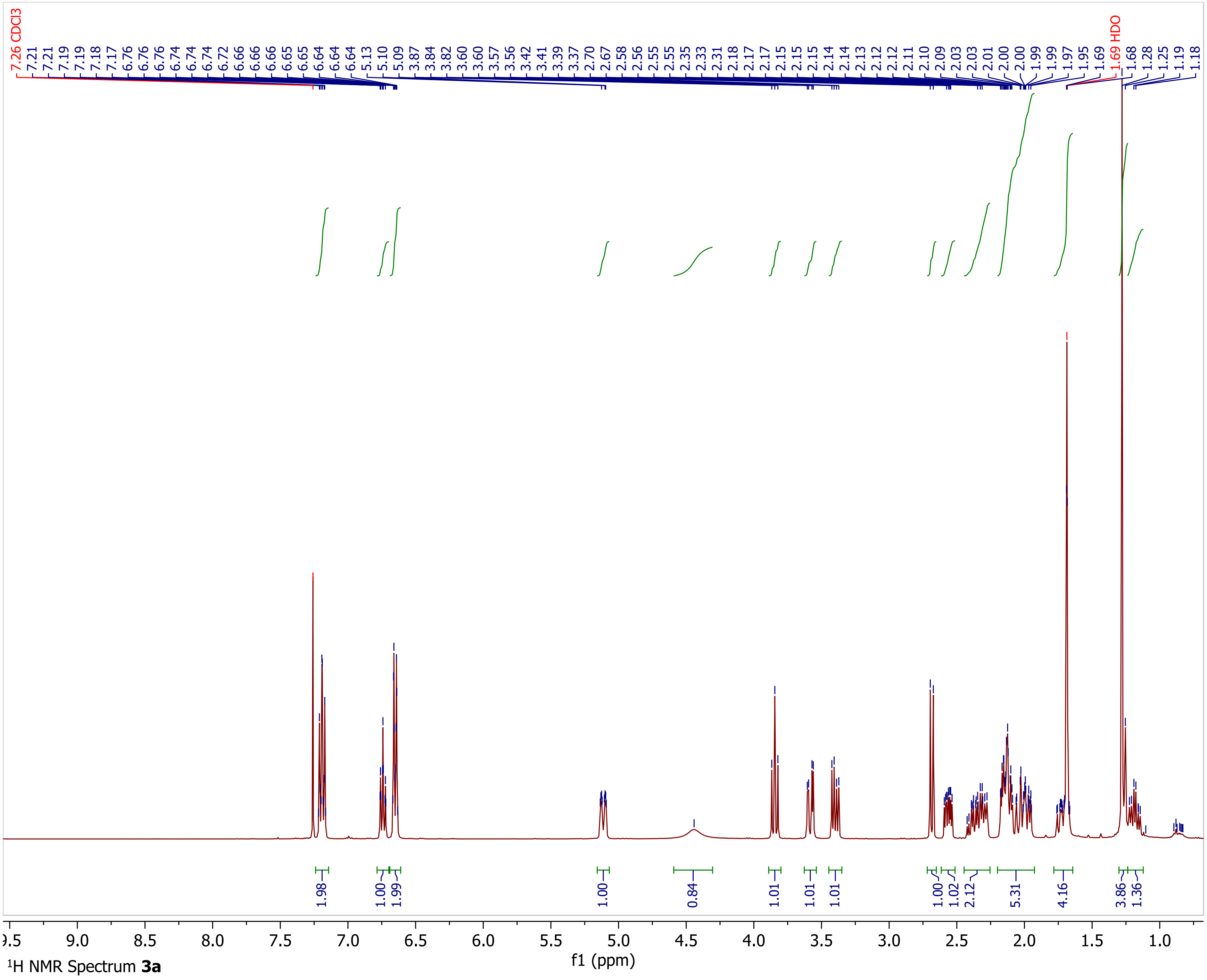

Current Data Parameters
NAME 2017-Jul-13-Fossey-24
EXPNO 10
PROCNO 1

F2 - Acquisition Parameters
Date_ 20170714
Time 5.14
INSTRUM spect
PROBHD 5 mm PADUL 13C
PULPROG zg30
TD 32768
SOLVENT CDCl3
NS 32
DS 2
SWH 8223.685 Hz
FIDRES 0.250967 Hz
AQ 1.9922944 sec
RG 64
DW 60.800 usec
DE 16.65 usec
TE 295.1 K
D1 1.50000000 sec
TD0 1

======== CHANNEL f1 ========
SFO1 400.1324008 MHz
NUC1 1H
P1 11.06 usec
PLW1 24.29199982 W

F2 - Processing parameters
SI 32768
SF 400.1300105 MHz
WDW EM
SSB 0
LB 0.30 Hz
GB 0
PC 1.00

^{1}H NMR Spectrum **3a**

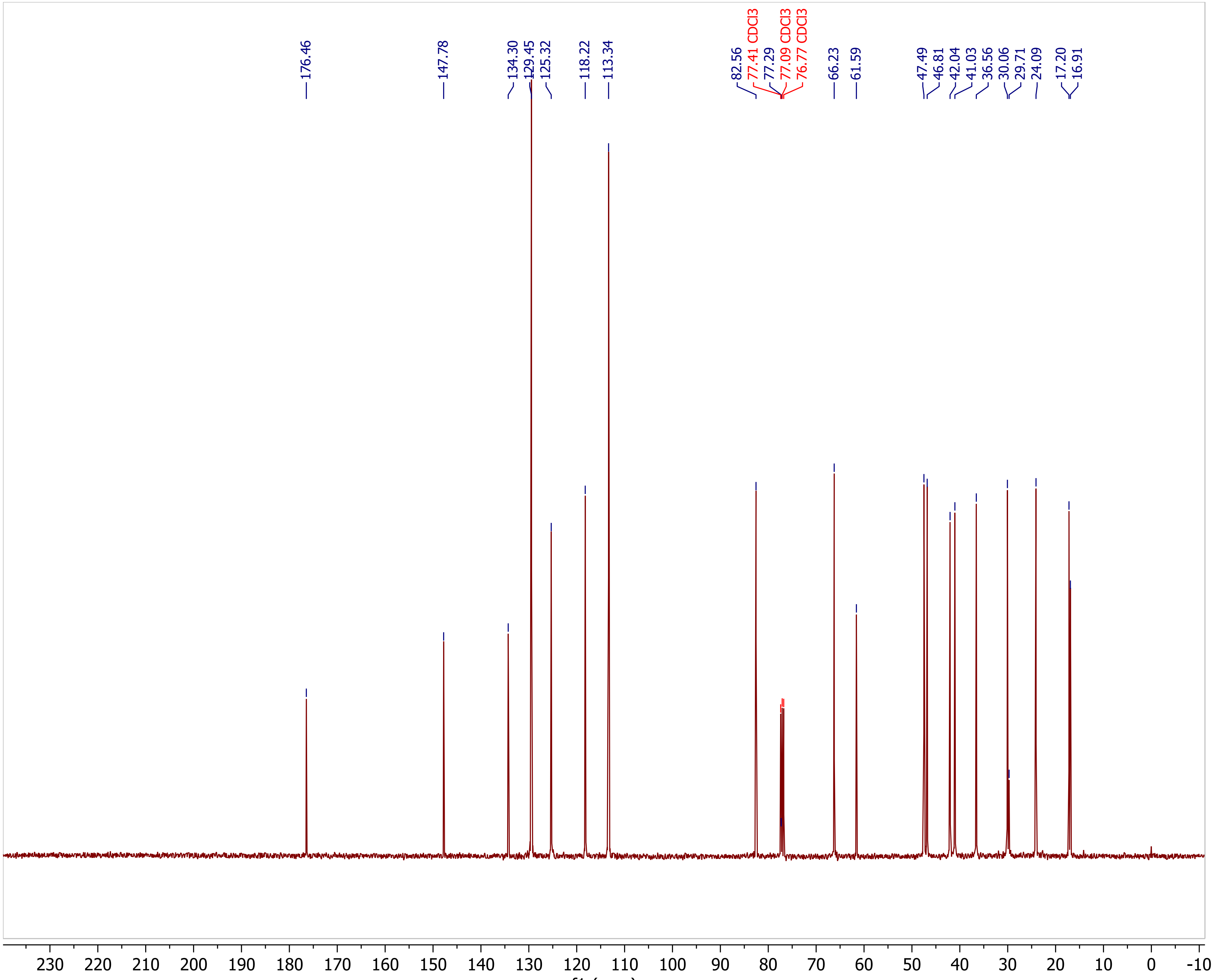

^{13}C NMR Spectrum **3a**

Current Data Parameters
NAME 2017-Jul-13-Fossey-24
EXPNO 12
PROCNO 1

F2 - Acquisition Parameters
Date_ 20170714
Time 5.51
INSTRUM spect
PROBHD 5 mm PADUL 13C
PULPROG udeft
TD 18178
SOLVENT CDCl3
NS 380
DS 0
SWH 25252.525 Hz
FIDRES 1.389181 Hz
AQ 0.3599244 sec
RG 2050
DW 19.800 usec
DE 8.20 usec
TE 295.5 K
D1 3.00000000 sec
D11 0.03000000 sec
D12 0.00002000 sec
D20 200.00000000 sec
TD0 380

======== CHANNEL f1 ========
SFO1 100.6242690 MHz
NUC1 13C
P1 8.80 usec
P13 2000.00 usec
P26 500.00 usec
PLW1 58.63899994 W
SPNAM[5] Crp60comp.4
SPOAL5 0.500
SPOFFS5 0 Hz
SPW5 6.93809986 W
SPNAM[8] Crp60,0.5,20.1
SPOAL8 0.500
SPOFFS8 0 Hz
SPW8 6.93809986 W

======== CHANNEL f2 ========
SFO2 400.1320000 MHz
NUC2 1H
CPDPRG[2 waltz16
PCPD2 90.00 usec
PLW2 24.29199982 W
PLW12 0.28218001 W

F2 - Processing parameters
SI 65536
SF 100.6127690 MHz
WDW EM
SSB 0

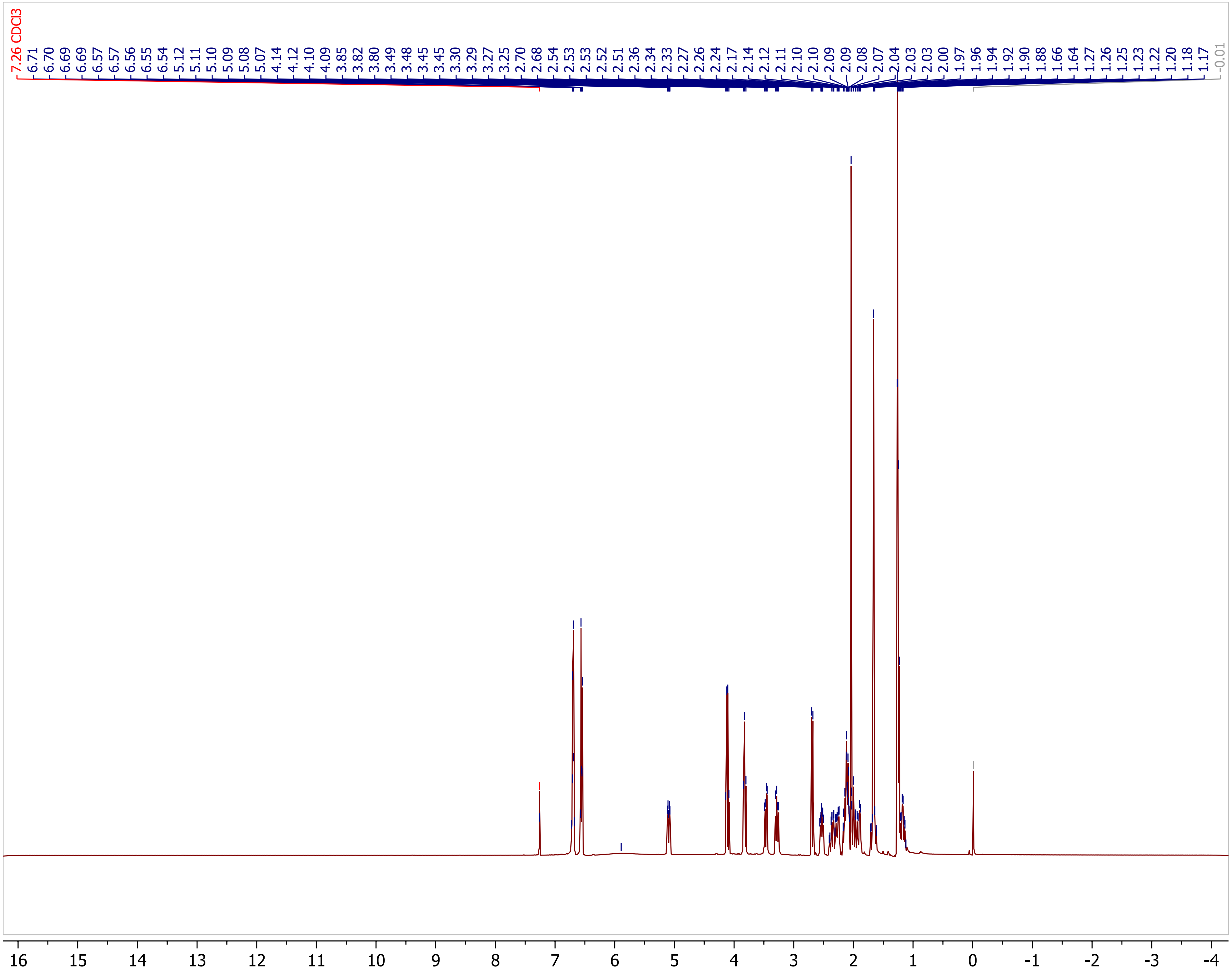

Current Data Parameters
NAME 07-06-Fossey-23
EXPNO 11
PROCNO 1

F2 - Acquisition Parameters
Date_ 20150707
Time 12.12
INSTRUM spect
PROBHD 5 mm PADUL 13C
PULPROG zg30
TD 32768
SOLVENT CDCl3
NS 32
DS 2
SWH 8223.685 Hz
FIDRES 0.250967 Hz
AQ 1.9922944 sec
RG 114
DW 60.800 usec
DE 16.98 usec
TE 293.1 K
D1 1.50000000 sec
TD0 1

======== CHANNEL f1 ========
SFO1 400.1324008 MHz
NUC1 1H
P1 9.50 usec
PLW1 24.29199982 W

F2 - Processing parameters
SI 32768
SF 400.1300045 MHz
WDW EM
SSB 0
LB 0.30 Hz
GB 0
PC 1.00

^{1}H NMR Spectrum **3b**

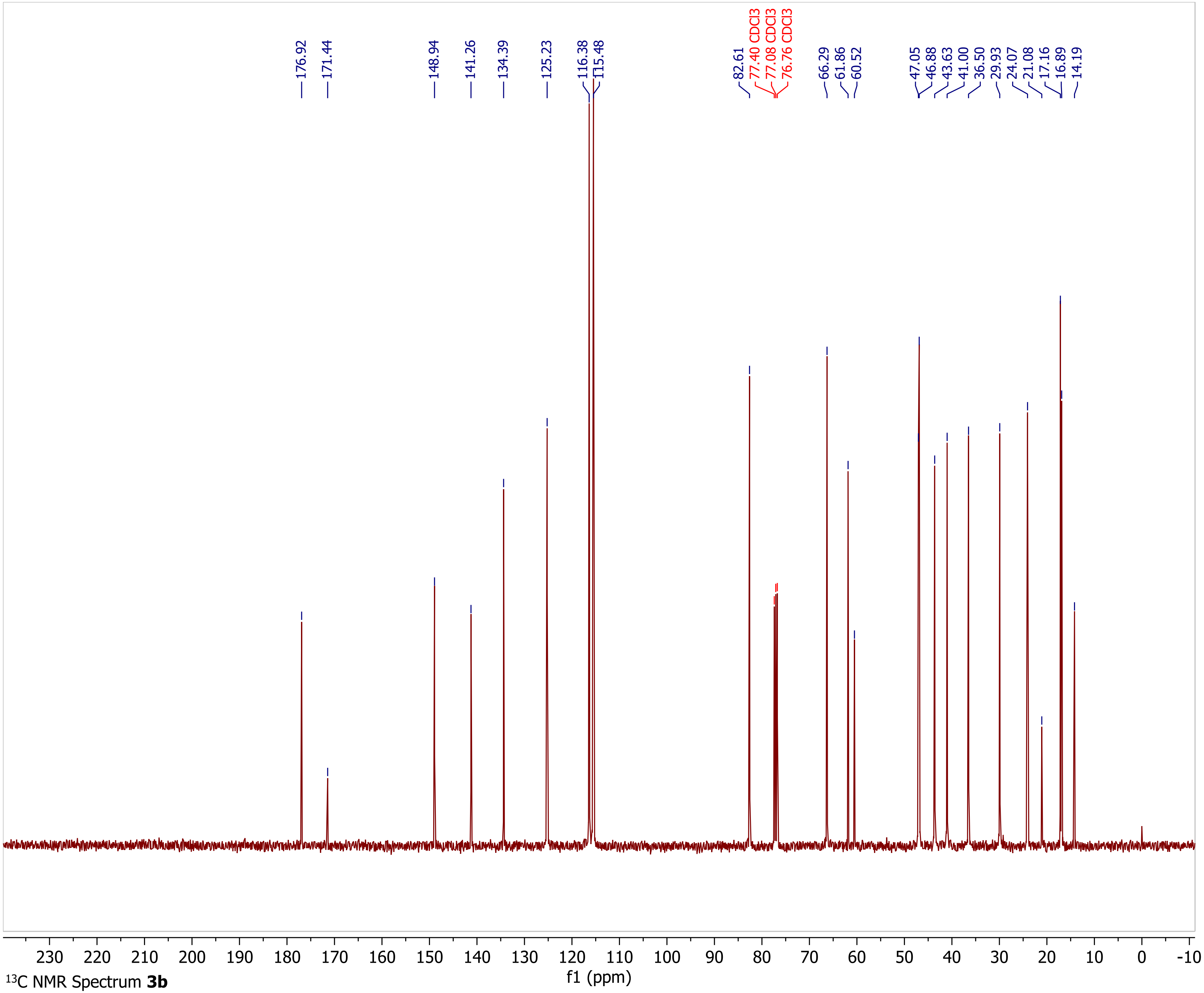

Current Data Parameters
NAME 07-06-Fossey-23
EXPNO 15
PROCNO 1

F2 - Acquisition Parameters
Date_ 20150707
Time 13.49
INSTRUM spect
PROBHD 5 mm PADUL 13C
PULPROG udeft
TD 18178
SOLVENT CDCl3
NS 380
DS 0
SWH 25252.525 Hz
FIDRES 1.389181 Hz
AQ 0.3599244 sec
RG 2050
DW 19.800 usec
DE 8.20 usec
TE 293.2 K
D1 3.00000000 sec
D11 0.03000000 sec
D12 0.00002000 sec
D20 200.00000000 sec
TD0 380

======== CHANNEL f1 ========
SFO1 100.6242690 MHz
NUC1 13C
P1 8.80 usec
P13 2000.00 usec
P26 500.00 usec
PLW1 58.63899994 W
SPNAM[5] Crp60comp.4
SPOAL5 0.500
SPOFFS5 0 Hz
SPW5 6.93809986 W
SPNAM[8] Crp60,0.5,20.1
SPOAL8 0.500
SPOFFS8 0 Hz
SPW8 6.93809986 W

======== CHANNEL f2 ========
SFO2 400.1320000 MHz
NUC2 1H
CPDPRG[2 waltz16
PCPD2 90.00 usec
PLW2 24.29199982 W
PLW12 0.28218001 W

F2 - Processing parameters
SI 65536
SF 100.6127690 MHz
WDW EM
SSB 0
LB 2.00 Hz
GB 0
PC 1.00

^{13}C NMR Spectrum **3b**

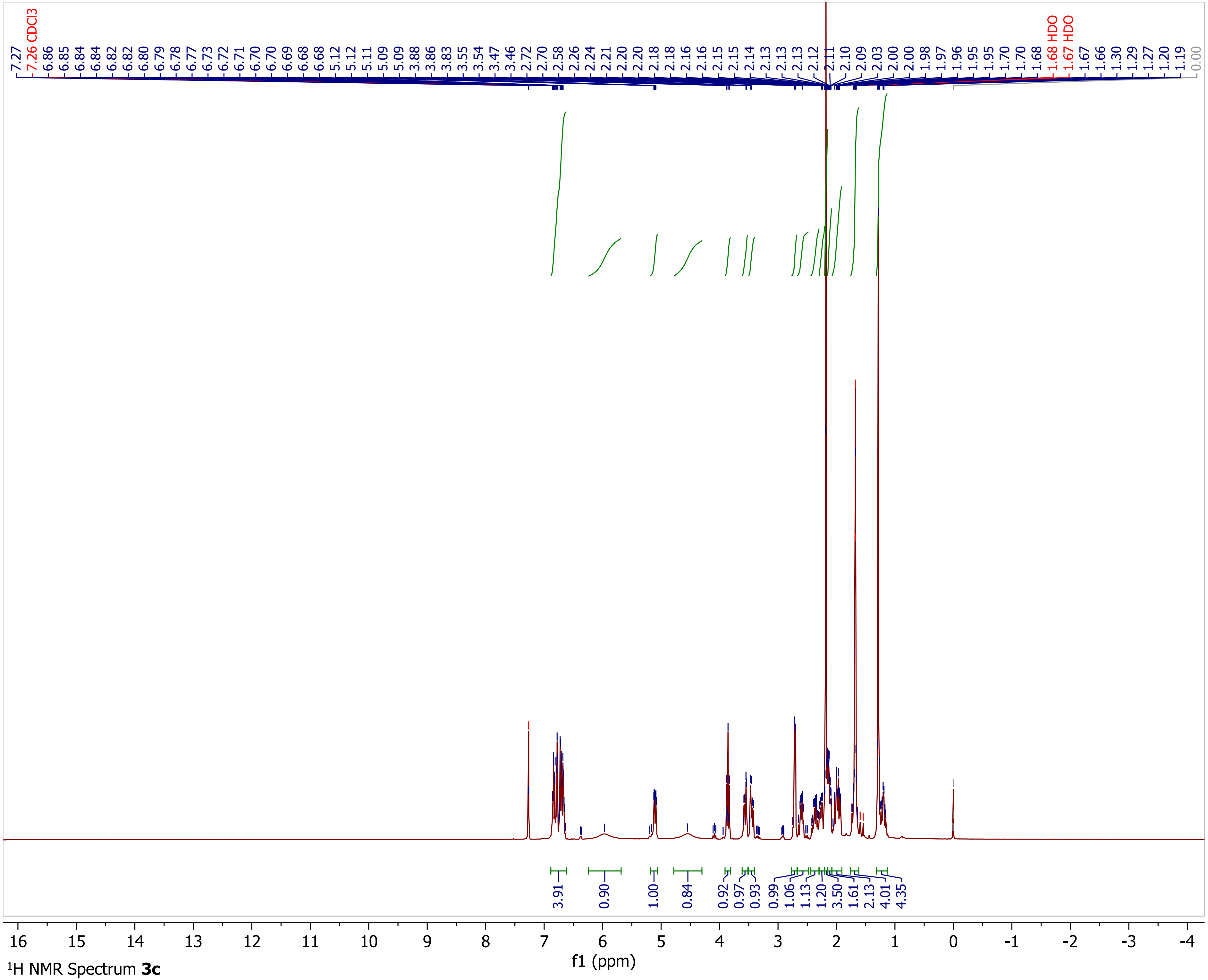

Current Data Parameters
NAME 2017-Nov-05-Fossey-10
EXPNO 10
PROCNO 1

F2 - Acquisition Parameters
Date_ 20171105
Time 17.42
INSTRUM spect
PROBHD 5 mm PADUL 13C
PULPROG zg30
TD 32768
SOLVENT CDCl3
NS 32
DS 2
SWH 8223.685 Hz
FIDRES 0.250967 Hz
AQ 1.9922944 sec
RG 203
DW 60.800 usec
DE 16.65 usec
TE 295.2 K
D1 1.50000000 sec
TD0 1

======== CHANNEL f1 ========
SFO1 400.1324008 MHz
NUC1 1H
P1 11.06 usec
PLW1 24.29199982 W

F2 - Processing parameters
SI 32768
SF 400.1300081 MHz
WDW EM
SSB 0
LB 0.30 Hz
GB 0

^{1}H NMR Spectrum **3c**

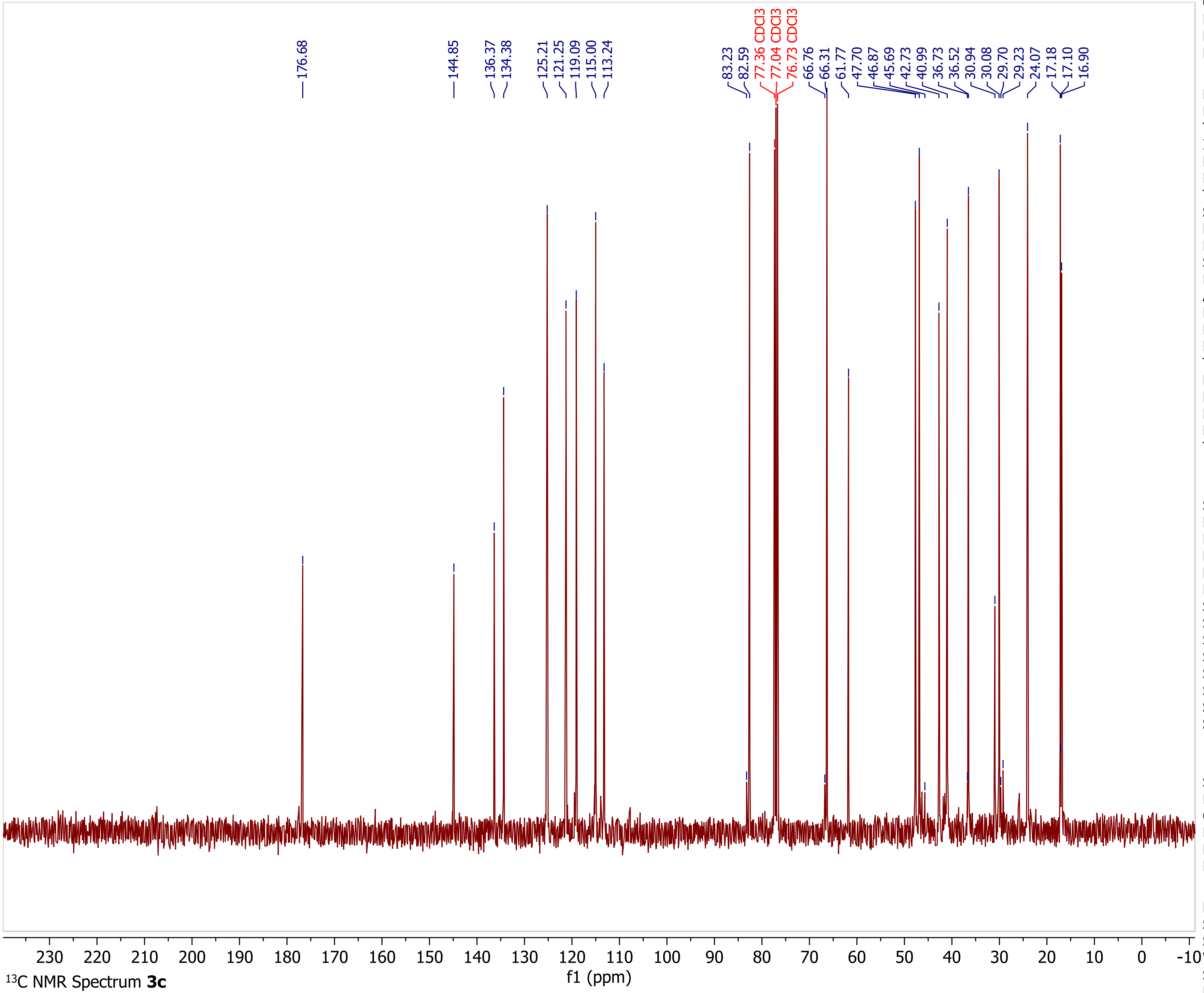

^{13}C NMR Spectrum **3c**

Current Data Parameters
NAME 2017-Nov-05-Fossey-10
EXPNO 12
PROCNO 1

F2 - Acquisition Parameters
Date_ 20171105
Time 18.22
INSTRUM spect
PROBHD 5 mm PADUL 13C
PULPROG udeft
TD 18178
SOLVENT CDCl3
NS 380
DS 0
SWH 25252.525 Hz
FIDRES 1.389181 Hz
AQ 0.3599244 sec
RG 2050
DW 19.800 usec
DE 8.20 usec
TE 295.6 K
D1 3.00000000 sec
D11 0.03000000 sec
D12 0.00002000 sec
D20 200.00000000 sec
TD0 380

======== CHANNEL f1 =======
SFO1 100.6242690 MHz
NUC1 13C
P1 8.80 usec
P13 2000.00 usec
P26 500.00 usec
PLW1 58.63899994 W
SPNAM[5] Crp60comp.4
SPOAL5 0.500
SPOFFS5 0 Hz
SPW5 6.93809986 W
SPNAM[8] Crp60,0.5,20.1
SPOAL8 0.500
SPOFFS8 0 Hz
SPW8 6.93809986 W

======== CHANNEL f2 =======
SFO2 400.1320000 MHz
NUC2 1H
CPDPRG[2 waltz16
PCPD2 90.00 usec
PLW2 24.29199982 W
PLW12 0.28218001 W

F2 - Processing parameters
SI 65536
SF 100.6127690 MHz
WDW EM
SSB 0
LB 2.00 Hz

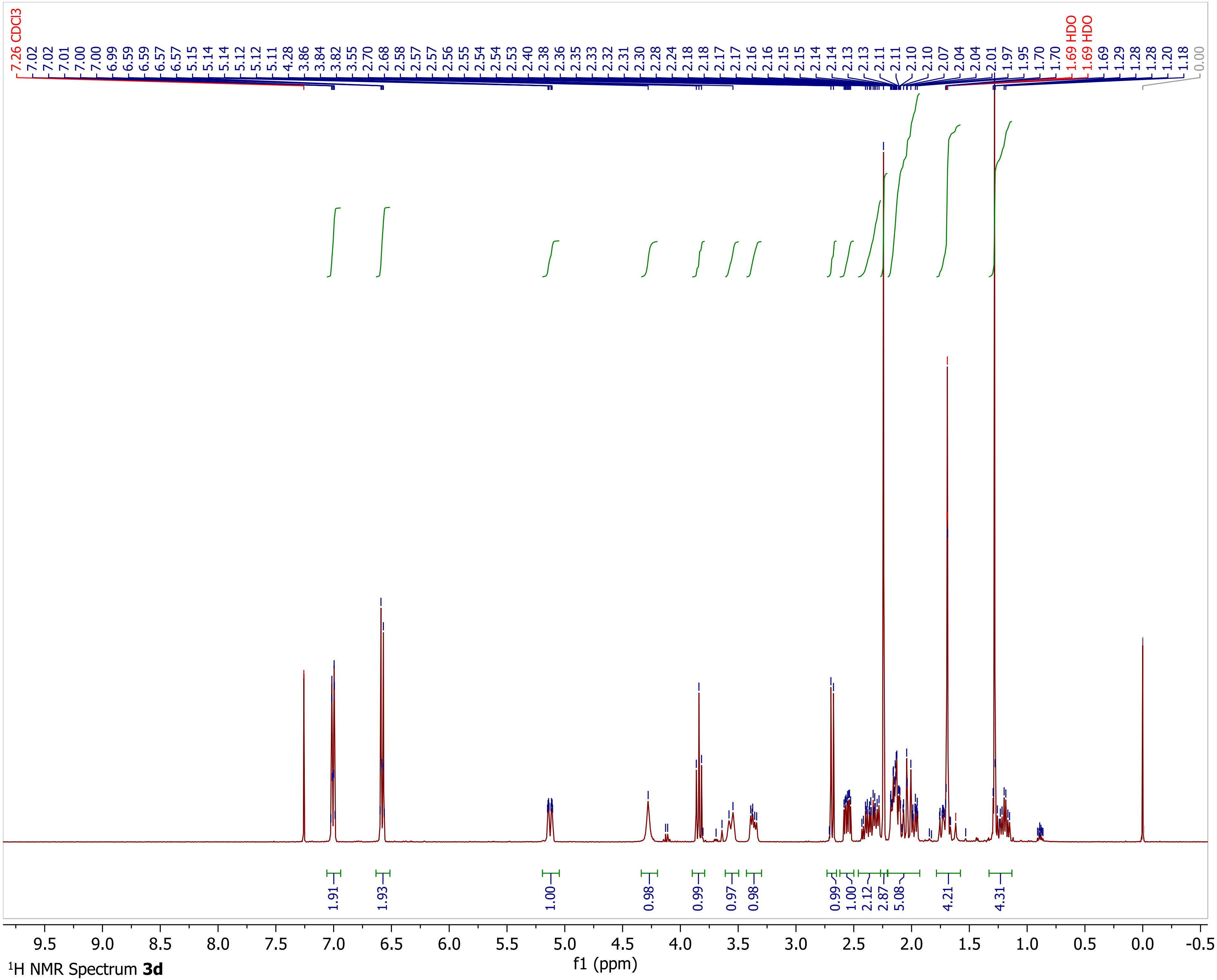

Current Data Parameters
NAME AQ-37B F7-10
EXPNO 10
PROCNO 1

F2 - Acquisition Parameters
Date_ 20190214
Time 16.27 h
INSTRUM AvanceNeo
PROBHD Z116098_0793 (
PULPROG zg30
TD 65536
SOLVENT CDCl3
NS 2
DS 0
SWH 7142.857 Hz
FIDRES 0.217983 Hz
AQ 4.5875201 sec
RG 70.5882
DW 70.000 usec
DE 14.62 usec
TE 298.0 K
D1 2.00000000 sec
TD0 1
SFO1 400.1324008 MHz
NUC1 1H
P0 3.33 usec
P1 10.00 usec
PLW1 18.69700050 W

F2 - Processing parameters
SI 131072
SF 400.1300105 MHz
WDW EM
SSB 0
LB 0.10 Hz
GB 0
PC 1.00

^{1}H NMR Spectrum **3d**

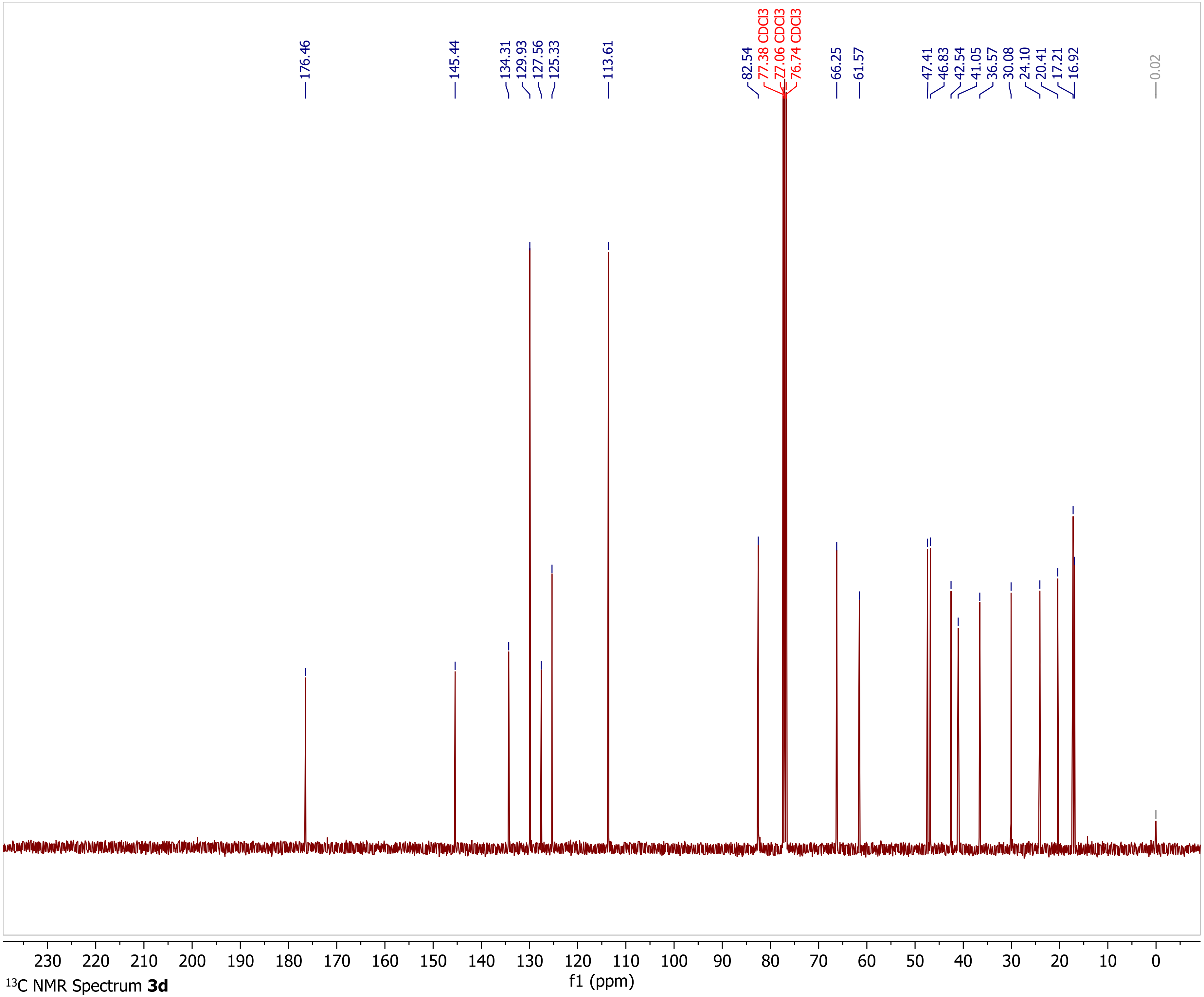

Current Data Parameters
NAME AQ-37B F7-10
EXPNO 11
PROCNO 1

F2 - Acquisition Parameters
Date_ 20190214
Time 21.03 h
INSTRUM AvanceNeo
PROBHD Z116098_0793 (
PULPROG zgpg30
TD 119044
SOLVENT CDCl3
NS 512
DS 0
SWH 25000.000 Hz
FIDRES 0.420013 Hz
AQ 2.3808801 sec
RG 31.9602
DW 20.000 usec
DE 7.12 usec
TE 298.0 K
D1 1.00000000 sec
D11 0.03000000 sec
TD0 1
SFO1 100.6243390 MHz
NUC1 13C
P0 3.33 usec
P1 10.00 usec
PLW1 83.92700195 W
SFO2 400.1318006 MHz
NUC2 1H
CPDPRG[2 waltz64
PCPD2 90.00 usec
PLW2 18.69700050 W
PLW12 0.23083000 W
PLW13 0.11611000 W

F2 - Processing parameters

^{13}C NMR Spectrum **3d**

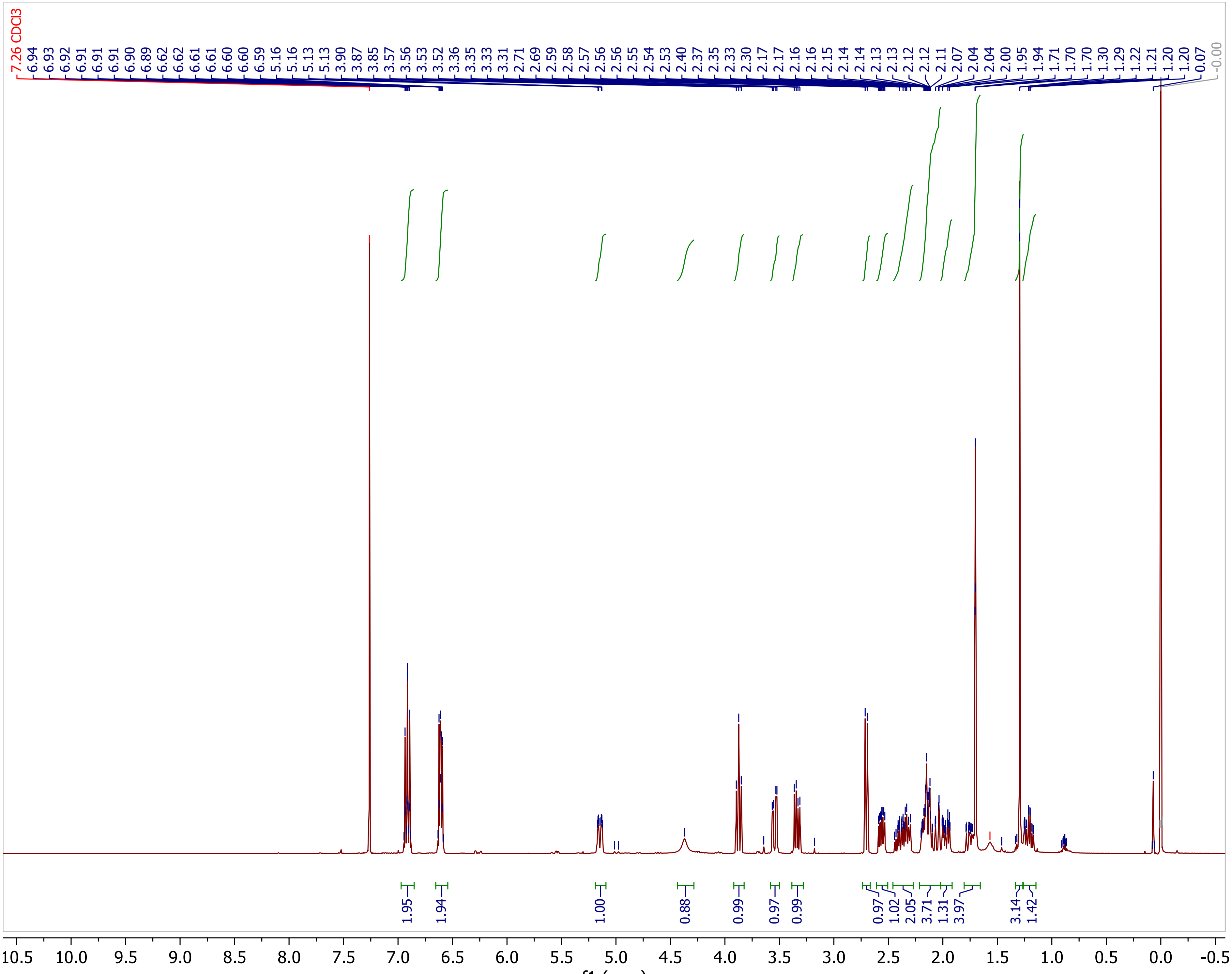

Current Data Parameters
NAME AQ-38 X2
EXPNO 10
PROCNO 1

F2 - Acquisition Parameters
Date_ 20190405
Time 0.04
INSTRUM spect
PROBHD 5 mm PADUL 13C
PULPROG zg
TD 32768
SOLVENT CDCl3
NS 64
DS 2
SWH 8223.685 Hz
FIDRES 0.250967 Hz
AQ 1.9922944 sec
RG 203
DW 60.800 usec
DE 11.93 usec
TE 294.5 K
D1 15.00000000 sec
TD0 1

======== CHANNEL f1 ========
SFO1 400.1324008 MHz
NUC1 1H
P1 11.06 usec
PLW1 24.29199982 W

F2 - Processing parameters
SI 32768
SF 400.1300088 MHz
WDW EM
SSB 0
[illegible]

^{1}H NMR Spectrum **3e**

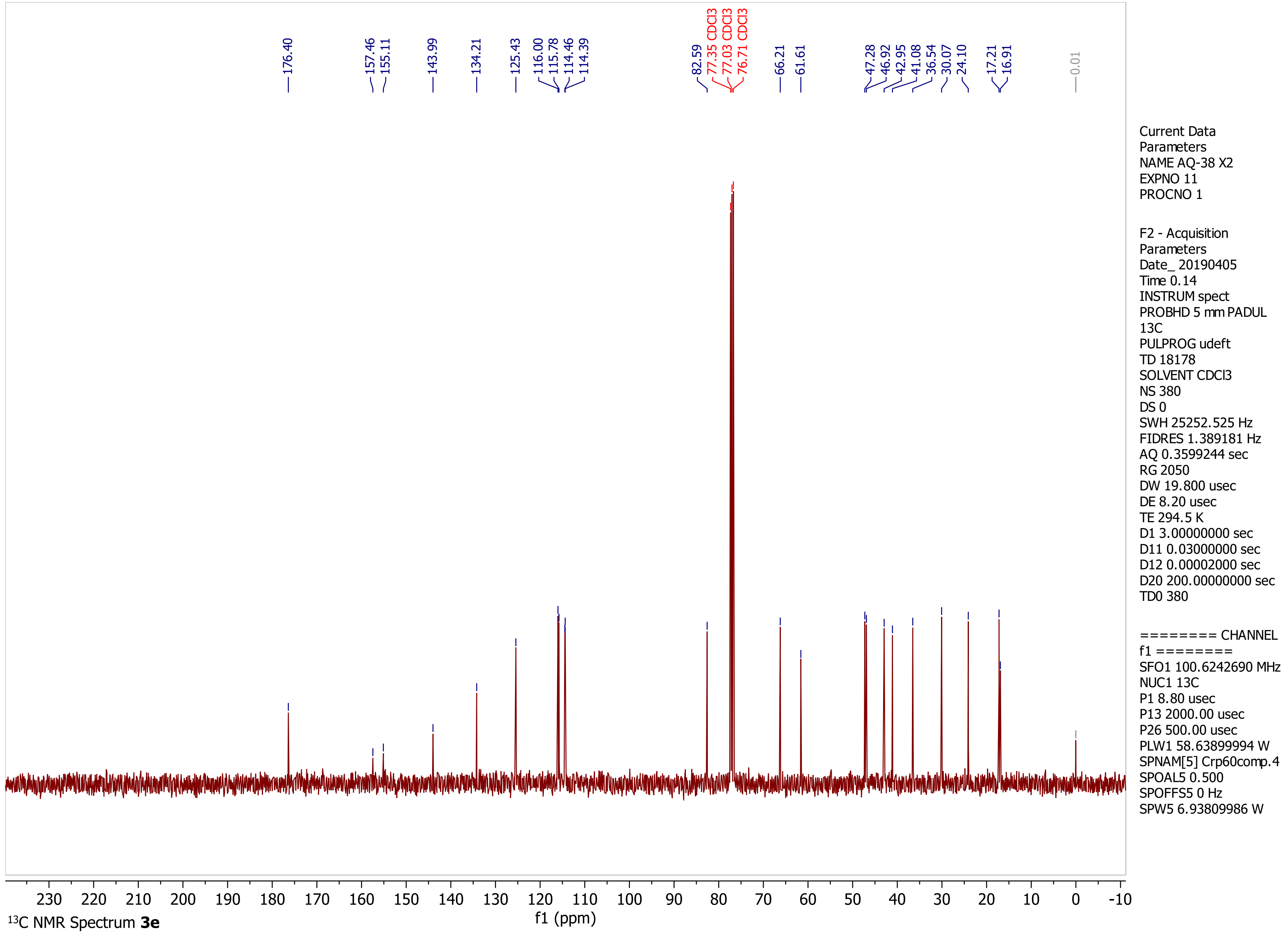

^{13}C NMR Spectrum **3e**

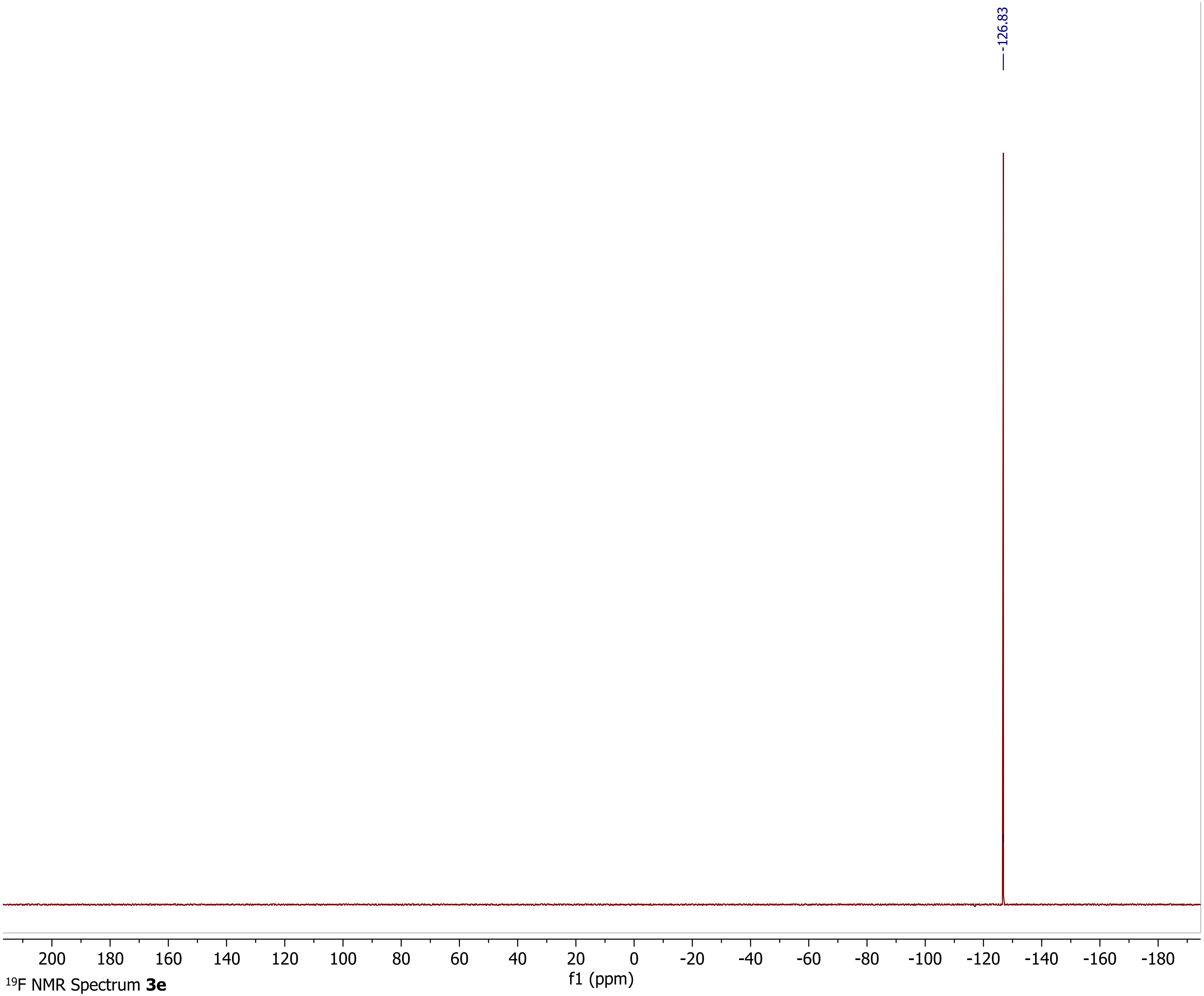

Current Data Parameters
NAME AQ-38 F14-21
EXPNO 11
PROCNO 1

F2 - Acquisition Parameters
Date_ 20190215
Time 13.54 h
INSTRUM AvanceNeo
PROBHD Z116098_0793 (
PULPROG zgig30
TD 261948
SOLVENT CDCl3
NS 16
DS 0
SWH 156250.000 Hz
FIDRES 1.192985 Hz
AQ 0.8382336 sec
RG 101
DW 3.200 usec
DE 6.82 usec
TE 298.0 K
D1 2.00000000 sec
D11 0.03000000 sec
TD0 1
SFO1 376.5021312 MHz
NUC1 19F
P0 6.00 usec
P1 18.00 usec
PLW1 18.94099998 W
SFO2 400.1318006 MHz
NUC2 1H
CPDPRG[2 waltz16
PCPD2 90.00 usec
PLW2 18.69700050 W
PLW12 0.23083000 W

F2 - Processing parameters
SI 262144
SF 376.4983662 MHz
WDW EM
SSB 0
LB 1.00 Hz
GB 0
PC 2.00

^{19}F NMR Spectrum **3e**

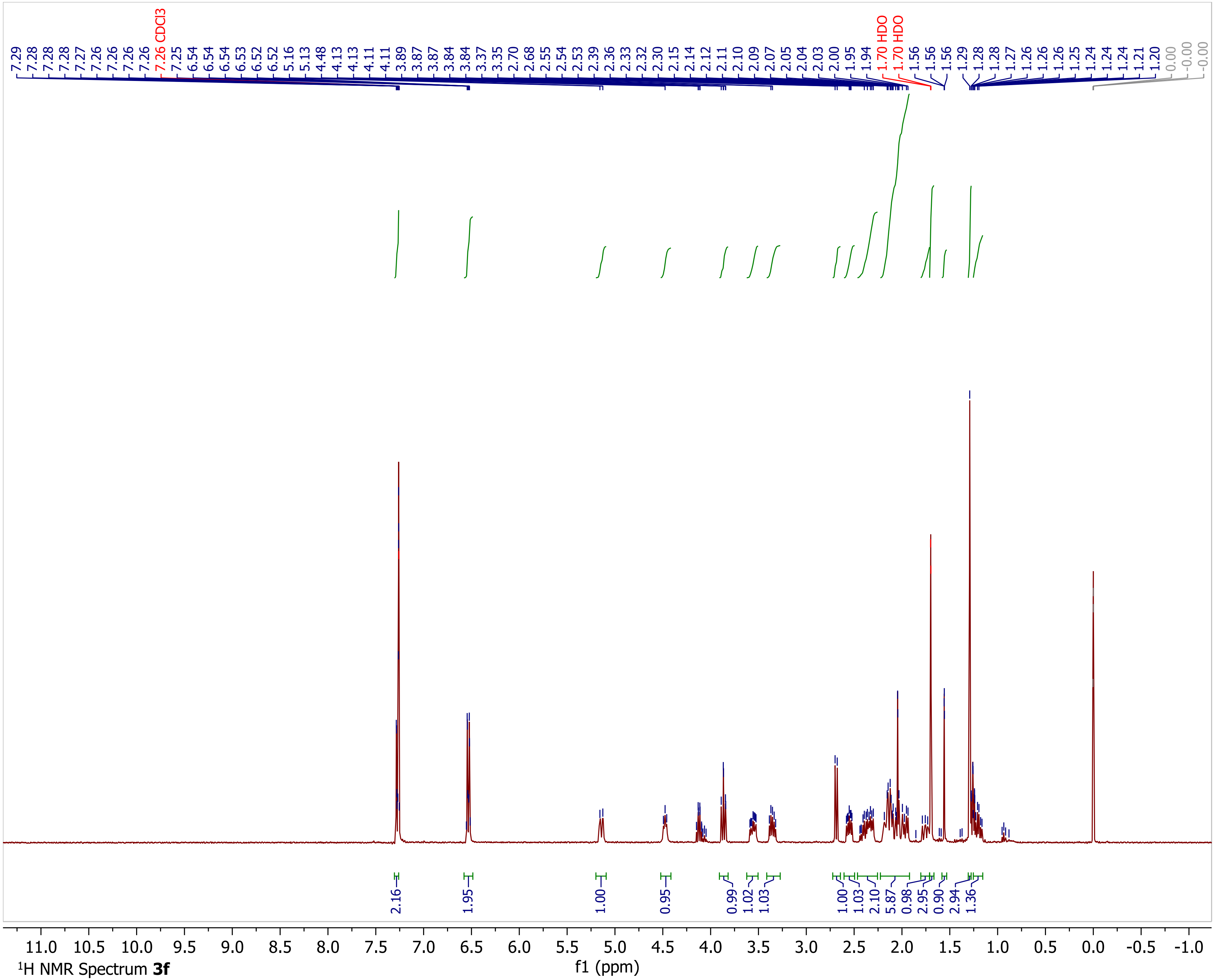

Current Data Parameters
NAME AQ-39B F7-8
EXPNO 10
PROCNO 1

F2 - Acquisition Parameters
Date_ 20190222
Time 15.26 h
INSTRUM AvanceNeo
PROBHD Z116098_0793 (
PULPROG zg30
TD 65536
SOLVENT CDCl3
NS 2
DS 0
SWH 7142.857 Hz
FIDRES 0.217983 Hz
AQ 4.5875201 sec
RG 101
DW 70.000 usec
DE 14.62 usec
TE 298.0 K
D1 2.00000000 sec
TD0 1
SFO1 400.1324008 MHz
NUC1 1H
P0 3.33 usec
P1 10.00 usec
PLW1 18.69700050 W

F2 - Processing parameters
SI 131072
SF 400.1300096 MHz
WDW EM
SSB 0
LB 0.10 Hz
GB 0
PC 1.00

^{1}H NMR Spectrum **3f**

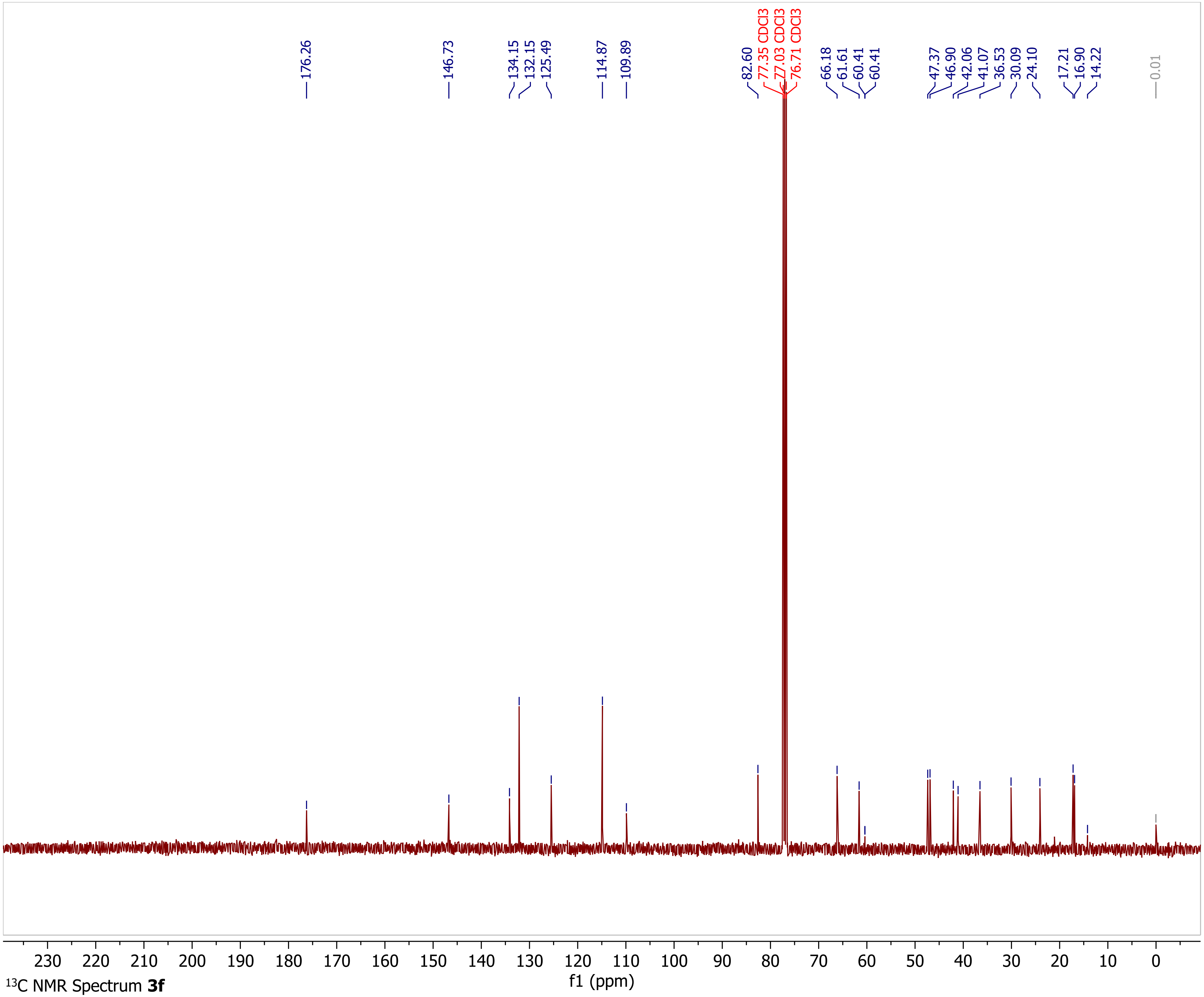

Current Data Parameters
NAME AQ-39B F7-8
EXPNO 11
PROCNO 1

F2 - Acquisition Parameters
Date_ 20190224
Time 19.18 h
INSTRUM AvanceNeo
PROBHD Z116098_0793 (
PULPROG zgpg30
TD 119044
SOLVENT CDCl3
NS 512
DS 0
SWH 25000.000 Hz
FIDRES 0.420013 Hz
AQ 2.3808801 sec
RG 31.9602
DW 20.000 usec
DE 7.12 usec
TE 298.0 K
D1 1.00000000 sec
D11 0.03000000 sec
TD0 1
SFO1 100.6243390 MHz
NUC1 13C
P0 3.33 usec
P1 10.00 usec
PLW1 83.92700195 W
SFO2 400.1318006 MHz
NUC2 1H
CPDPRG[2 waltz64
PCPD2 90.00 usec
PLW2 18.69700050 W
PLW12 0.23083000 W
PLW13 0.11611000 W

F2 - Processing parameters
SI 131072
SF 100.6127685 MHz
WDW EM
SSB 0
LB 1.00 Hz
GB 0
PC 1.40

^{13}C NMR Spectrum **3f**

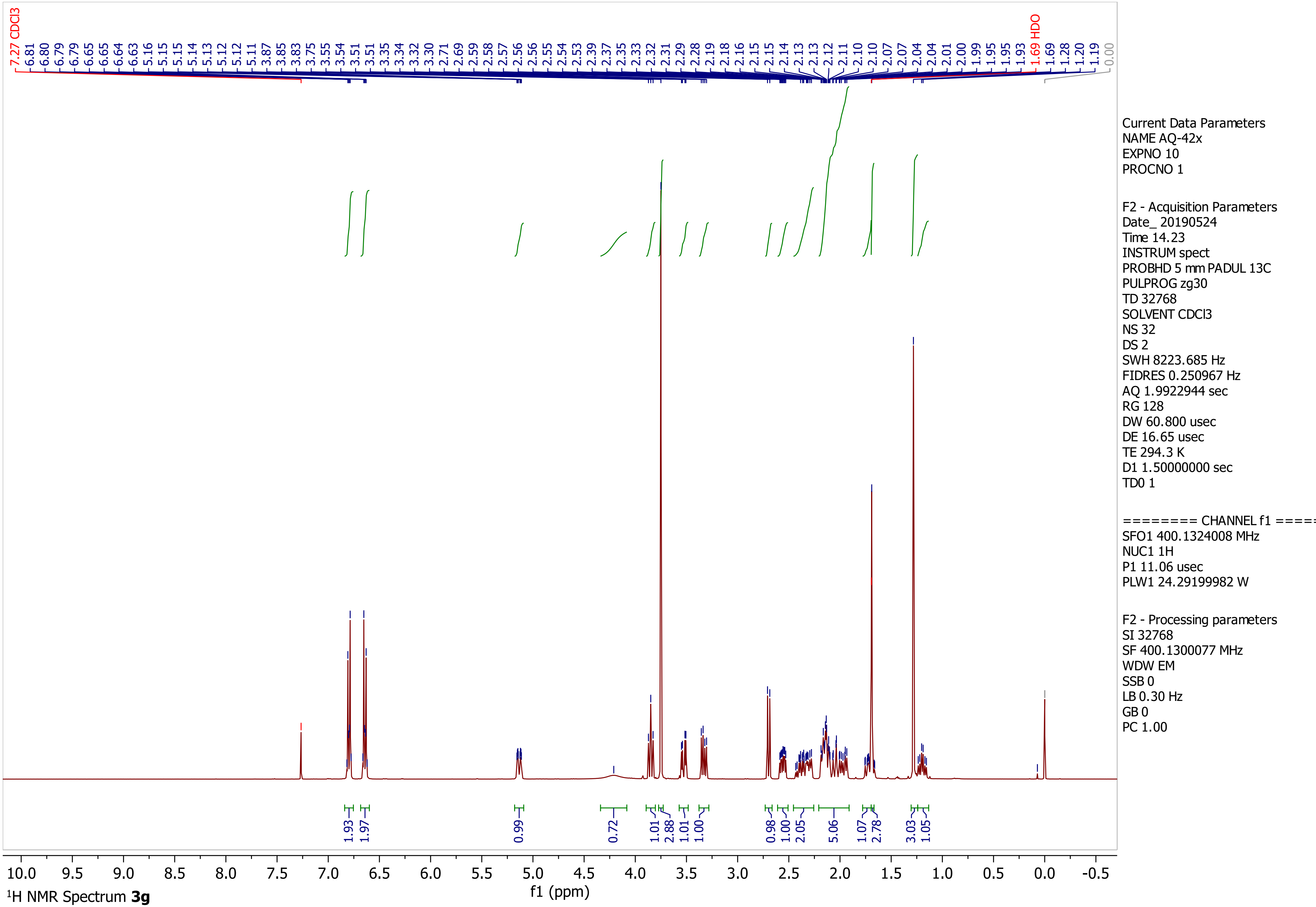

^{1}H NMR Spectrum **3g**

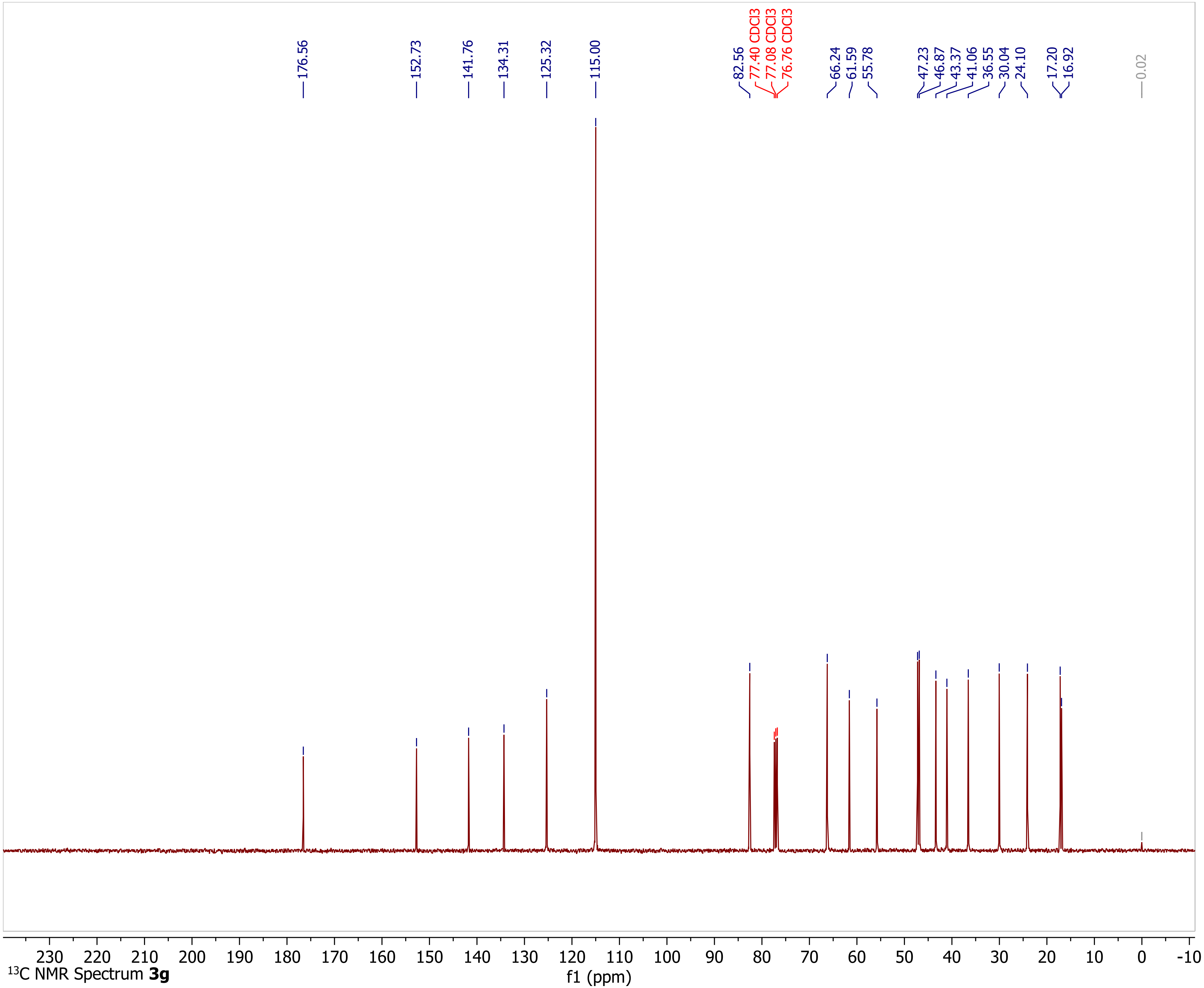

^{13}C NMR Spectrum **3g**

Current Data Parameters
NAME AQ-42x
EXPNO 11
PROCNO 1

F2 - Acquisition Parameters
Date_ 20190524
Time 14.33
INSTRUM spect
PROBHD 5 mm PADUL 13C
PULPROG udeft
TD 18178
SOLVENT CDCl3
NS 380
DS 0
SWH 25252.525 Hz
FIDRES 1.389181 Hz
AQ 0.3599244 sec
RG 2050
DW 19.800 usec
DE 8.20 usec
TE 295.0 K
D1 3.00000000 sec
D11 0.03000000 sec
D12 0.00002000 sec
D20 200.00000000 sec
TD0 380

======== CHANNEL f1 ========
SFO1 100.6242690 MHz
NUC1 13C
P1 8.80 usec
P13 2000.00 usec
P26 500.00 usec
PLW1 58.63899994 W
SPNAM[5] Crp60comp.4
SPOAL5 0.500
SPOFFS5 0 Hz
SPW5 6.93809986 W
SPNAM[8] Crp60,0.5,20.1
SPOAL8 0.500
SPOFFS8 0 Hz
SPW8 6.93809986 W

======== CHANNEL f2 ========
SFO2 400.1320000 MHz
NUC2 1H
CPDPRG[2 waltz16
PCPD2 90.00 usec
PLW2 24.29199982 W
PLW12 0.28218001 W

F2 - Processing parameters
SI 65536
SF 100.6127690 MHz
WDW EM
SSB 0
LB 2.00 Hz
GB 0
PC 1.00

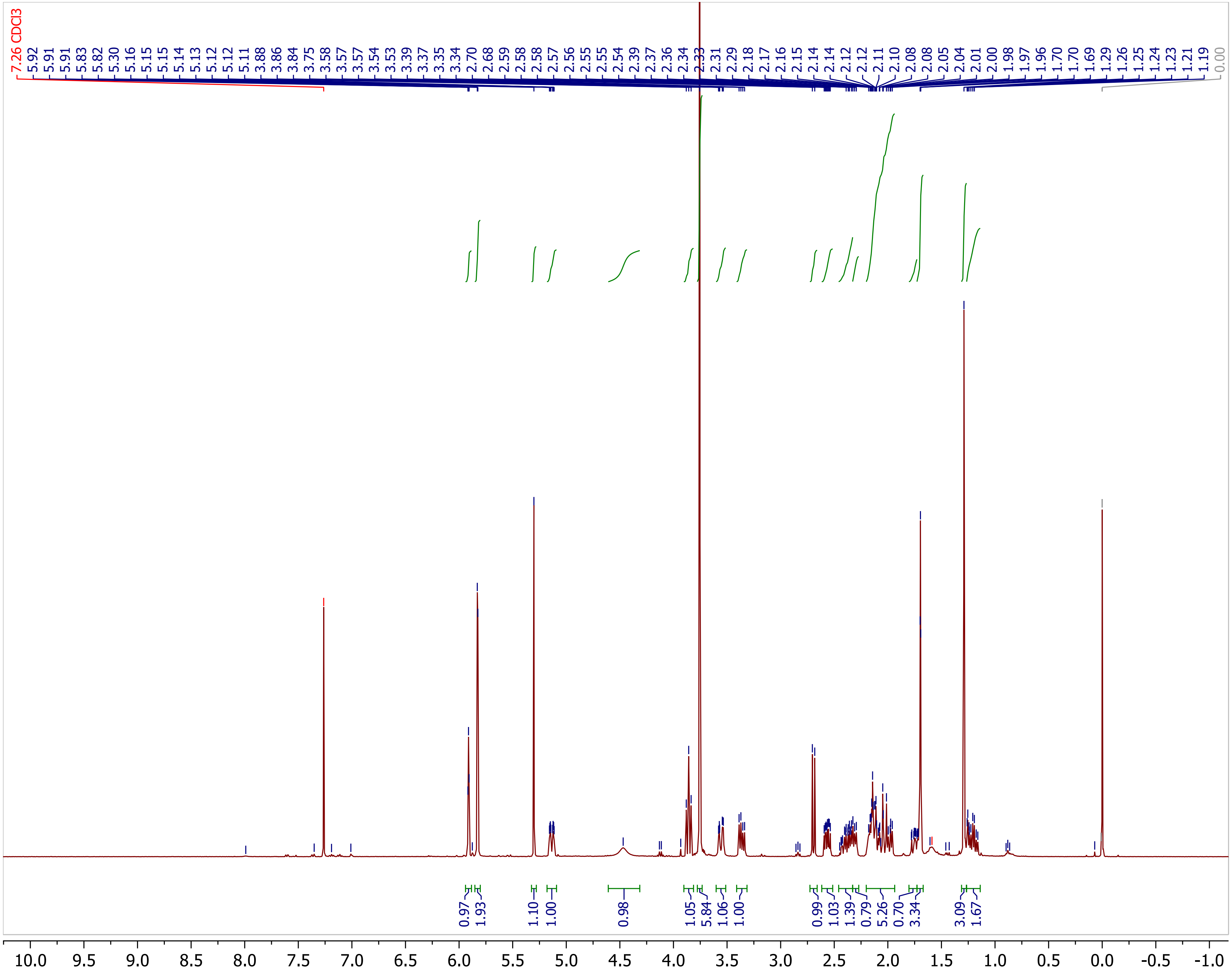

Current Data Parameters
NAME AQ-54
EXPNO 10
PROCNO 1

F2 - Acquisition Parameters
Date_ 20190315
Time 18.29
INSTRUM spect
PROBHD 5 mm PADUL 13C
PULPROG zg30
TD 32768
SOLVENT CDCl3
NS 32
DS 2
SWH 8223.685 Hz
FIDRES 0.250967 Hz
AQ 1.9922944 sec
RG 362
DW 60.800 usec
DE 16.65 usec
TE 294.4 K
D1 1.50000000 sec
TD0 1

======== CHANNEL f1 ========
SFO1 400.1324008 MHz
NUC1 1H
P1 11.06 usec
PLW1 24.29199982 W

F2 - Processing parameters
SI 32768
SF 400.1300086 MHz
WDW EM
SSB 0
LB 0.30 Hz
GB 0
PC 1.00

^{1}H NMR Spectrum **3h**

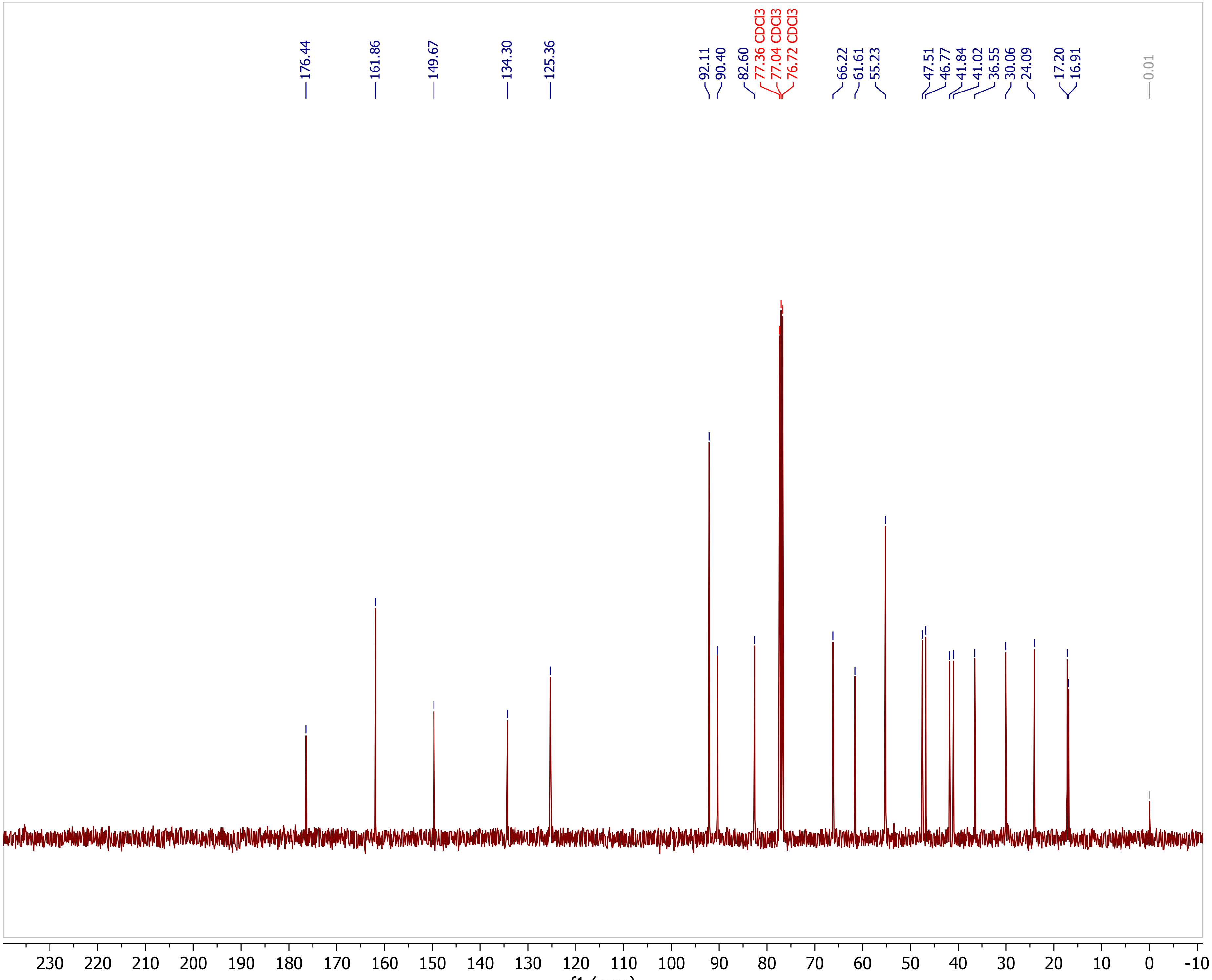

^{13}C NMR Spectrum **3h**

Current Data Parameters
NAME AQ-54
EXPNO 11
PROCNO 1

F2 - Acquisition Parameters
Date_ 20190315
Time 18.01
INSTRUM spect
PROBHD 5 mm PADUL 13C
PULPROG udeft
TD 18178
SOLVENT CDCl3
NS 380
DS 0
SWH 25252.525 Hz
FIDRES 1.389181 Hz
AQ 0.3599244 sec
RG 2050
DW 19.800 usec
DE 8.20 usec
TE 294.7 K
D1 3.00000000 sec
D11 0.03000000 sec
D12 0.00002000 sec
D20 200.00000000 sec
TD0 380

======== CHANNEL f1 ======
SFO1 100.6242690 MHz
NUC1 13C
P1 8.80 usec
P13 2000.00 usec
P26 500.00 usec
PLW1 58.63899994 W
SPNAM[5] Crp60comp.4
SPOAL5 0.500
SPOFFS5 0 Hz
SPW5 6.93809986 W
SPNAM[8] Crp60,0.5,20.1
SPOAL8 0.500
SPOFFS8 0 Hz
SPW8 6.93809986 W

======== CHANNEL f2 ======
SFO2 400.1320000 MHz
NUC2 1H
CPDPRG[2 waltz16
PCPD2 90.00 usec
PLW2 24.29199982 W
PLW12 0.28218001 W

F2 - Processing parameters
SI 65536
SF 100.6127690 MHz
WDW EM
SSB 0
LB 2.00 Hz
GB 0
PC 1.00

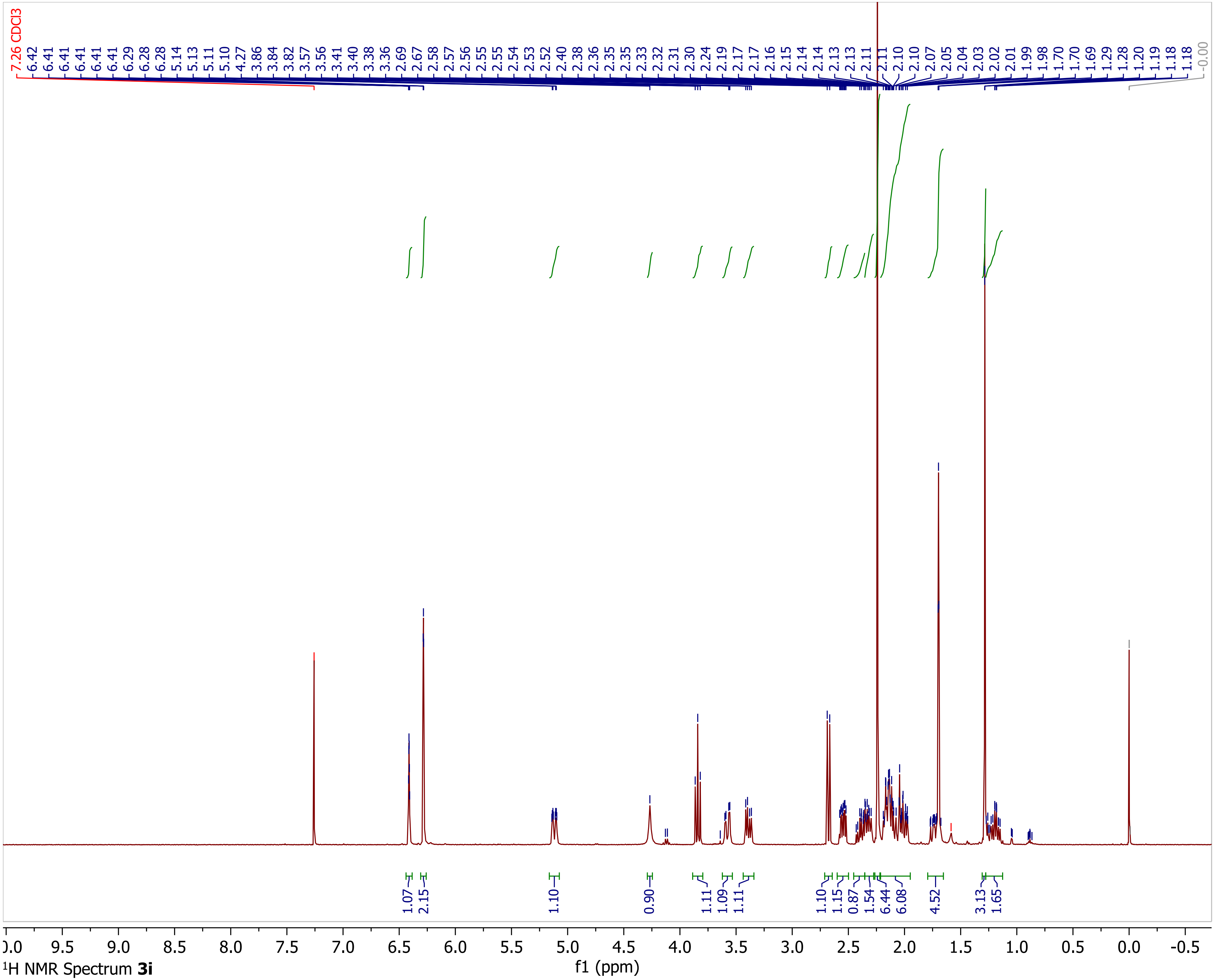

Current Data Parameters
NAME AQ-55x
EXPNO 10
PROCNO 1

F2 - Acquisition Parameters
Date_ 20190313
Time 12.35 h
INSTRUM AvanceNeo, Otter
PROBHD Z116098_0793 (
PULPROG zg30
TD 65536
SOLVENT CDCl3
NS 2
DS 0
SWH 7142.857 Hz
FIDRES 0.217983 Hz
AQ 4.5875201 sec
RG 101
DW 70.000 usec
DE 7.04 usec
TE 298.0 K
D1 2.00000000 sec
TD0 1
SFO1 400.1324008 MHz
NUC1 1H
P0 3.33 usec
P1 10.00 usec
PLW1 18.69700050 W

F2 - Processing parameters
SI 131072
SF 400.1300104 MHz
WDW EM
SSB 0
LB 0.10 Hz
GB 0
PC 1.00

^{1}H NMR Spectrum **3i**

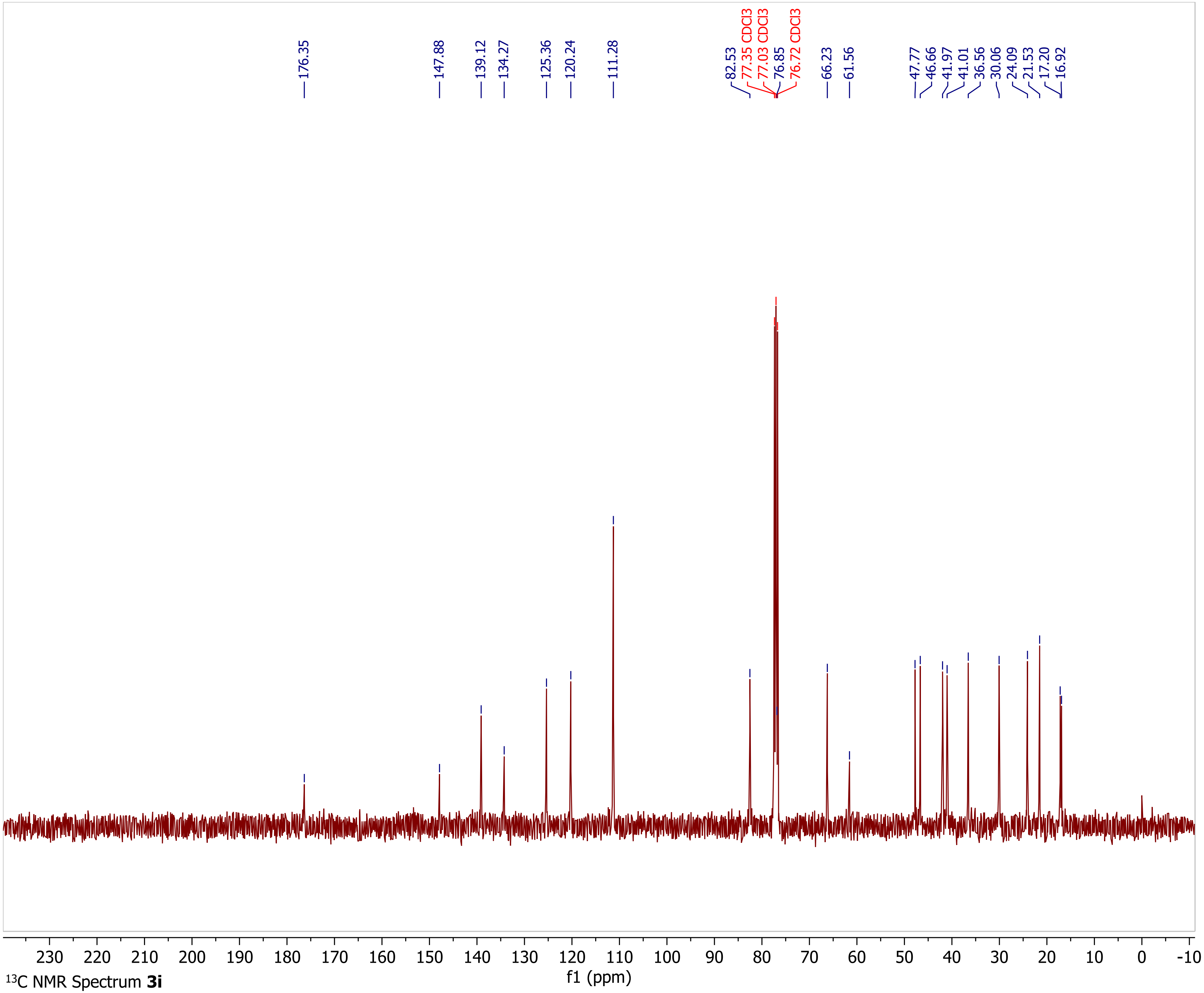

^{13}C NMR Spectrum **3i**

Current Data Parameters
NAME AQ-55 X2
EXPNO 11
PROCNO 1

F2 - Acquisition Parameters
Date_ 20190405
Time 1.11
INSTRUM spect
PROBHD 5 mm PADUL 13C
PULPROG udeft
TD 18178
SOLVENT CDCl3
NS 380
DS 0
SWH 25252.525 Hz
FIDRES 1.389181 Hz
AQ 0.3599244 sec
RG 2050
DW 19.800 usec
DE 8.20 usec
TE 294.7 K
D1 3.00000000 sec
D11 0.03000000 sec
D12 0.00002000 sec
D20 200.00000000 sec
TD0 380

======== CHANNEL f1 =======
SFO1 100.6242690 MHz
NUC1 13C
P1 8.80 usec
P13 2000.00 usec
P26 500.00 usec
PLW1 58.63899994 W
SPNAM[5] Crp60comp.4
SPOAL5 0.500
SPOFFS5 0 Hz
SPW5 6.93809986 W
SPNAM[8] Crp60,0.5,20.1
SPOAL8 0.500
SPOFFS8 0 Hz
SPW8 6.93809986 W

======== CHANNEL f2 =======
SFO2 400.1320000 MHz
NUC2 1H
CPDPRG[2 waltz16
PCPD2 90.00 usec
PLW2 24.29199982 W
PLW12 0.28218001 W

F2 - Processing parameters
SI 65536
SF 100.6127690 MHz
WDW EM
[illegible]

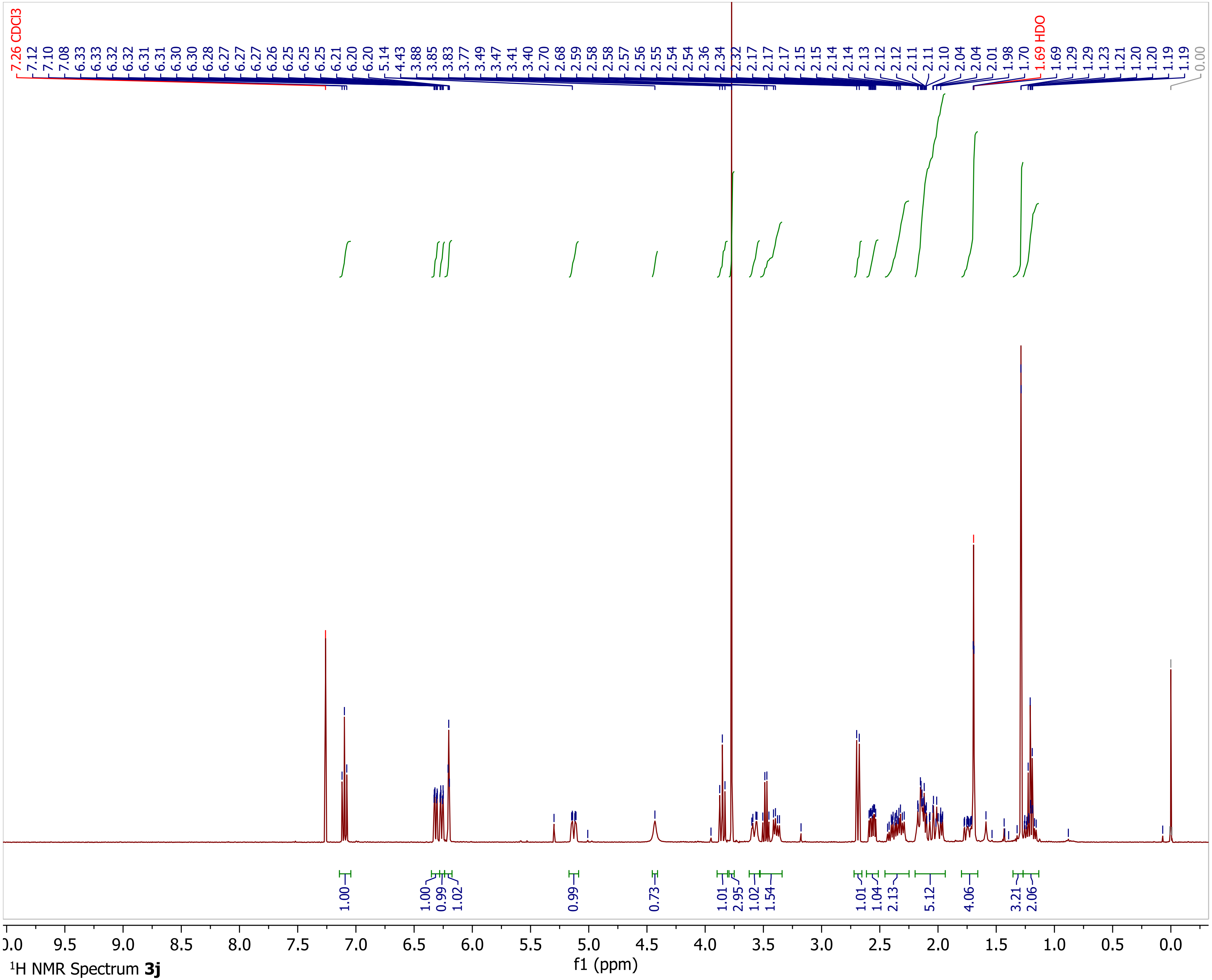

Current Data Parameters
NAME AQ-81
EXPNO 10
PROCNO 1

F2 - Acquisition Parameters
Date_ 20190416
Time 9.36 h
INSTRUM AvanceNeo
PROBHD Z116098_0793 (
PULPROG zg30
TD 65536
SOLVENT CDCl3
NS 2
DS 0
SWH 7142.857 Hz
FIDRES 0.217983 Hz
AQ 4.5875201 sec
RG 101
DW 70.000 usec
DE 14.62 usec
TE 298.0 K
D1 2.00000000 sec
TD0 1
SFO1 400.1324008 MHz
NUC1 1H
P0 3.33 usec
P1 10.00 usec
PLW1 18.69700050 W

F2 - Processing parameters
SI 131072
SF 400.1300091 MHz
WDW EM
SSB 0
LB 0.10 Hz
GB 0
PC 1.00

^{1}H NMR Spectrum **3j**

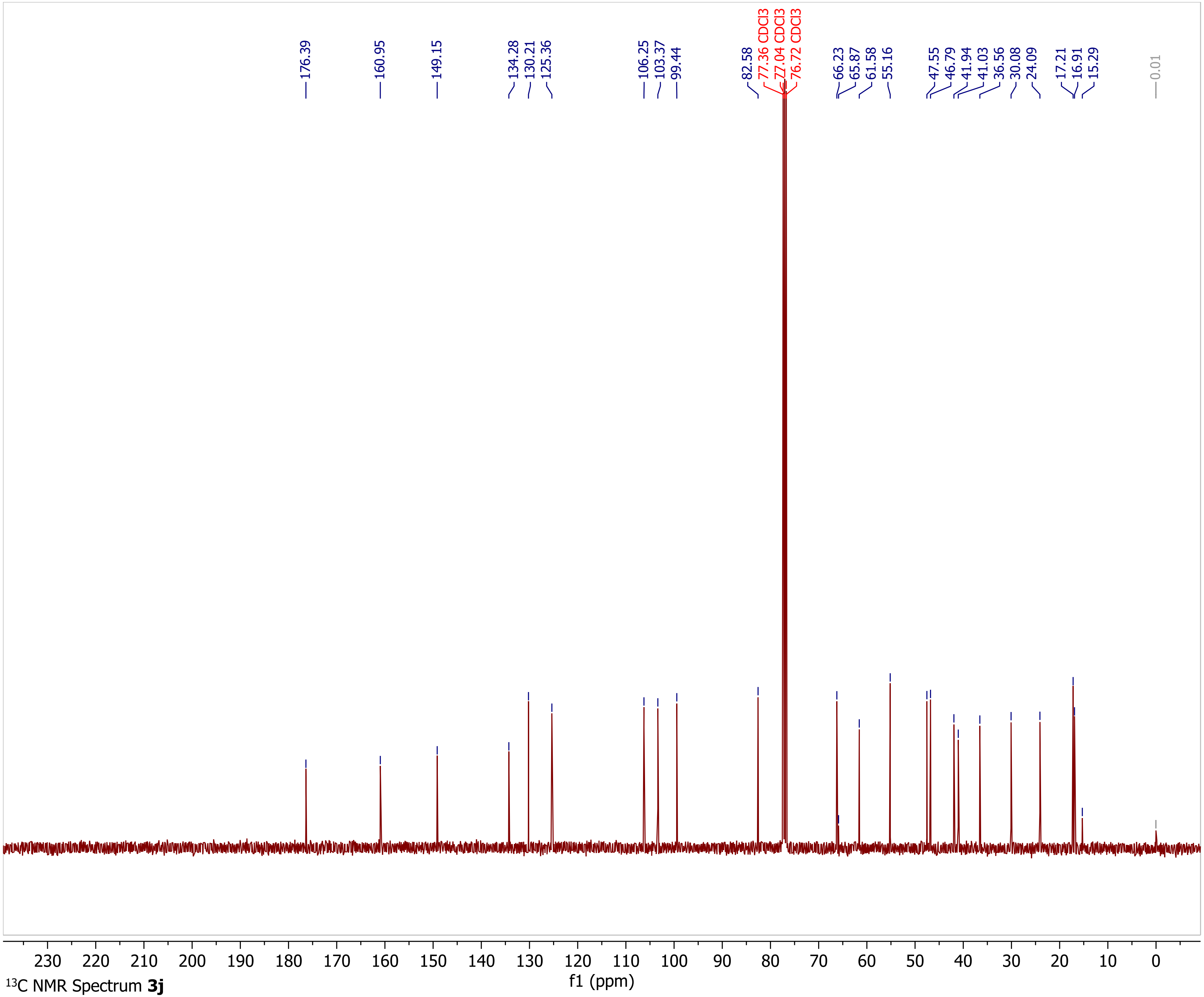

Current Data Parameters
NAME AQ-81
EXPNO 11
PROCNO 1

F2 - Acquisition Parameters
Date_ 20190416
Time 18.08 h
INSTRUM AvanceNeo
PROBHD Z116098_0793 (
PULPROG zgpg30
TD 119044
SOLVENT CDCl3
NS 512
DS 0
SWH 25000.000 Hz
FIDRES 0.420013 Hz
AQ 2.3808801 sec
RG 31.9602
DW 20.000 usec
DE 7.12 usec
TE 298.0 K
D1 1.00000000 sec
D11 0.03000000 sec
TD0 1
SFO1 100.6243390 MHz
NUC1 13C
P0 3.33 usec
P1 10.00 usec
PLW1 83.92700195 W
SFO2 400.1318006 MHz
NUC2 1H
CPDPRG[2 waltz64
PCPD2 90.00 usec
PLW2 18.69700050 W
PLW12 0.23083000 W
PLW13 0.11611000 W

F2 - Processing parameters
SI 131072
SF 100.6127685 MHz
WDW EM
SSB 0
LB 1.00 Hz
GB 0
PC 1.40

^{13}C NMR Spectrum **3j**

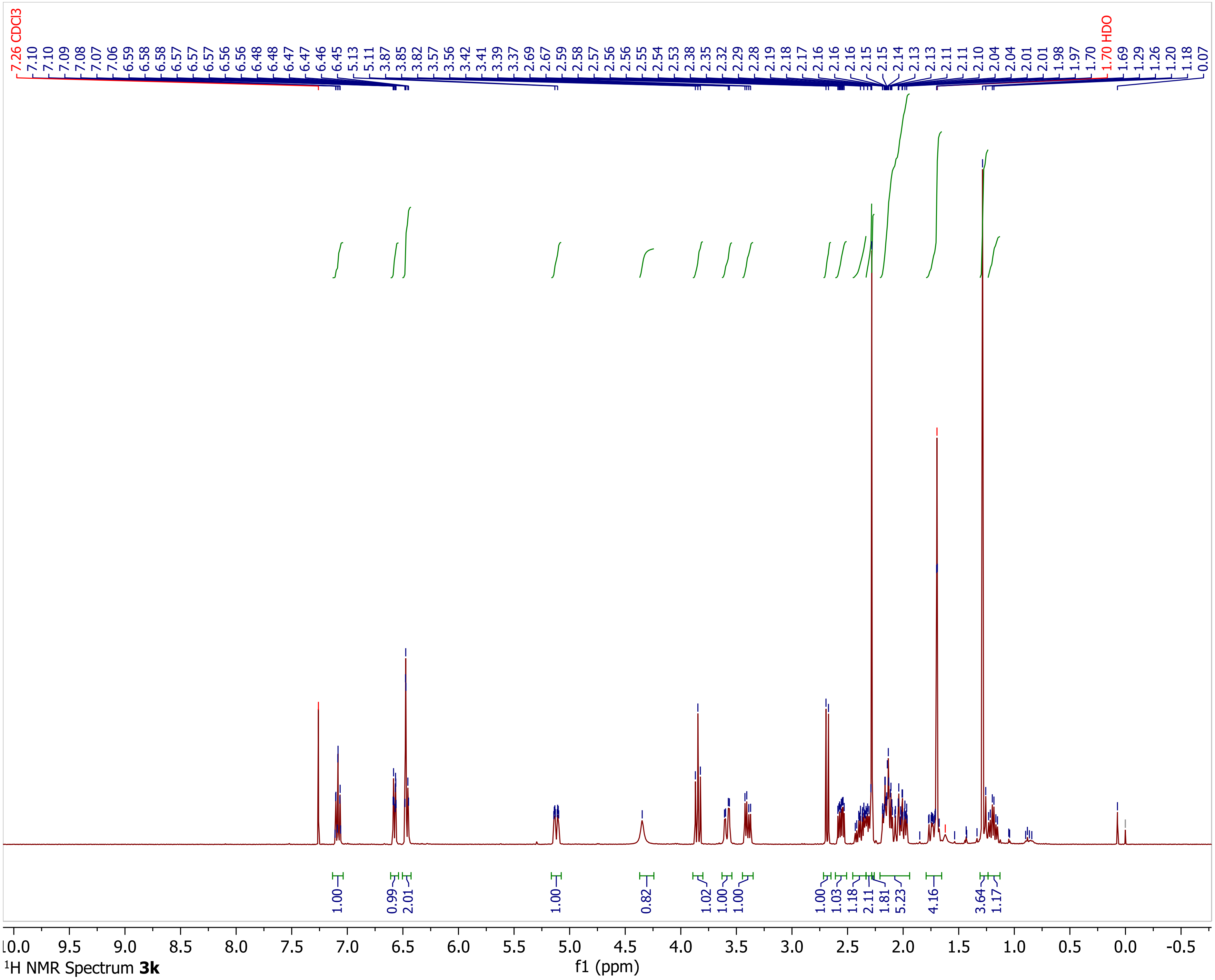

Current Data Parameters
NAME AQ-90
EXPNO 10
PROCNO 1

F2 - Acquisition Parameters
Date_ 20190508
Time 14.01 h
INSTRUM AvanceNeo
PROBHD Z116098_0793 (
PULPROG zg30
TD 65536
SOLVENT CDCl3
NS 2
DS 0
SWH 7142.857 Hz
FIDRES 0.217983 Hz
AQ 4.5875201 sec
RG 75
DW 70.000 usec
DE 14.62 usec
TE 298.0 K
D1 2.00000000 sec
TD0 1
SFO1 400.1324008 MHz
NUC1 1H
P0 3.33 usec
P1 10.00 usec
PLW1 18.69700050 W

F2 - Processing parameters
SI 131072
SF 400.1300384 MHz
WDW EM
SSB 0
LB 0.10 Hz
GB 0
PC 1.00

^{1}H NMR Spectrum **3k**

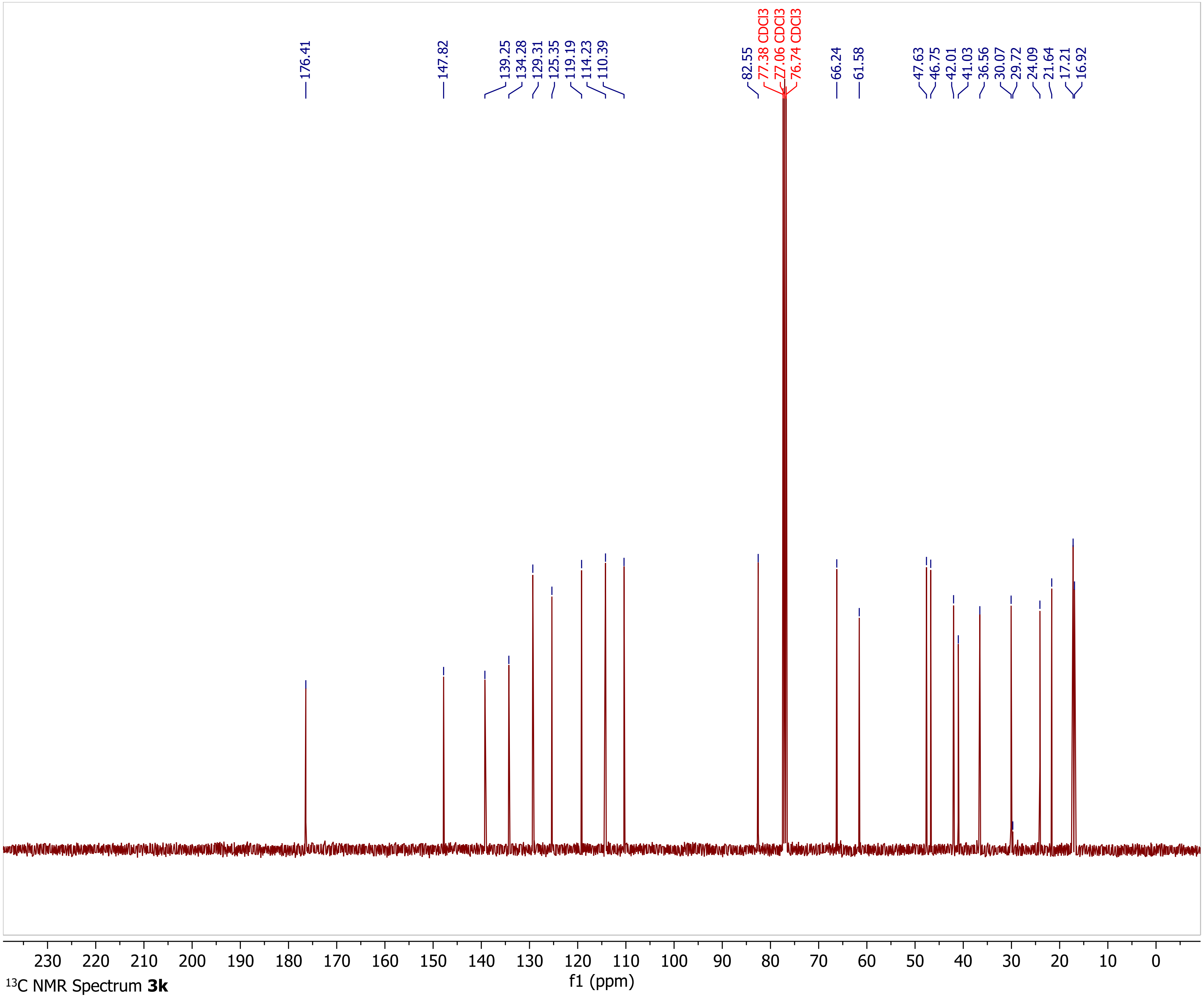

Current Data Parameters
NAME AQ-90
EXPNO 11
PROCNO 1

F2 - Acquisition Parameters
Date_ 20190508
Time 22.27 h
INSTRUM AvanceNeo
PROBHD Z116098_0793 (
PULPROG zgpg30
TD 119044
SOLVENT CDCl3
NS 512
DS 0
SWH 25000.000 Hz
FIDRES 0.420013 Hz
AQ 2.3808801 sec
RG 35.1563
DW 20.000 usec
DE 7.12 usec
TE 298.0 K
D1 1.00000000 sec
D11 0.03000000 sec
TD0 1
SFO1 100.6243390 MHz
NUC1 13C
P0 3.33 usec
P1 10.00 usec
PLW1 83.92700195 W
SFO2 400.1318006 MHz
NUC2 1H
CPDPRG[2 waltz64
PCPD2 90.00 usec
PLW2 18.69700050 W
PLW12 0.23083000 W
PLW13 0.11611000 W

F2 - Processing parameters
SI 131072
SF 100.6127685 MHz
WDW EM
SSB 0
LB 1.00 Hz
GB 0
PC 1.40

^{13}C NMR Spectrum **3k**

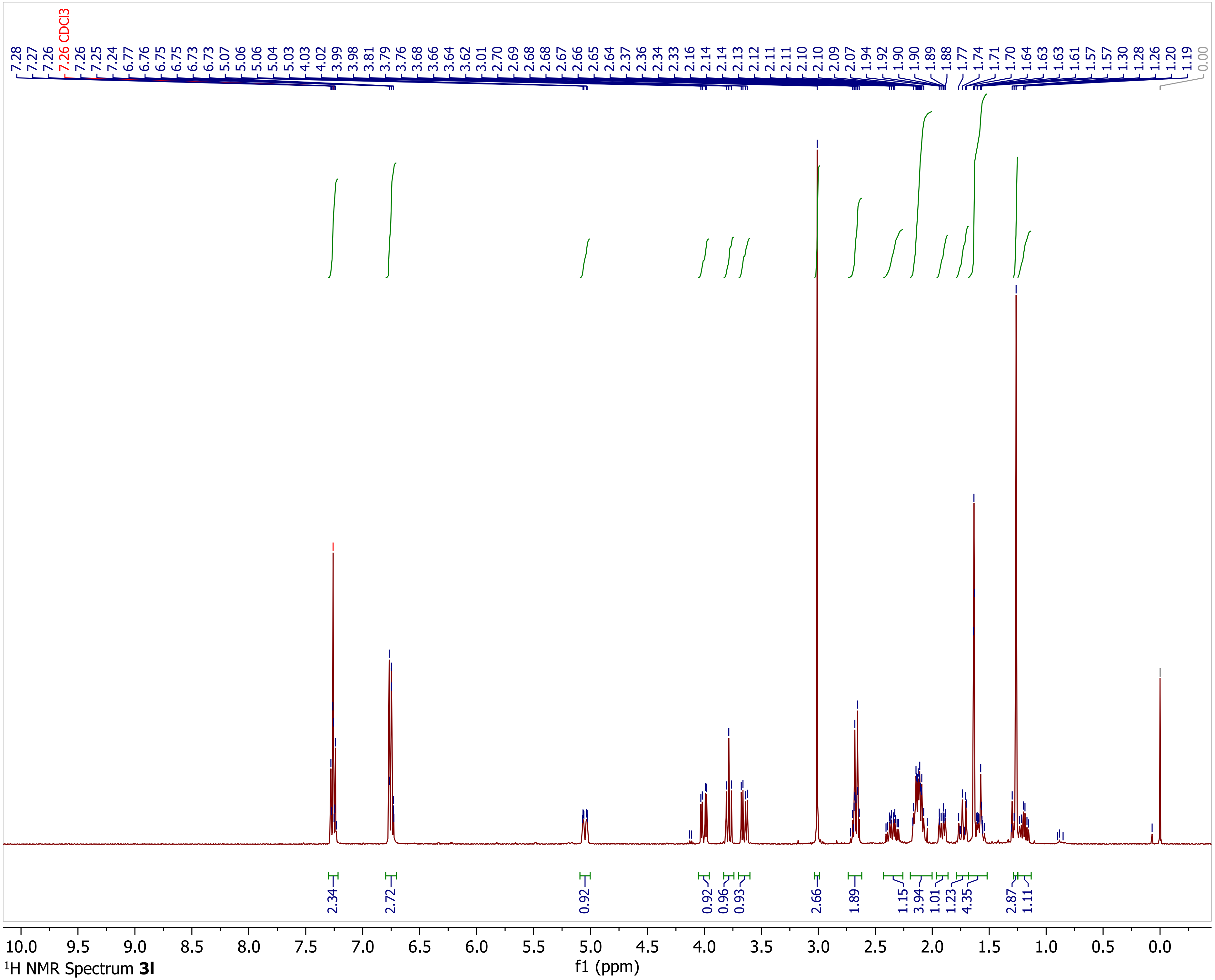

Current Data Parameters
NAME AQ-56 X
EXPNO 10
PROCNO 1

F2 - Acquisition Parameters
Date_ 20190409
Time 6.12 h
INSTRUM AvanceNeo
PROBHD Z116098_0793 (
PULPROG zg30
TD 65536
SOLVENT CDCl3
NS 2
DS 0
SWH 7142.857 Hz
FIDRES 0.217983 Hz
AQ 4.5875201 sec
RG 101
DW 70.000 usec
DE 14.62 usec
TE 298.0 K
D1 2.00000000 sec
TD0 1
SFO1 400.1324008 MHz
NUC1 1H
P0 3.33 usec
P1 10.00 usec
PLW1 18.69700050 W

F2 - Processing parameters
SI 131072
SF 400.1300095 MHz
WDW EM
SSB 0
LB 0.10 Hz
GB 0
PC 1.00

^{1}H NMR Spectrum **3l**

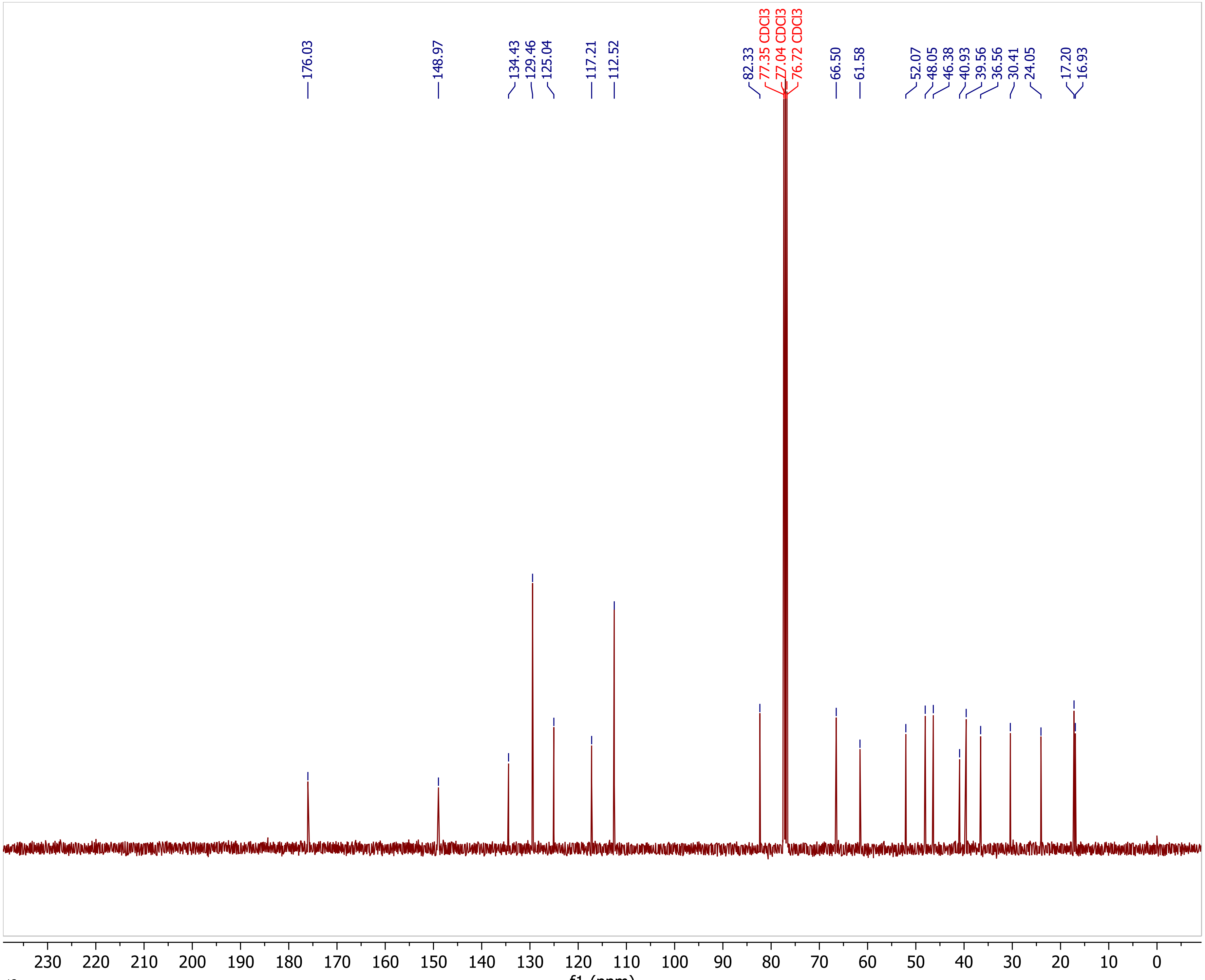

Current Data Parameters
NAME AQ-56 X
EXPNO 11
PROCNO 1

F2 - Acquisition Parameters
Date_ 20190409
Time 6.42 h
INSTRUM AvanceNeo
PROBHD Z116098_0793 (
PULPROG zgpg30
TD 119044
SOLVENT CDCl3
NS 512
DS 0
SWH 25000.000 Hz
FIDRES 0.420013 Hz
AQ 2.3808801 sec
RG 35.1563
DW 20.000 usec
DE 7.12 usec
TE 298.0 K
D1 1.00000000 sec
D11 0.03000000 sec
TD0 1
SFO1 100.6243390 MHz
NUC1 13C
P0 3.33 usec
P1 10.00 usec
PLW1 83.92700195 W
SFO2 400.1318006 MHz
NUC2 1H
CPDPRG[2 waltz64
PCPD2 90.00 usec
PLW2 18.69700050 W
PLW12 0.23083000 W
PLW13 0.11611000 W

F2 - Processing parameters
SI 131072
SF 100.6127685 MHz
WDW EM
SSB 0
LB 1.00 Hz
GB 0
PC 1.40

^{13}C NMR Spectrum **3l**

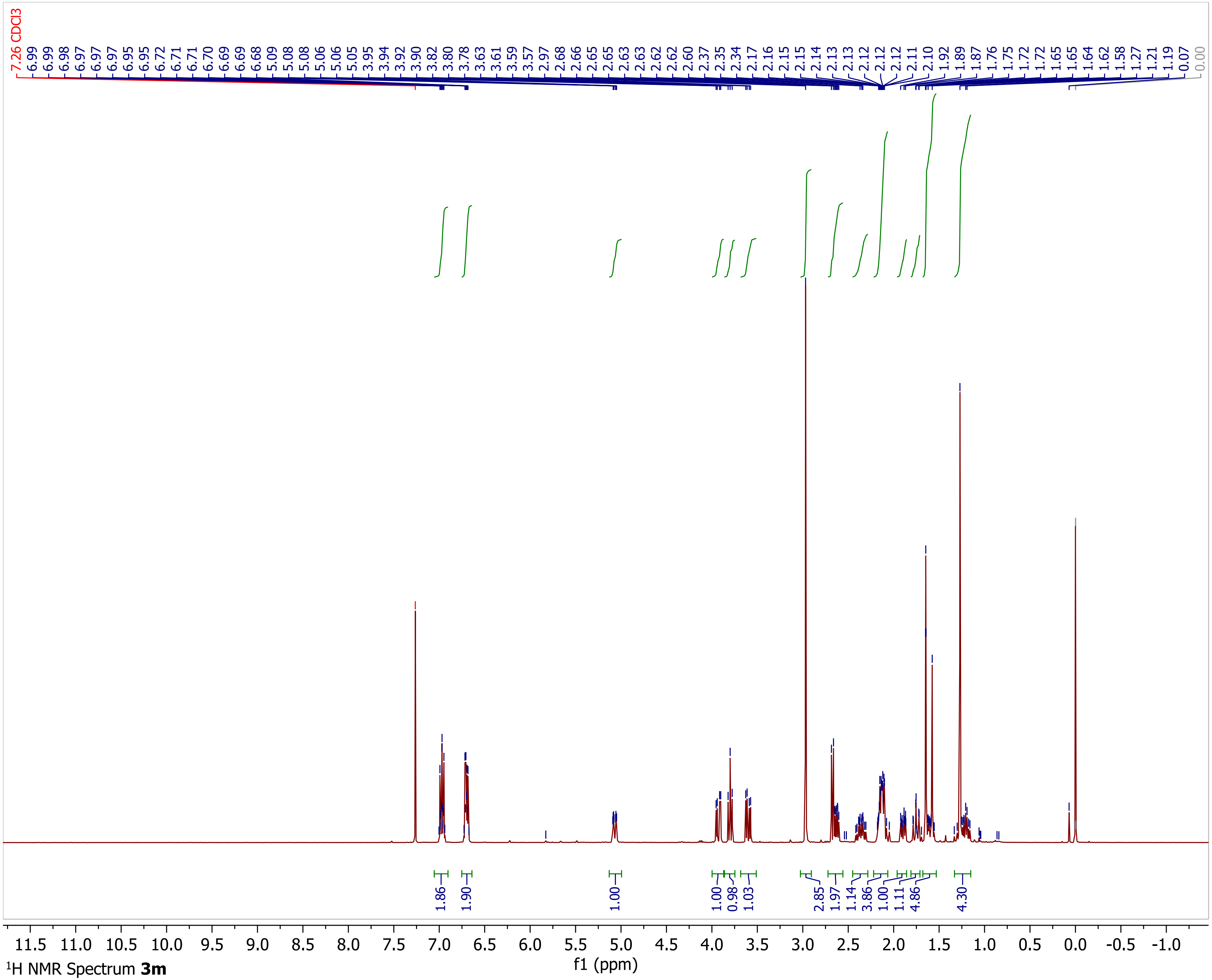

Current Data Parameters
NAME AQ-100-2x
EXPNO 10
PROCNO 1

F2 - Acquisition Parameters
Date_ 20190617
Time 14.23
INSTRUM spect
PROBHD 5 mm PADUL 13C
PULPROG zg30
TD 32768
SOLVENT CDCl3
NS 32
DS 2
SWH 8223.685 Hz
FIDRES 0.250967 Hz
AQ 1.9922944 sec
RG 456
DW 60.800 usec
DE 16.65 usec
TE 294.3 K
D1 1.50000000 sec
TD0 1

======== CHANNEL f1 ========
SFO1 400.1324008 MHz
NUC1 1H
P1 11.06 usec
PLW1 24.29199982 W

F2 - Processing parameters
SI 32768
SF 400.1300086 MHz
WDW EM
SSB 0
LB 0.30 Hz
GB 0
PC 1.00

^{1}H NMR Spectrum **3m**

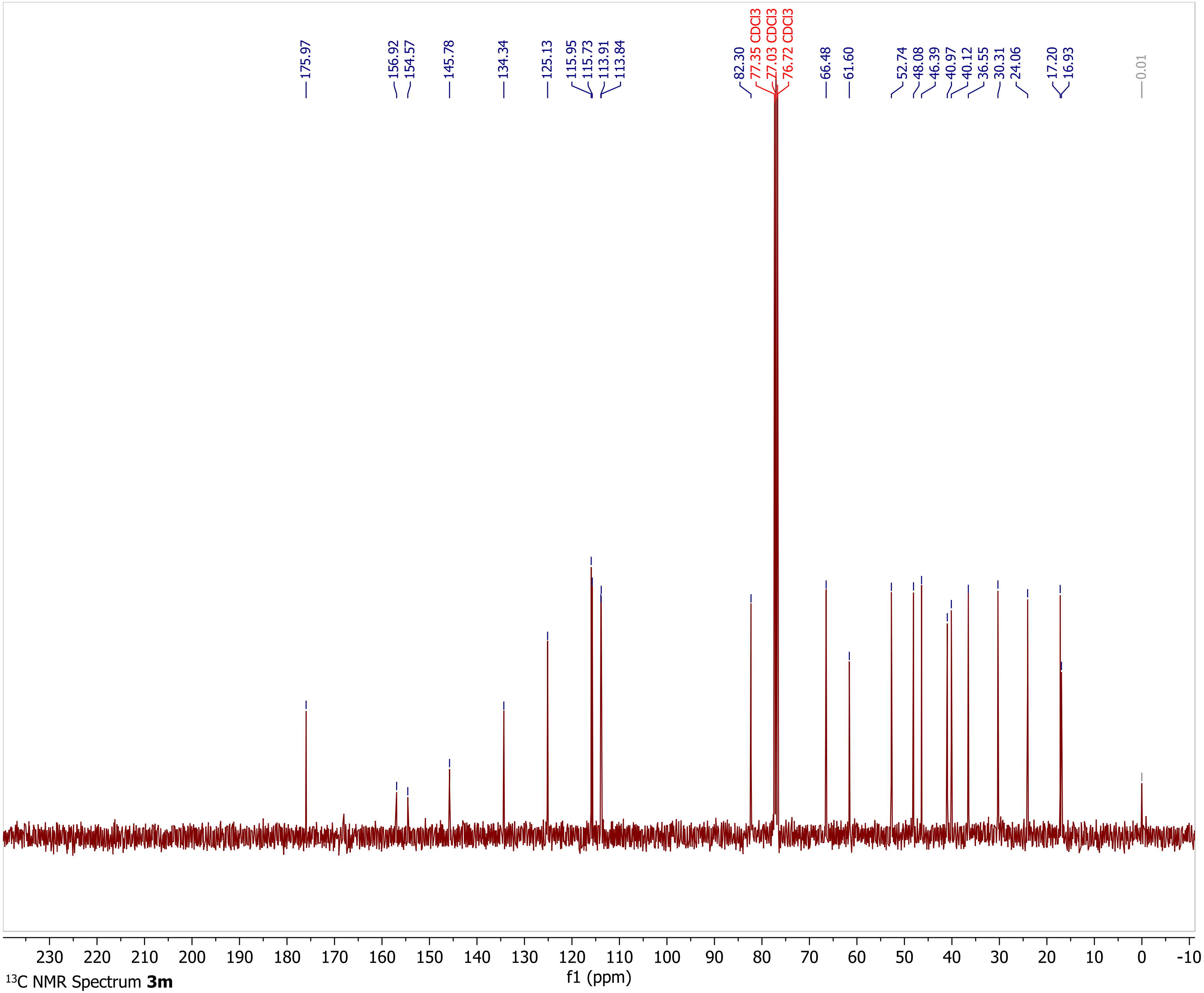

Current Data Parameters
NAME AQ-100-2x
EXPNO 12
PROCNO 1

F2 - Acquisition Parameters
Date_ 20190617
Time 14.33
INSTRUM spect
PROBHD 5 mm PADUL 13C
PULPROG udeft
TD 18178
SOLVENT CDCl3
NS 380
DS 0
SWH 25252.525 Hz
FIDRES 1.389181 Hz
AQ 0.3599244 sec
RG 2050
DW 19.800 usec
DE 8.20 usec
TE 294.9 K
D1 3.00000000 sec
D11 0.03000000 sec
D12 0.00002000 sec
D20 200.00000000 sec
TD0 380

======== CHANNEL f1 ========
SFO1 100.6242690 MHz
NUC1 13C
P1 8.80 usec
P13 2000.00 usec
P26 500.00 usec
PLW1 58.63899994 W
SPNAM[5] Crp60comp.4
SPOAL5 0.500
SPOFFS5 0 Hz
SPW5 6.93809986 W

^{13}C NMR Spectrum **3m**

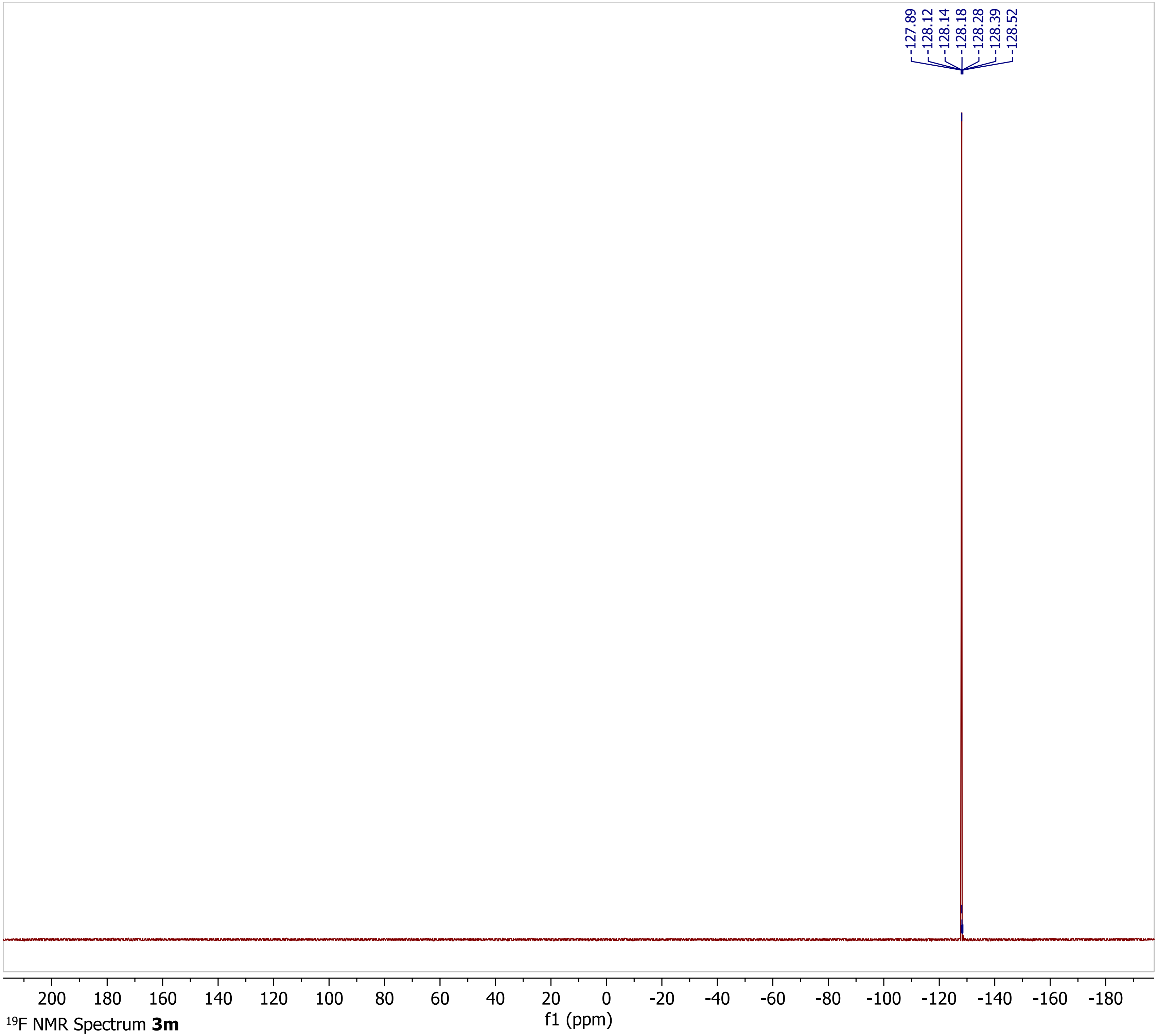

Current Data Parameters
NAME AQ-100-2
EXPNO 11
PROCNO 1

F2 - Acquisition Parameters
Date_ 20190617
Time 14.07 h
INSTRUM AvanceNeo
PROBHD Z116098_0793 (
PULPROG zgig30
TD 261948
SOLVENT CDCl3
NS 16
DS 0
SWH 156250.000 Hz
FIDRES 1.192985 Hz
AQ 0.8382336 sec
RG 101
DW 3.200 usec
DE 6.82 usec
TE 298.0 K
D1 2.00000000 sec
D11 0.03000000 sec
TD0 1
SFO1 376.5021312 MHz
NUC1 19F
P0 6.00 usec
P1 18.00 usec
PLW1 18.94099998 W
SFO2 400.1318006 MHz
NUC2 1H
CPDPRG[2 waltz16
PCPD2 90.00 usec
PLW2 18.69700050 W
PLW12 0.23083000 W

F2 - Processing parameters
SI 262144
SF 376.4983662 MHz
WDW EM
SSB 0
LB 1.00 Hz
GB 0
PC 2.00

^{19}F NMR Spectrum **3m**

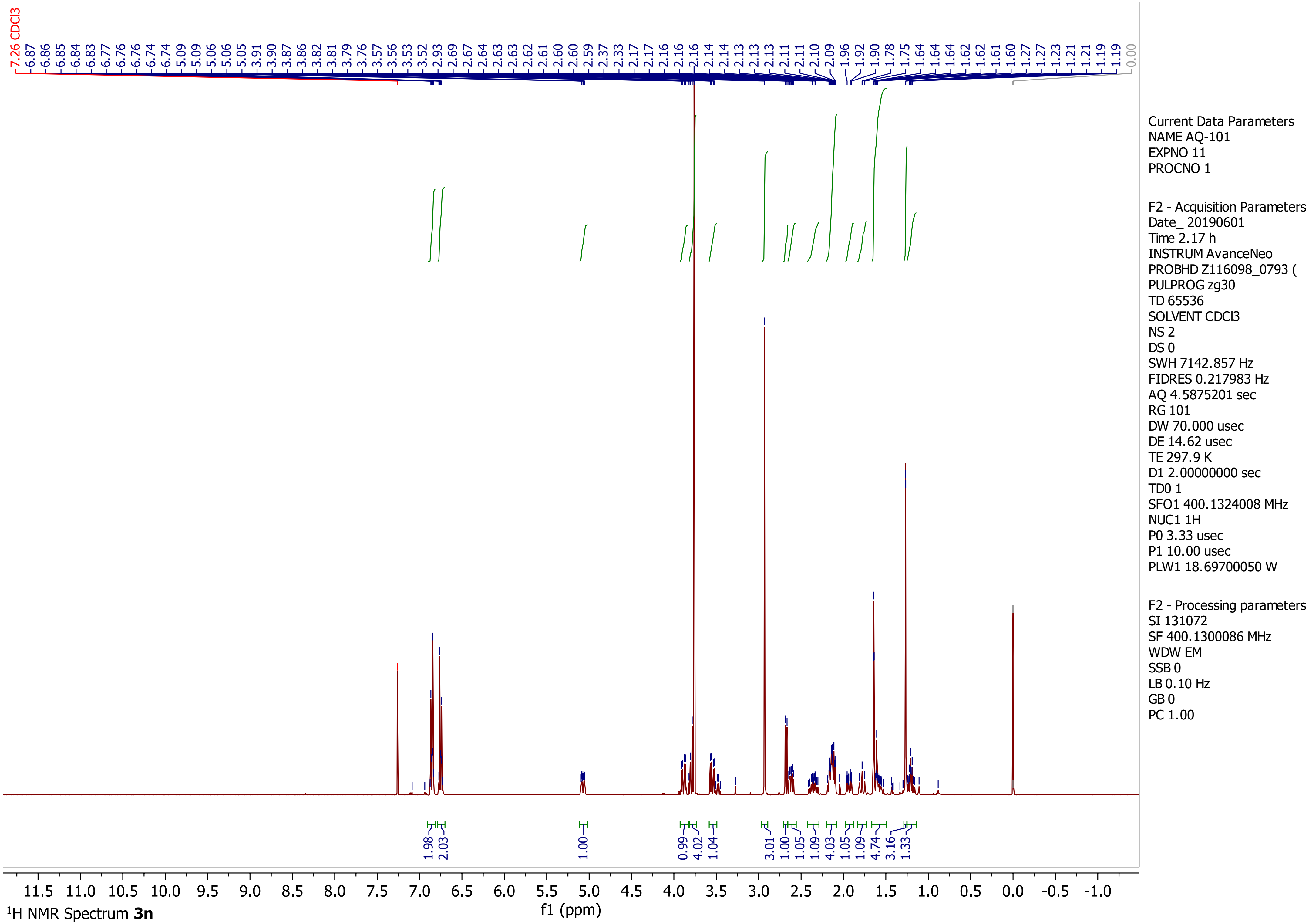

^{1}H NMR Spectrum **3n**

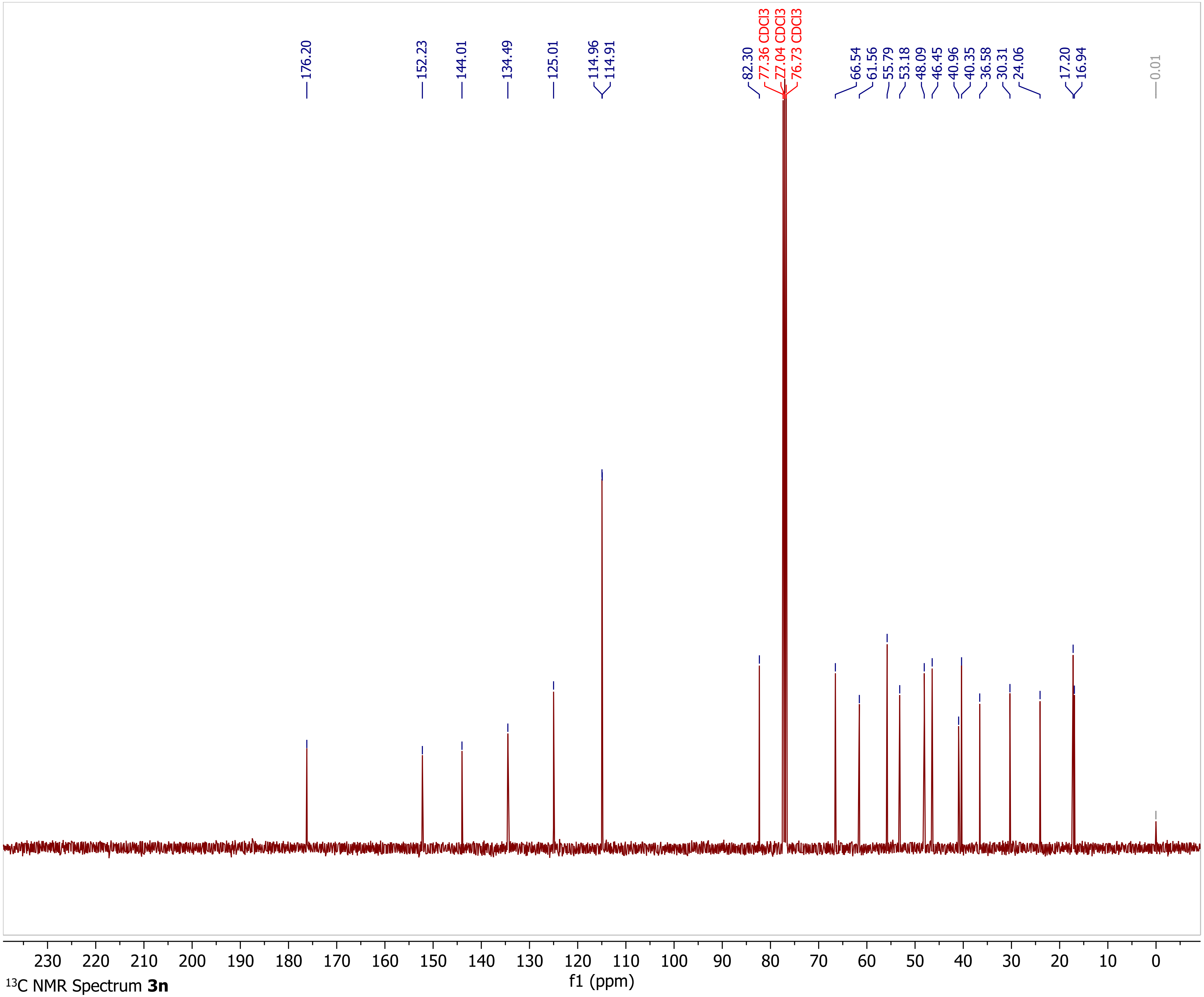

Current Data Parameters
NAME AQ-101
EXPNO 10
PROCNO 1

F2 - Acquisition Parameters
Date_ 20190601
Time 2.16 h
INSTRUM AvanceNeo
PROBHD Z116098_0793 (
PULPROG zgpg30
TD 119044
SOLVENT CDCl3
NS 512
DS 0
SWH 25000.000 Hz
FIDRES 0.420013 Hz
AQ 2.3808801 sec
RG 31.9602
DW 20.000 usec
DE 7.12 usec
TE 298.0 K
D1 1.00000000 sec
D11 0.03000000 sec
TD0 1
SFO1 100.6243390 MHz
NUC1 13C
P0 3.33 usec
P1 10.00 usec
PLW1 83.92700195 W
SFO2 400.1318006 MHz
NUC2 1H
CPDPRG[2 waltz64
PCPD2 90.00 usec
PLW2 18.69700050 W
PLW12 0.23083000 W
PLW13 0.11611000 W

F2 - Processing parameters

^{13}C NMR Spectrum **3n**

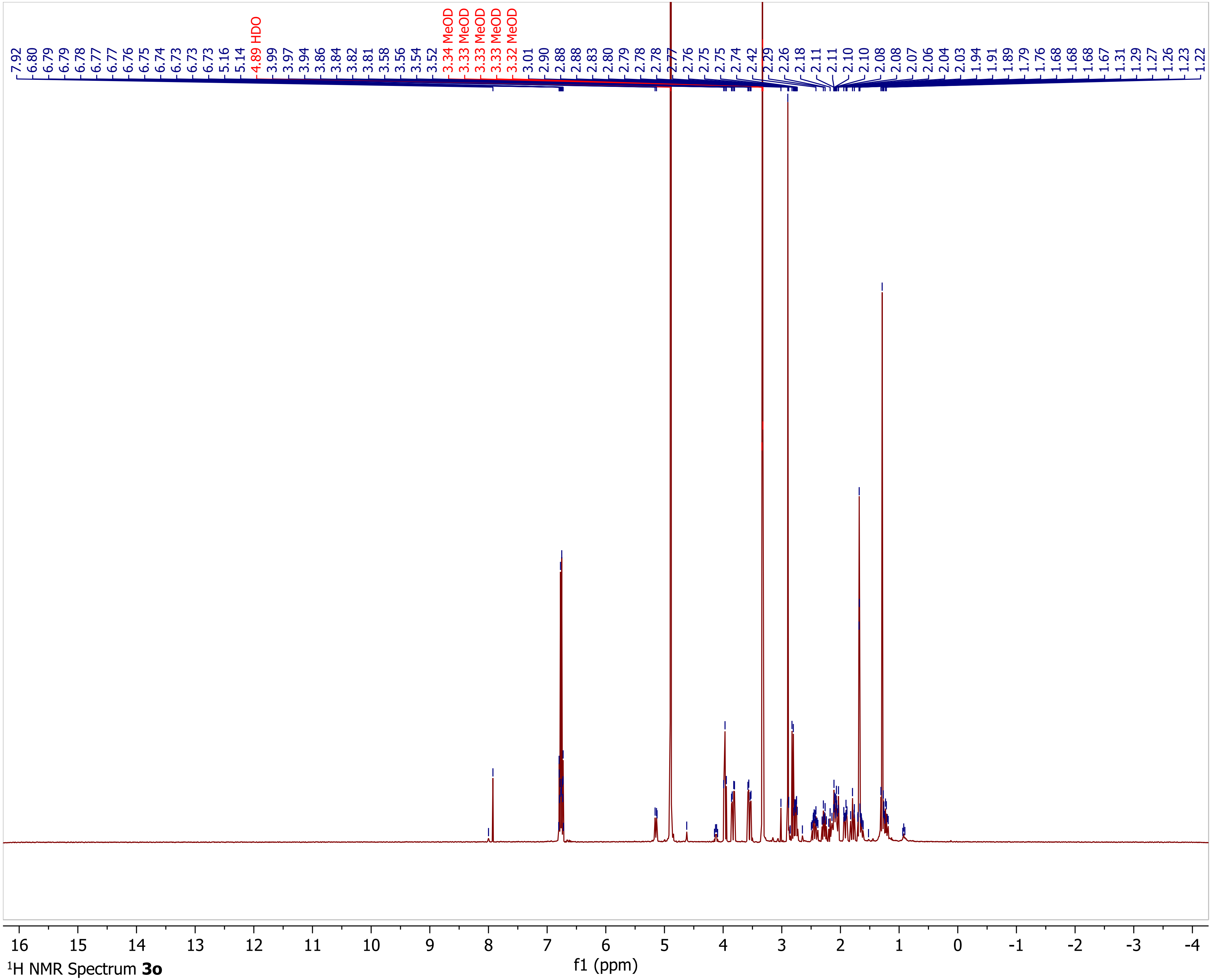

Current Data Parameters
NAME 2017-Oct-30-Fossey-27
EXPNO 10
PROCNO 1

F2 - Acquisition Parameters
Date_ 20171031
Time 5.02
INSTRUM spect
PROBHD 5 mm PADUL 13C
PULPROG zg30
TD 32768
SOLVENT MeOD
NS 32
DS 2
SWH 8223.685 Hz
FIDRES 0.250967 Hz
AQ 1.9922944 sec
RG 512
DW 60.800 usec
DE 16.65 usec
TE 295.5 K
D1 1.50000000 sec
TD0 1

======== CHANNEL f1 ========
SFO1 400.1324008 MHz
NUC1 1H
P1 11.06 usec
PLW1 24.29199982 W

F2 - Processing parameters
SI 32768
SF 400.1300000 MHz
WDW EM
SSB 0
LB 0.30 Hz
GB 0
PC 1.00

^{1}H NMR Spectrum **3o**

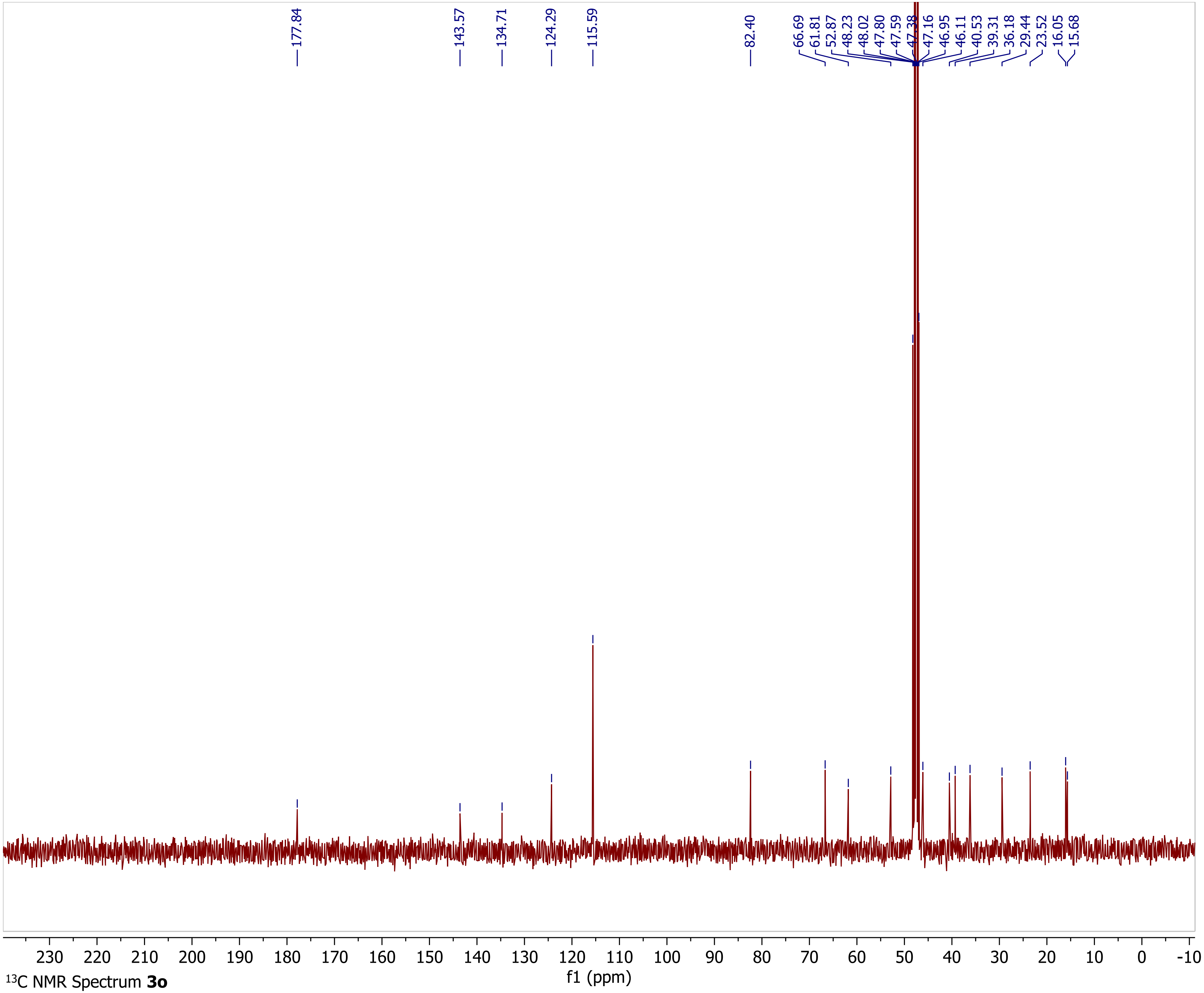

Current Data Parameters
NAME 2017-Oct-30-Fossey-27
EXPNO 12
PROCNO 1

F2 - Acquisition Parameters
Date_ 20171031
Time 5.39
INSTRUM spect
PROBHD 5 mm PADUL 13C
PULPROG udeft
TD 18178
SOLVENT MeOD
NS 380
DS 0
SWH 25252.525 Hz
FIDRES 1.389181 Hz
AQ 0.3599244 sec
RG 2050
DW 19.800 usec
DE 8.20 usec
TE 295.5 K
D1 3.00000000 sec
D11 0.03000000 sec
D12 0.00002000 sec
D20 200.00000000 sec
TD0 380

======== CHANNEL f1 ========
SFO1 100.6242690 MHz
NUC1 13C
P1 8.80 usec
P13 2000.00 usec
P26 500.00 usec
PLW1 58.63899994 W
SPNAM[5] Crp60comp.4
SPOAL5 0.500
SPOFFS5 0 Hz
SPW5 6.93809986 W
SPNAM[8] Crp60,0.5,20.1
SPOAL8 0.500
SPOFFS8 0 Hz
SPW8 6.93809986 W

======== CHANNEL f2 ========
SFO2 400.1320000 MHz
NUC2 1H
CPDPRG[2 waltz16
PCPD2 90.00 usec
PLW2 24.29199982 W
PLW12 0.28218001 W

F2 - Processing parameters
SI 65536
SF 100.6127690 MHz
WDW EM
SSB 0
LB 2.00 Hz
GB 0
PC 1.00

^{13}C NMR Spectrum **3o**

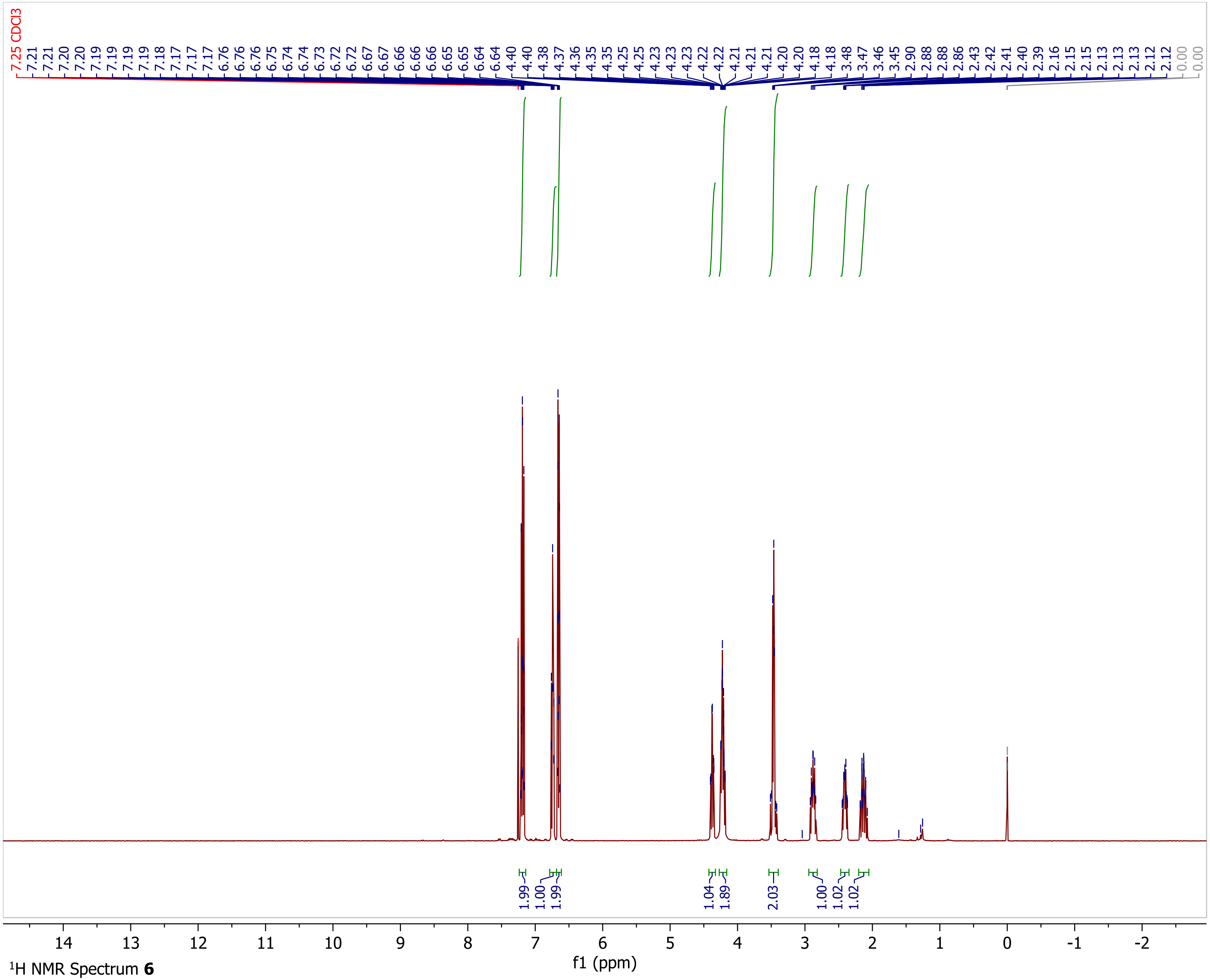

Current Data Parameters
NAME AQ-XL-6
EXPNO 10
PROCNO 1

F2 - Acquisition Parameters
Date_ 20200124
Time 13.41 h
INSTRUM AvanceNeo
PROBHD Z116098_0793 (
PULPROG zg30
TD 65536
SOLVENT CDCl3
NS 4
DS 0
SWH 7142.857 Hz
FIDRES 0.217983 Hz
AQ 4.5875201 sec
RG 92.3077
DW 70.000 usec
DE 14.80 usec
TE 298.0 K
D1 2.00000000 sec
TD0 1
SFO1 400.1324008 MHz
NUC1 1H
P0 3.13 usec
P1 9.40 usec
PLW1 18.69700050 W

F2 - Processing parameters
SI 131072
SF 400.1300125 MHz
WDW EM
SSB 0
LB 0.10 Hz
GB 0
PC 1.00

^{1}H NMR Spectrum **6**

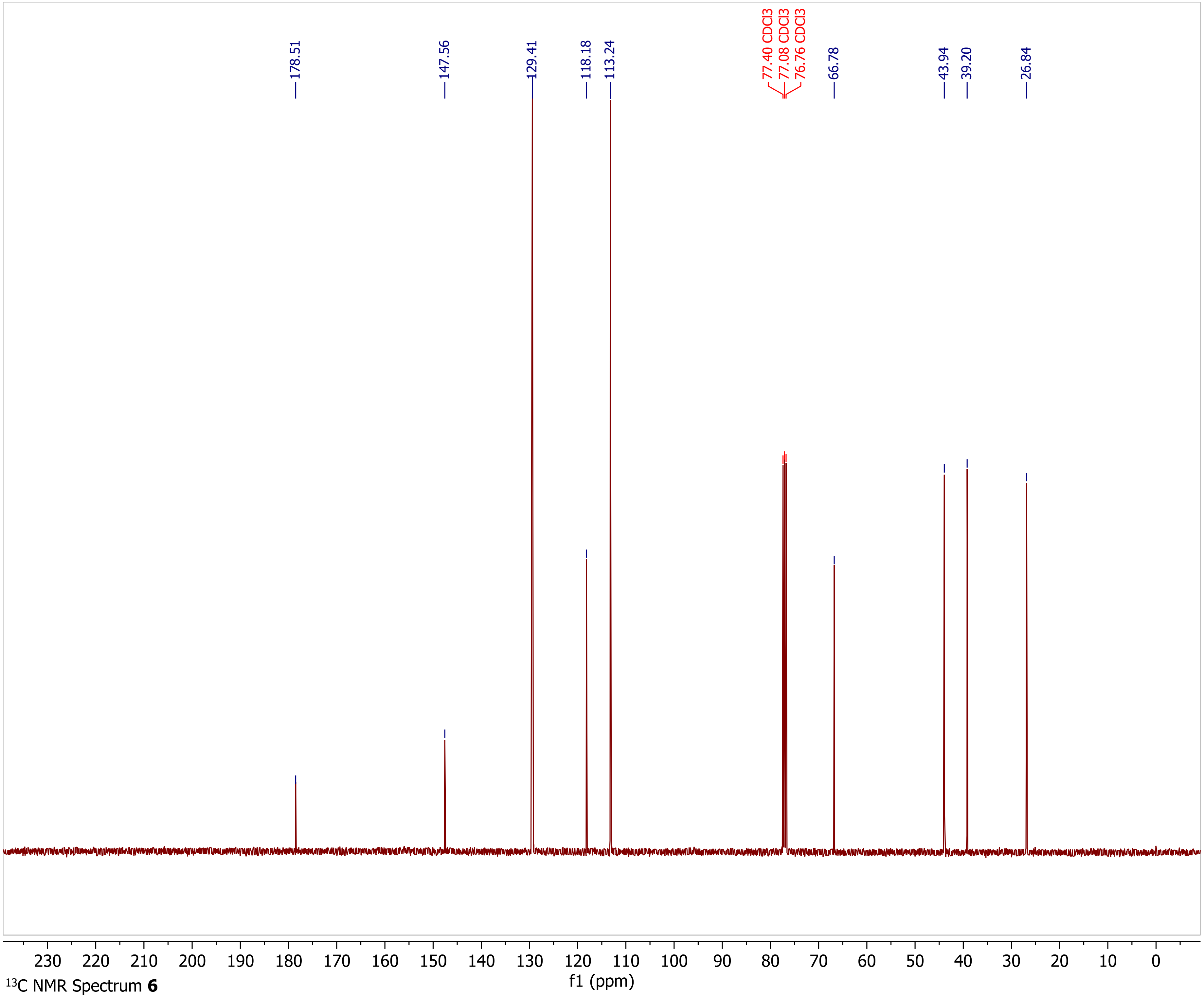

Current Data Parameters
NAME AQ-XL-6-C
EXPNO 10
PROCNO 1

F2 - Acquisition Parameters
Date_ 20200127
Time 2.57 h
INSTRUM AvanceNeo
PROBHD Z116098_0793 (
PULPROG zgpg30
TD 119044
SOLVENT CDCl3
NS 512
DS 0
SWH 25000.000 Hz
FIDRES 0.420013 Hz
AQ 2.3808801 sec
RG 35.1563
DW 20.000 usec
DE 7.12 usec
TE 298.0 K
D1 1.00000000 sec
D11 0.03000000 sec
TD0 1
SFO1 100.6243390 MHz
NUC1 13C
P0 3.33 usec
P1 10.00 usec
PLW1 83.92700195 W
SFO2 400.1318006 MHz
NUC2 1H
CPDPRG[2 waltz64
PCPD2 90.00 usec
PLW2 18.69700050 W
PLW12 0.20396000 W
PLW13 0.10259000 W

F2 - Processing parameters
SI 131072
SF 100.6127685 MHz
WDW EM
SSB 0
LB 1.00 Hz
GB 0
PC 1.40

^{13}C NMR Spectrum **6**

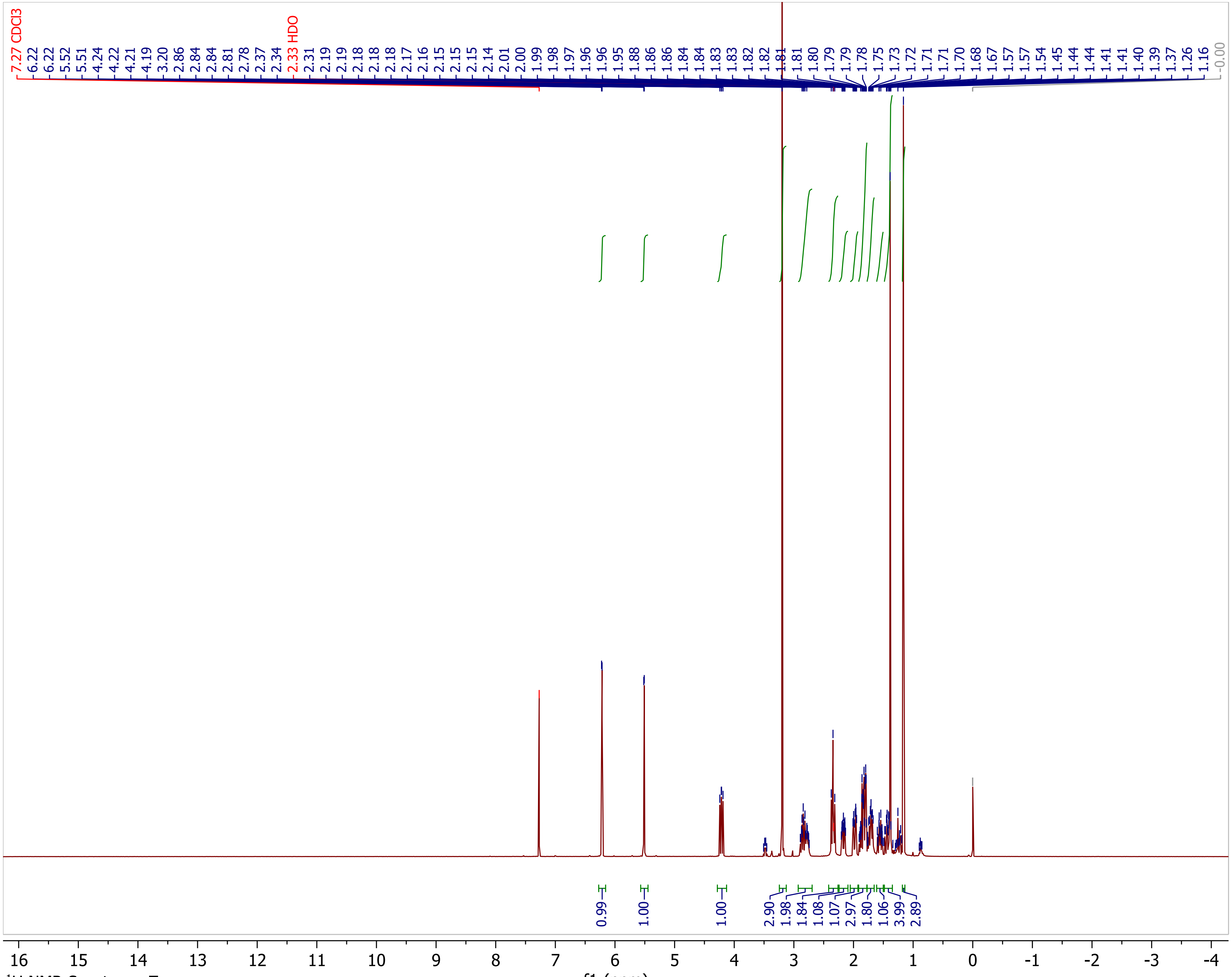

Current Data Parameters
NAME 2017-Oct-28-Fossey-15
EXPNO 10
PROCNO 1

F2 - Acquisition Parameters
Date_ 20171028
Time 14.26
INSTRUM spect
PROBHD 5 mm PADUL 13C
PULPROG zg30
TD 32768
SOLVENT CDCl3
NS 32
DS 2
SWH 8223.685 Hz
FIDRES 0.250967 Hz
AQ 1.9922944 sec
RG 203
DW 60.800 usec
DE 16.65 usec
TE 295.1 K
D1 1.50000000 sec
TD0 1

======== CHANNEL f1 ========
SFO1 400.1324008 MHz
NUC1 1H
P1 11.06 usec
PLW1 24.29199982 W

F2 - Processing parameters
SI 32768
SF 400.1300055 MHz
WDW EM
SSB 0
LB 0.30 Hz
GB 0
PC 1.00

^{1}H NMR Spectrum **7**

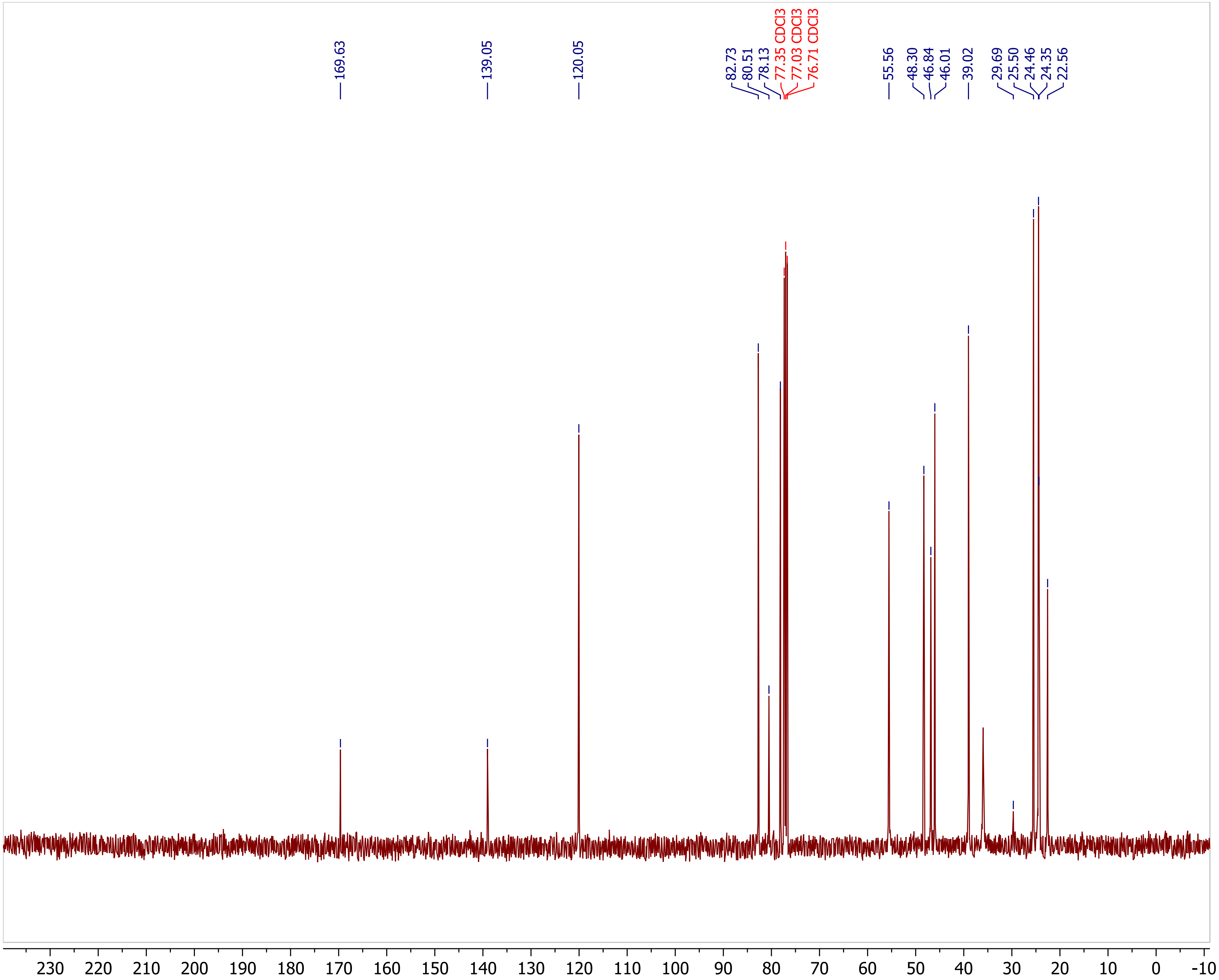

^{13}C NMR Spectrum **7**

Current Data Parameters
NAME 2017-Oct-28-Fossey-15
EXPNO 12
PROCNO 1

F2 - Acquisition Parameters
Date_ 20171028
Time 15.03
INSTRUM spect
PROBHD 5 mm PADUL 13C
PULPROG udeft
TD 18178
SOLVENT CDCl3
NS 380
DS 0
SWH 25252.525 Hz
FIDRES 1.389181 Hz
AQ 0.3599244 sec
RG 2050
DW 19.800 usec
DE 8.20 usec
TE 295.5 K
D1 3.00000000 sec
D11 0.03000000 sec
D12 0.00002000 sec
D20 200.00000000 sec
TD0 380

======== CHANNEL f1 ========
SFO1 100.6242690 MHz
NUC1 13C
P1 8.80 usec
P13 2000.00 usec
P26 500.00 usec
PLW1 58.63899994 W
SPNAM[5] Crp60comp.4
SPOAL5 0.500
SPOFFS5 0 Hz
SPW5 6.93809986 W
SPNAM[8] Crp60,0.5,20.1
SPOAL8 0.500
SPOFFS8 0 Hz
SPW8 6.93809986 W

======== CHANNEL f2 ========
SFO2 400.1320000 MHz
NUC2 1H
CPDPRG[2 waltz16
PCPD2 90.00 usec
PLW2 24.29199982 W
PLW12 0.28218001 W

F2 - Processing parameters
SI 65536
SF 100.6127690 MHz
WDW EM
SSB 0
LB 2.00 Hz
GB 0
PC 1.00

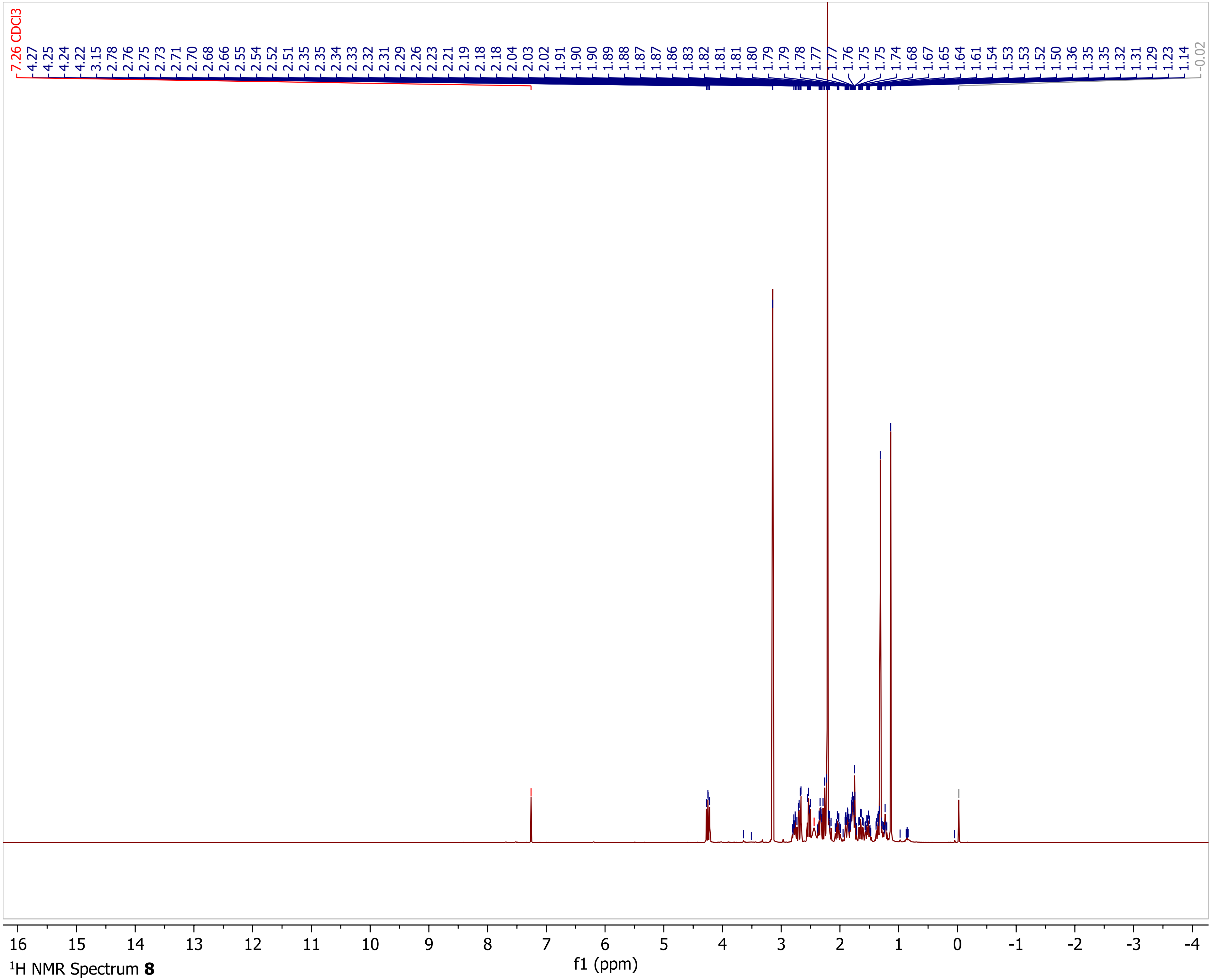

Current Data Parameters
NAME 07-29-Fossey-11
EXPNO 11
PROCNO 1

F2 - Acquisition Parameters
Date_ 20150730
Time 7.26
INSTRUM spect
PROBHD 5 mm PADUL 13C
PULPROG zg30
TD 32768
SOLVENT CDCl3
NS 32
DS 2
SWH 8223.685 Hz
FIDRES 0.250967 Hz
AQ 1.9922944 sec
RG 114
DW 60.800 usec
DE 16.98 usec
TE 293.1 K
D1 1.50000000 sec
TD0 1

======== CHANNEL f1 ========
SFO1 400.1324008 MHz
NUC1 1H
P1 9.50 usec
PLW1 24.29199982 W

F2 - Processing parameters
SI 32768
SF 400.1300004 MHz
WDW EM
SSB 0
LB 0.30 Hz
GB 0
PC 1.00

^{1}H NMR Spectrum **8**

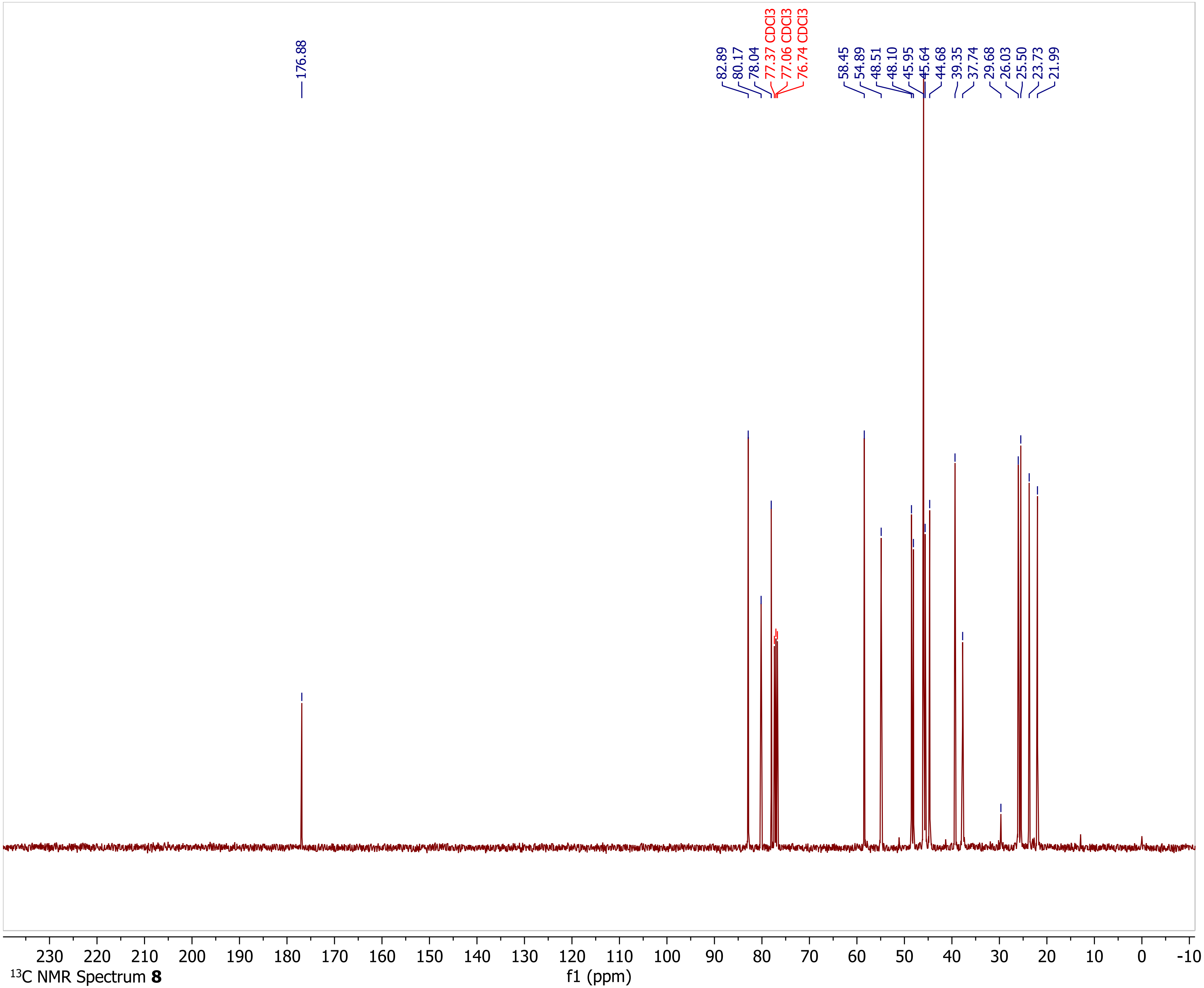

^{13}C NMR Spectrum **8**

Current Data Parameters
NAME 07-29-Fossey-11
EXPNO 15
PROCNO 1

F2 - Acquisition Parameters
Date_ 20150730
Time 9.03
INSTRUM spect
PROBHD 5 mm PADUL 13C
PULPROG udeft
TD 18178
SOLVENT CDCl3
NS 380
DS 0
SWH 25252.525 Hz
FIDRES 1.389181 Hz
AQ 0.3599244 sec
RG 2050
DW 19.800 usec
DE 8.20 usec
TE 293.2 K
D1 3.00000000 sec
D11 0.03000000 sec
D12 0.00002000 sec
D20 200.00000000 sec
TD0 380

======== CHANNEL f1 ========
SFO1 100.6242690 MHz
NUC1 13C
P1 8.80 usec
P13 2000.00 usec
P26 500.00 usec
PLW1 58.63899994 W
SPNAM[5] Crp60comp.4
SPOAL5 0.500
SPOFFS5 0 Hz
SPW5 6.93809986 W
SPNAM[8] Crp60,0.5,20.1
SPOAL8 0.500
SPOFFS8 0 Hz
SPW8 6.93809986 W

======== CHANNEL f2 ========
SFO2 400.1320000 MHz
NUC2 1H
CPDPRG[2 waltz16
PCPD2 90.00 usec
PLW2 24.29199982 W
PLW12 0.28218001 W

F2 - Processing parameters
SI 65536
SF 100.6127690 MHz
WDW EM
SSB 0
LB 2.00 Hz
GB 0
PC 1.00

Supplementary References

1 Nakayama, G. R.; Caton, M. C.; Nova, M. P.; Parandoosh, Z. *J. Immunol. Methods* **1997**, *204*, 205.
2 Dolomanov, O. V.; Bourhis, L. J.; Gildea, R. J.; Howard, J. A. K.; Puschmann, H. *J. Appl. Crystallogr.* **2009**, *42*, 339.
3 Sheldrick, G. M. *Acta Crystallogr. Sect. A* **2008**, *A64*, 112.
4 Sheldrick, G. M. *Acta Crystallogr. Sect. A* **2015**, *A71*, 3.
5 Sheldrick, G. M. *Acta Crystallogr. Sect. C* **2015**, *C71*, 3.
