# Supplementary material for: Aniline-containing derivatives of parthenolide: Synthesis and anti-chronic lymphocytic leukaemia activity
Source: Tetrahedron. 2020 Nov 27;76(48):131631. doi: 10.1016/j.tet.2020.131631 (PMC7695678; doi:10.1016/j.tet.2020.131631)

checkCIF/PLATON report

Structure factors have been supplied for datablock(s) 7

THIS REPORT IS FOR GUIDANCE ONLY. IF USED AS PART OF A REVIEW PROCEDURE FOR PUBLICATION, IT SHOULD NOT REPLACE THE EXPERTISE OF AN EXPERIENCED CRYSTALLOGRAPHIC REFEREE.

No syntax errors found.   CIF dictionary   Interpreting this report

Datablock: 7

```
Bond precision:  C-C = 0.0030 A          Wavelength=1.54184

Cell:           a=6.42575(9)    b=25.7445(3)         c=9.53536(12)
                alpha=90        beta=105.0673(13)    gamma=90
Temperature:    100 K

                 Calculated                Reported
Volume           1523.18(4)                1523.19(3)
Space group      P 21                      P 1 21 1
Hall group       P 2yb                     P 2yb
Moiety formula   2(C16 H24 O4), H2 O       2(C16 H24 O4), H2 O
Sum formula      C32 H50 O9                C32 H50 O9
Mr               578.72                    578.72
Dx,g cm-3        1.262                     1.262
Z                2                         2
Mu (mm-1)        0.741                     0.741
F000             628.0                     628.0
F000'            629.98
h,k,lmax         7,31,11                   7,31,11
Nref             5685[ 2911]               5683
Tmin,Tmax        0.897,0.968               0.967,0.993
Tmin'            0.828

Correction method= # Reported T Limits: Tmin=0.967 Tmax=0.993
AbsCorr = GAUSSIAN

Data completeness= 1.95/1.00      Theta(max)= 69.138

R(reflections)= 0.0264( 5469)     wR2(reflections)= 0.0637( 5683)

S = 1.031                  Npar= 392
```

The following ALERTS were generated. Each ALERT has the format
test-name_ALERT_alert-type_alert-level.
Click on the hyperlinks for more details of the test.

Alert level G

```
PLAT791_ALERT_4_G Model has Chirality at C4         (Sohnke SpGr)          S Verify
PLAT791_ALERT_4_G Model has Chirality at C5         (Sohnke SpGr)          S Verify
PLAT791_ALERT_4_G Model has Chirality at C6         (Sohnke SpGr)          S Verify
PLAT791_ALERT_4_G Model has Chirality at C7         (Sohnke SpGr)          R Verify
PLAT791_ALERT_4_G Model has Chirality at C11        (Sohnke SpGr)          R Verify
PLAT791_ALERT_4_G Model has Chirality at C12        (Sohnke SpGr)          R Verify
PLAT791_ALERT_4_G Model has Chirality at C104       (Sohnke SpGr)          S Verify
PLAT791_ALERT_4_G Model has Chirality at C105       (Sohnke SpGr)          S Verify
PLAT791_ALERT_4_G Model has Chirality at C106       (Sohnke SpGr)          S Verify
PLAT791_ALERT_4_G Model has Chirality at C107       (Sohnke SpGr)          R Verify
PLAT791_ALERT_4_G Model has Chirality at C111       (Sohnke SpGr)          R Verify
PLAT791_ALERT_4_G Model has Chirality at C112       (Sohnke SpGr)          R Verify
PLAT883_ALERT_1_G No Info/Value for _atom_sites_solution_primary .     Please Do !
PLAT978_ALERT_2_G Number C-C Bonds with Positive Residual Density.         17 Info
PLAT992_ALERT_5_G Repd & Actual _reflns_number_gt Values Differ by          1 Check
```

```
  0 ALERT level A = Most likely a serious problem - resolve or explain
  0 ALERT level B = A potentially serious problem, consider carefully
  0 ALERT level C = Check. Ensure it is not caused by an omission or oversight
 15 ALERT level G = General information/check it is not something unexpected

  1 ALERT type 1 CIF construction/syntax error, inconsistent or missing data
  1 ALERT type 2 Indicator that the structure model may be wrong or deficient
  0 ALERT type 3 Indicator that the structure quality may be low
 12 ALERT type 4 Improvement, methodology, query or suggestion
  1 ALERT type 5 Informative message, check
```

It is advisable to attempt to resolve as many as possible of the alerts in all categories. Often the minor alerts point to easily fixed oversights, errors and omissions in your CIF or refinement strategy, so attention to these fine details can be worthwhile. In order to resolve some of the more serious problems it may be necessary to carry out additional measurements or structure refinements. However, the purpose of your study may justify the reported deviations and the more serious of these should normally be commented upon in the discussion or experimental section of a paper or in the "special_details" fields of the CIF. checkCIF was carefully designed to identify outliers and unusual parameters, but every test has its limitations and alerts that are not important in a particular case may appear. Conversely, the absence of alerts does not guarantee there are no aspects of the results needing attention. It is up to the individual to critically assess their own results and, if necessary, seek expert advice.

Publication of your CIF in IUCr journals

A basic structural check has been run on your CIF. These basic checks will be run on all CIFs submitted for publication in IUCr journals (*Acta Crystallographica*, *Journal of Applied Crystallography*, *Journal of Synchrotron Radiation*); however, if you intend to submit to *Acta Crystallographica Section C* or *E* or *IUCrData*, you should make sure that full publication checks are run on the final version of your CIF prior to submission.

Publication of your CIF in other journals

Please refer to the *Notes for Authors* of the relevant journal for any special instructions relating to CIF submission.

PLATON version of 04/06/2020; check.def file version of 02/06/2020

Datablock 7 - ellipsoid plot

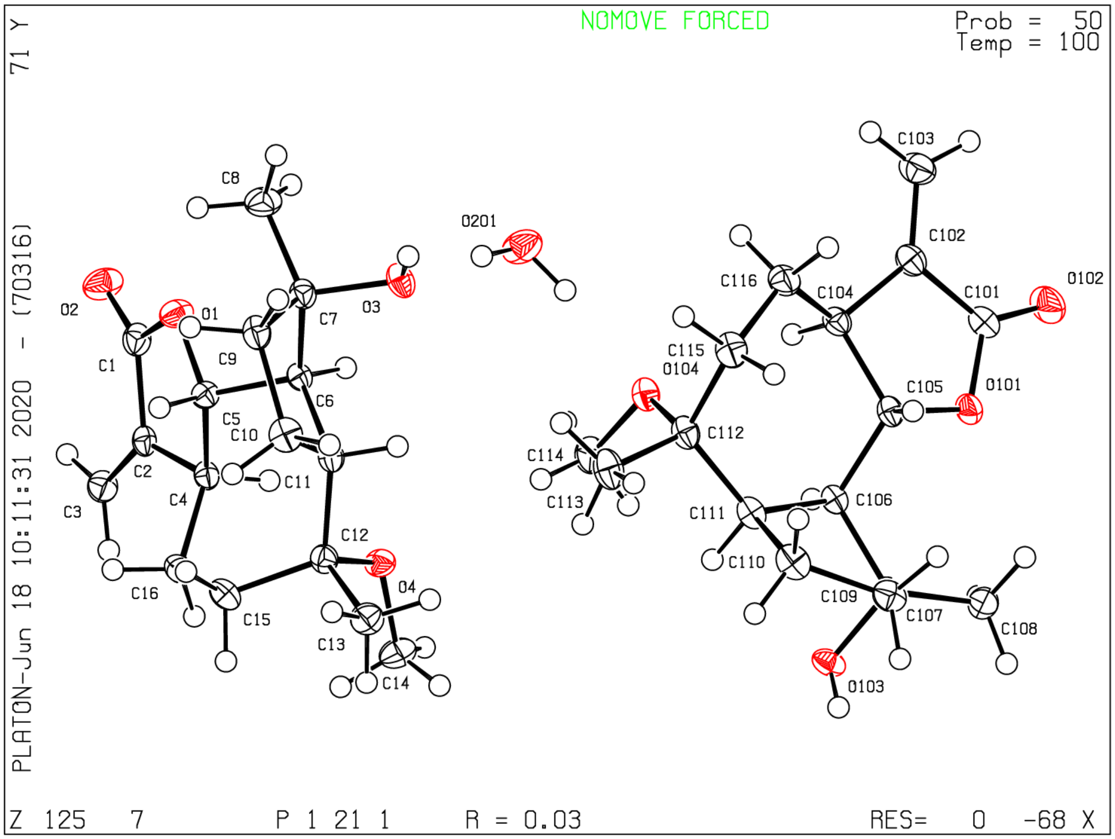

Supplement: cif check 7 [file mmc3.pdf]
